# Multiscale Modeling of the Bacterial Ribosome to Identify Potential Peptide Modulators and Their Allosteric Effects

**DOI:** 10.1021/acs.biochem.5c00832

**Published:** 2026-04-27

**Authors:** Merve Yuce, Fethiye Aylin Sungur, Ozge Kurkcuoglu

**Affiliations:** † Department of Chemical Engineering, 52971 Istanbul Technical University, Istanbul 34469, Turkey; ‡ Computational Science and Engineering Division, Informatics Institute, Istanbul Technical University, Istanbul 34469, Turkey

## Abstract

The bacterial ribosome is a key antibiotic target, yet peptide-based modulation from its functional and allosteric sites is underexplored. We developed a computational pipeline combining SiteMap-derived binding-site detection, consensus docking with Glide and rDock, all-atom truncated molecular dynamics (MD), and coarse-grained MD (CGMD) simulations to identify peptide candidates against four*E. coli*ribosomal sites: the decoding center, peptidyl transferase center, a putative binding pocket on 30S, and the intersubunit bridge B8. Consensus-selected peptides recapitulated hallmark contacts of the native inhibitors viomycin and dalfopristin, and their interaction fingerprints delineate site-specific scaffolds that enable prioritization of inhibitor candidates with enhanced ribosomal affinity, thereby guiding the rational design of novel and effective peptide-based therapeutics. Notably, the peptide CycPeptMPDB_2508 exhibited binding affinity across all investigated sites, nominating it as a versatile lead core for antimicrobial peptide design. Dynamic cross-correlation matrices derived from CGMD simulations captured coupled motions between distal regions of the ribosome, while residue interaction network analysis identified hub residues enriched near the putative binding pocket and B8 bridge, outlining putative allosteric pathways linking local pockets to global motions relevant to decoding and domain closure. This work provides a concise, testable framework for ribosome-targeted peptide discovery and, to the best of our knowledge, constitutes the first ribosome–peptide virtual screening study to employ the viparr module for truncated ribosome–peptide complexes, suggesting the potential applicability of this approach to complex systems and broadening its scope.

## Graphical abstract

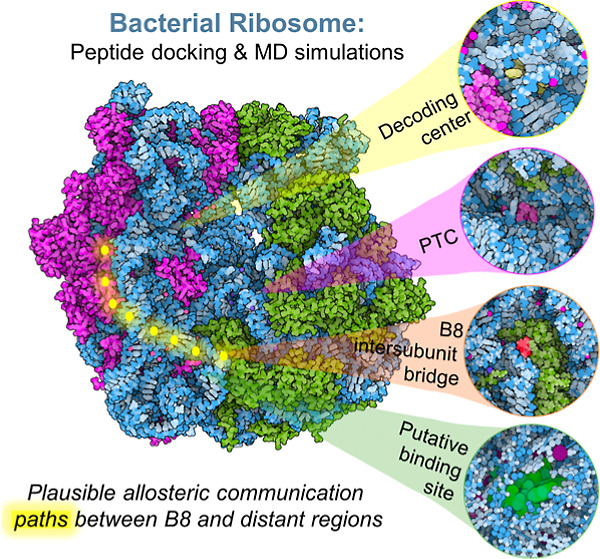

## Introduction

The bacterial ribosome is a vital target for drug discovery due to its essential role in protein synthesis and its involvement in the mechanism of action of numerous antibiotics.^1–3^ This highly complex supramolecular structure of 2.5 MDa consists of two distinct subunits: the 30S small subunit, which is composed of the 16S rRNA and over 20 associated ribosomal proteins, and the 50S large subunit, which comprises the 5S rRNA, 23S rRNA, and more than 30 ribosomal proteins.^4^ Together, these subunits assemble to form the functional 70S ribosomal complex. Key functional regions, such as the decoding center (DC) of the A-site in the 30S subunit, the peptidyl transferase center (PTC) and the nascent peptide exit tunnel (NPET) in the 50S subunit, are the primary targets of many antibiotics.^5–9^ While traditional antibiotics predominantly target these functional regions of the ribosome, some disrupt intersubunit bridges, thereby inhibiting the initiation of protein synthesis.^10,11^ Recently, novel antibiotic binding sites on bacterial ribosomes have been identified as alternative therapeutic targets, shedding light on the allosteric regulation of ribosomal functions.^12–14^ This allosteric regulation, which indirectly modulates ribosomal function,^15^ offers promising opportunities for the development of innovative therapeutics, particularly in the prevailing climate of increasing antibiotic resistance.

Peptide-based therapeutics have gained significant attention over the past several decades due to their diverse biological activities and the growing global challenge of pathogen resistance to existing drugs.^16–18^ In 2024, the peptide therapeutics market was valued at approximately 45 billion USD, underscoring the immense potential for advancing peptide drug discovery technologies. Within this context, peptide antibiotics have emerged as promising candidates for targeting functional sites on the bacterial ribosome.^19–28^ However, the development of high-affinity peptides capable of effectively binding to these regions remains a considerable challenge, primarily due to issues such as proteolytic degradation, poor membrane permeability, and low oral bioavailability.^29^ High-throughput virtual screening (HTVS) offers a promising solution to these obstacles by facilitating the identification of peptide ligands and leveraging pharmacological algorithms to optimize candidate selection.^30^

Although HTVS methods have been advanced, predicting RNA–ligand interactions remains difficult because of RNA’s intrinsic flexibility and high electronegativity.^31^ Fortunately, the growing repository of structural data available in the Protein Data Bank has provided valuable insights into the structural characteristics of RNA-binding sites while stimulating the development of RNA-specific scoring functions^32–35^ and RNA-focused docking software, such as rDock,^36^ the fragment-based approach SILCS-RNA,^37^ and the meta-dynamics enhanced-sampling method SHAMAN.^38^ Furthermore, artificial intelligence-based virtual screening tools, such as FingeRNAt,^39^ RLaffinity,^40^ and RNAmigos2,^41^ have been introduced to accelerate RNA-targeted drug discovery. Despite these advancements, the rational design of novel and effective peptide-based therapeutics to target the bacterial ribosome remains largely unexplored, mainly due to its 2.5 MDa size.

Recent progress in molecular docking methods has further expanded opportunities for peptide-based drug discovery. Among available tools, Glide^42^ has long been regarded as one of the most accurate docking programs for small molecules and has been successfully extended to accommodate flexible peptides, aided by enhanced sampling protocols and scoring improvements.^43–45^ In parallel, rDock,^36^ designed with a focus on RNA–ligand interactions, has demonstrated strength in capturing binding modes within highly charged and flexible RNA environments.^46^

The development of peptide therapeutics has been greatly facilitated by the establishment of numerous peptide databases, which serve as essential resources for researchers in the field. Among the most widely used of these is the database of antimicrobial activity and structure of peptides (DBAASP), an open-access and comprehensive repository that provides detailed information on amino acid sequences, three-dimensional structures, bioactivities, and toxicities of peptides with antimicrobial properties.^47^ Moreover, machine-learning-based tools implemented in the DBAASP database provide information for the development of predictive models for peptide toxicity, thereby addressing one of the primary obstacles to the clinical advancement of antimicrobial peptides (AMPs). On the other hand, cyclic peptides have recently emerged as a highly promising class of pharmaceutical agents, distinguished from other classes of therapeutic molecules by enhanced proteolytic resistance since both termini are absent.^48^ The CyclicPepedia^49^ and CycPeptMPDB^50,51^ databases have compiled an extensive array of data on cyclic peptides, encompassing comprehensive information on their structures, bioactivities, pharmacokinetic properties, targets, functions, and other relevant characteristics. Notably, CycPeptMPDB also includes detailed data on the cell membrane permeabilities of cyclic peptides.^50,51^ These three peptide libraries were employed in this study.

In this study, we aimed to identify hit peptides targeting functional and putative binding pockets of the*E. coli* ribosome using a combination of docking and molecular dynamics (MD) simulations and to predict their effects on the globular dynamics of the complex. In our previous study, residue network analysis identified intersubunit bridges, such as B8, as accommodating hub residues, underscoring their potential role in facilitating allosteric communication between multiple distant functional sites.^52^ Building on that, we predicted a putative allosteric site on the 30S small subunit using Gaussian Network Model fast mode analysis.^53^ To comprehensively model interactions at the ribosomal binding site, targeting not only the rRNA but also the rRNA-protein interface (B8 bridge), we employed two distinct docking programs, Glide and rDock. Glide offers robust handling of peptide conformational search and scoring, while rDock provides RNA-specific adaptation particularly suited to the ribosomal binding landscape. This consensus approach, leveraging the programs’ different search algorithms and scoring functions, avoided reliance on a single scoring function. Docking scores were ranked, and peptides with z-scores < −1.65 (the lowest 5% portion of the distribution) from both programs were selected for further analysis. The peptides identified by consensus between the two programs were further evaluated for nonbonded interactions and recurrent sequences, and the top candidates were subjected to three independent 100 ns MD simulations in four regions (two orthosteric sites, the B8 bridge, and the putative binding pocket). For each peptide candidate, an interaction fingerprint was derived by mapping contact types to the physicochemical classes of the contacting peptide residues. This framework provides a rationale for prioritizing candidates with an enhanced ribosomal affinity for *in vitro* validation. Moreover, interaction fingerprints at the B8 region served to investigate the allosteric communication coupling the docking sites around B8 and distal functional regions of the ribosome, using four independent 750 ns coarse-grained molecular dynamics (CGMD) simulations of the 70S complex combined with residue interaction network analysis.

This study provides a comprehensive computational pipeline for ribosome-targeting peptide discovery. By integrating molecular docking, consensus scoring, and MD simulations, we not only assessed the binding stability and interaction profiles of the selected peptides but also identified recurrent sequences that may guide future peptide design. Our findings contribute to the growing field of ribosome-targeted drug discovery and offer valuable insights into the development of peptide-based therapeutics targeting orthosteric and allosteric sites.

## Materials and Methods

In this study, peptide docking calculations using three peptide libraries were performed for four distinct sites on the *E. coli* ribosome: the decoding center, the peptidyl transferase center, the B8 intersubunit bridge, and a putative binding pocket near the B8 bridge. To enhance the identification of true positives, two docking programs, Glide and rDock, which employ different search algorithms and scoring functions, were utilized. Docking scores were ranked, and peptides with z-scores < −1.65 from both programs were selected for further sequence similarity analysis based on the sequences of these peptides. Subsequently, peptides identified by both programs were evaluated based on docking interactions and shared recurrent sequences, leading to the selection of hit peptides for three independent 100 ns long MD simulations. The MD simulations and subsequent analyses were carried out to assess the binding stability and interaction profiles of the selected peptides. To evaluate correlated motions between the putative binding pocket, the B8 bridge, and distal regions of the 70S complex, we constructed a dynamic cross-correlation matrix (DCCM) from the coarse-grained MD (CGMD) trajectories. Residue interaction network (RIN) analysis identified hub residues that have a high capacity to transmit an allosteric signal through correlated motions.

### Data Collection

Given the existence of crystal structures of bacterial ribosomes complexed with both linear and cyclic peptides, a diverse library was compiled. Peptides that possessed an available three-dimensional structure and sequence length of less than 15 amino acids were obtained from the DBAASP v3 (98 peptides),^54^ CyclicPepedia (778 peptides),^49^ and CycPeptMPDB (7451 peptides)^50^ databases. The crystal structures of *E. coli* ribosomes in complex with the tuberactinomycin antibiotic viomycin (PDB ID: 4v7l, 3.00 Å) and the streptogramin A antibiotic dalfopristin (PDB ID: 4u24, 2.90 Å) were used in the calculations.^55,56^ Viomycin is a cyclic pentapeptide antibiotic containing several noncanonical amino acids, and dalfopristin belongs to streptogramin A antibiotics, which are unsaturated macrolactones with peptide bonds.

### Receptor and Ligand Preparation for Molecular Docking

Peptides were prepared using the LigPrep module as implemented in Schrödinger (LigPrep, Schrödinger, LLC, New York, NY, USA), which involves energy minimization using the OPLS4 force field.^57^ Here, the ionization state of the molecules was determined at pH 7.0 ± 0.5 following physiological conditions using the Epik ionization tool (Epik module, Schrödinger Release 2024-3). Multiple conformations were produced for each peptide, yielding 13,380 conformers from the DBAASP v3 database, 2010 conformers from CyclicPepedia, and 7485 conformers from CycPeptMPDB. All generated conformations were subsequently used in the peptide docking calculations.

The *E. coli* 30S ribosome small subunit (PDB ID: 4v7l) in complex with viomycin^55^ was prepared for peptide docking calculations at the decoding center (DC) and the putative binding pocket, whereas the 50S large subunit (PDB ID: 4u24) in complex with dalfopristin^56^ was prepared for docking calculations at the peptidyl transferase center (PTC) using the Protein Preparation Wizard (Schrödinger Release 2024-3). Crystal waters, Mg^2+^ ions, and all ribosomal proteins were maintained in the structures. The *E. coli* 70S ribosome (PDB ID: 4v7l) was prepared for peptide docking calculations of the B8 bridge. Crystal waters and Mg^2+^ ions were maintained in the structure, whereas all ribosomal proteins except those close to the binding site (s20, L14, and L19) were removed. Missing hydrogen and side chain atoms were added to the Protein Preparation Wizard module. PROPKA was employed to adjust the protonation states of amino acids, while Epik was utilized to predict the ionization and tautomeric states of the cocrystallized ligands under the physiological pH of 7.0. Subsequently, the 30S small subunit, 50S large subunit, and 70S ribosomal complex were subjected to energy minimization under the OPLS4 force field to eliminate steric overlaps resulting from the addition of hydrogens to the structure. Energy minimization was carried out using the Impref module,^58^ where the root mean squared deviation (RMSD) tolerance for heavy atoms was set to 0.3 Å. The prepared 30S ribosome small subunit and 50S ribosome large subunit consisted of approximately 88,000 and 153,000 atoms, respectively.

### Binding Pocket Detection Using SiteMap

Due to the absence of peptide-bound crystal structures for the B8 intersubunit bridge and the proposed allosteric site, potential binding cavities within these regions were identified using the SiteMap module^59,60^ implemented in Schrödinger Release 2024-3. SiteMap can be used to identify and characterize potential ligand binding sites on protein or DNA/RNA receptors. In this study, the putative binding pocket was analyzed on the 30S subunit of the *E. coli* ribosome crystal structure (PDB ID 4v7l), whereas the B8 intersubunit bridge was analyzed on the intact 70S *E. coli* ribosome (PDB ID 4v7l). For this analysis, key nucleotides^53^ given in Table S1 previously determined through the Gaussian Network Model were specified as reference points for the putative binding site. The search for ligand-binding cavities was conducted by defining a grid encompassing a 25 Å region centered at the site (Figure S1a; center coordinates: *x* = 63.5 Å, *y* = −1.0 Å, and *z* = 69.3 Å) corresponding to these nucleotides. For the B8 intersubunit bridge, the grid was centered within a 40 Å radius of the U340 nucleotide (Figure S1b; center coordinates: *x* = 68.7 Å, *y* = −21.1 Å, and *z* = 61.9 Å), a critical residue in helix h14 of 16S rRNA that contributes to the formation of the B8 bridge.^61^ To improve the prediction of peptide-binding sites, the enclosure and maxvdw parameters were set to 0.4 and 0.55, respectively.^42^ Charge density calculations for the rRNA receptor were performed using the OPLS4 force field.^57^

Druggable sites were identified based on SiteScore and DScore metrics. SiteScore is an empirical function that incorporates a weighted sum of hydrogen bonding, enclosure/exposure, contact, and hydrophilic/hydrophobic terms. For DScore, the hydrophilic score is not capped, which is a critical factor in distinguishing undruggable targets from druggable ones.^60^ The use of distinct functions for binding site identification (SiteScore) and druggability classification (DScore) is justified by their differing characteristics and the nature of the interactions that they assess. Binding sites with SiteScore and DScore values greater than 1.0 were prioritized and selected for subsequent peptide screening.

### Peptide Docking Calculations

Two docking programs, Glide^42^ and rDock,^36^ were employed to decide on the hit compounds following the consensus of two different scoring functions. The standard precision peptide mode (SP-Peptide) of Glide was employed to dock the antimicrobial peptides to the targeted regions of the bacterial ribosome.^42^ The SP-Peptide mode of Glide is specifically optimized to more accurately predict the binding affinity and binding poses of polypeptides. The Receptor Grid Generation module (Schrödinger Release 2024-3) was used to determine the grid box size with standard parameters optimized for peptide docking. The grid box coordinates were centered at the native ligand viomycin on DC (*x* = 104.8 Å, *y* = 9.4 Å, and *z* = 21.7 Å). For PTC, the grid box was centered at the cocrystallized ligand dalfopristin (*x* = −77.5 Å, *y* = −52.3 Å, and *z* = 2.3 Å). For the putative binding pocket and B8 bridge, the center of the grid box was set to *x* = 63.5 Å, *y* = −1.0 Å, and *z* = 69.3 Å and *x* = 65.1 Å, *y* = −30.9 Å, and *z* = 59.8 Å, respectively. The outer grid box was sized as 25.0 × 25.0 × 25.0 Å^3^, while the inner grid box was 10.0 × 10.0 × 10.0 Å^3^. During the docking calculations performed with the OPLS4 force field,^57^ the dihedral angles of the compounds were kept flexible while the receptor (including 23S and 5S rRNA, 16S rRNA, ribosomal proteins, crystal waters, and crystal Mg^2+^ ions) was kept rigid. In the validation step, we first tested the suitability of Glide with SP-Peptide mode accuracy to predict the positions of the cocrystallized inhibitors, viomycin and dalfopristin. Validation of the docking protocol was further performed using cross-docking with capreomycin (PDB ID: 4V7M). The detailed protocol and results are given in the Supporting Information.

For the consensus docking approach, the rDock program—specifically designed for RNA targets—was also employed in the docking studies of peptides.^36,62^ For cavity generation in DC and PTC regions, the reference ligand method was employed, using the position of the cocrystallized peptide inhibitor as the reference for cavity mapping. The default mapping algorithm, RbtLigandSiteMapper, was used with the cavity mapping region radius set to 10.0 Å and the small probe radius set to 1.5 Å. In the case of putative binding sites and B8 bridges, the two-sphere method was used for cavity mapping. Here, the RbtSphereSiteMapper mapping algorithm was applied with the small and large probe radii set to 1.5 and 4 Å, respectively, to define the docking cavity. The cavity mapping region radius was set to 20.0 Å. The maximum number of cavities to accept was set to 1. For scoring and ranking the docking poses, the standard scoring function (dock.prm) implemented in rDock was used, incorporating intermolecular, intraligand, and intratarget terms. The solvation term was not included in the scoring function.

For all docking calculations performed with both Glide and rDock, 100 poses were generated for each peptide to ensure comprehensive sampling of the binding pockets. The top-ranked pose based on the lowest docking score was selected for further analysis.

### All-Atom Molecular Dynamics Simulations

The 70S ribosomal complex comprises over 240,000 atoms, rendering it computationally impractical to investigate the entire structure using molecular dynamics (MD) simulations with an explicit solvent model at atomic resolution. To ensure computational efficiency, MD simulations were conducted on a truncated, spherical region of the bacterial ribosome, centered on each target site, as in previous studies.^63–67^ These simulations were subsequently utilized to evaluate the docking poses of the selected peptides at their respective binding sites and to analyze their interactions with specific nucleic acid or amino acid residues. MD simulations were carried out at four target sites: (1) the DC, where viomycin is bound; (2) the PTC, where dalfopristin is bound; (3) a potential allosteric site located near helices h14 and h15; and (4) the B8 intersubunit bridge region. For the second site, the truncated model was derived from the *E. coli* 50S ribosome large subunit (PDB ID: 4u24) in complex with dalfopristin,^56^ whereas for the remaining target sites, truncated models were generated from the *E. coli* ribosome structure (PDB ID: 4v7l) in complex with viomycin.^55^ A spherical region with a 60 Å radius, centering the binding site, was truncated from the ribosome structure to include both rRNA and ribosomal proteins, following the approach of previous studies.^64–66^ The 60 Å cutoff was selected as a relatively large distance to ensure the inclusion of electrostatic interactions between the peptides and the ribosome. Missing nucleotides in the rRNA and ribosomal protein chains were completed, while chains shorter than five residues or nucleotides were removed to generate a truncated, globular region. Mg^2+^ ions and crystallographic water molecules were retained within the truncated region. Truncated ribosomal structures in complex with the docked peptides were prepared in the Protein Preparation Wizard in Maestro (Schrödinger Release 2020-4). Further details regarding the resulting truncated structures are provided in Table S2.

The Desmond package^68^ was used in the MD simulations of the ribosome–peptide complexes. The simulation system was prepared using Desmond’s System Builder module (Schrödinger Release 2020-4). The TIP3P water model^69^ and a cubic water box with a buffer distance of 15 Å were used. K^+^ ions sufficient to neutralize the charge of the system were added, and KCl salt was introduced to set the ionic strength to 0.15 M to mimic the physiological conditions. The Desmond Viparr tool was used to assign rna.DES-Amber_pe3.2 force field parameters^70^ to rRNA, aa.DES-Amber_pe3.2 force field parameters^70^ to proteins, and the general Amber force field (GAFF) parameters^71^ for the nonstandard amino acids. The Mg^2+^ ions were parametrized using the ions.amber1234lm_anton.tip3p force field^70^ via the Viparr tool, which is optimized for accurate ion–nucleic acid interactions. For the ligand parametrization, the antechamber and parmchk2 modules of the AmberTools24 software package^72^ were used with AM1-BCC charges.^73^

The long-range interactions were estimated using the Particle Mesh Ewald (PME) method^74^ with a grid spacing of 0.8 Å. A 12 Å cutoff radius was set for short-range electrostatic interactions. The equations of motion were integrated using the multistep RESPA integrator^75^ with an inner time step of 2 fs for bonded interactions and nonbonded interactions with a cutoff of 9 Å. An outer time step of 6.0 fs was used for nonbonded interactions beyond the cutoff. MD simulations were performed by using a modification of the default relaxation protocol provided by Desmond. The protocol consists of a series of restrained minimizations and MD simulations designed to relax the system. First, steepest descent minimization of 2000 steps was applied using Brownian dynamics. Then, a 12 ps simulation was performed at a temperature of 10 K in the *NVT* ensemble. For the third step, the Langevin thermostat and the Langevin barostat were used at 10 K and 1 atm in the *NPT* ensemble for 12 ps. In the fourth step, the temperature was raised to 310 K under the same conditions. In the first four steps of the relaxation protocol, a harmonic constraint of 50 kcal/mol·Å^2^ was applied to all solute atoms in the structure, including the chelated Mg^2+^ ions. The heating was then followed by an equilibration simulation of 24 ps using the same parameters except for the harmonic restraints. The truncated structure was divided into three zones according to the proximity of the binding site. The middle zone (24–32 Å) and the outermost zone (32–60 Å) were restrained with 25 kcal/mol·Å^2^ and 50 kcal/mol·Å^2^, respectively. These harmonic restraints were applied to prevent the truncated structure from disintegrating during the simulation. The innermost zone (0–24 Å), including the targeted nucleotides, Mg^2+^ ions, and ligand, was kept flexible to observe the molecular interactions. The production runs were performed for 100 ns in three replicas. A Langevin thermostat and a Langevin barostat in the *NPT* ensemble were used to set the temperature and the pressure at 310 K and 1.013 bar, respectively. The harmonic restraints for the middle and outer zones were also applied during the production run.

### Coarse-Grained Molecular Dynamics Simulations

The apo crystal structure of the ribosomal complex 70S of *E. coli* with PDB ID 4V5H (containing P-tRNA and mRNA) of resolution 5.80 Å^76^ was employed in coarse-grained molecular dynamics (CGMD) simulations to investigate the intrinsic dynamics and structural flexibility of the bacterial ribosome at microsecond scales. CGMD simulations were performed using RedMD,^77^ which is suitable to study the dynamics of the ribosomal complexes.

The force fields implemented in RedMD are based on elastic network models and their extensions, such as those initially developed for ribosomes and other large biomolecular complexes. The simulations primarily sample fluctuations within the native basin of the functional complex and are not intended to capture large-scale conformational transitions or barrier-crossing events, which would be expected from multibasin structure-based models such as SMOG.^78^ Assuming that a perturbation at a site can be transmitted through coupled residue fluctuations at the low-frequency dynamics,^79^ this study aims to reveal which distant regions of the ribosome complex are dynamically coupled to the putative binding site near the B8 bridge. At this point, RedMD can reveal the collective motions of the bacterial ribosome relevant to its allosteric mechanisms^80^ and thus show how local perturbations at a putative binding site may propagate through the ribosome’s intrinsic dynamic network at a specific conformational state with high computational efficiency. Here, the intact 70S structure was described as a one-bead-per-residue model, where pseudoatoms are located at Cα and P atoms to represent residues and nucleotides, respectively. The total potential energy of the structure is given by

1 $$E=E_{1-2}+E_{1-3}+E_{1-4}+E_{bp}+E_{nonbonded}$$

The harmonic potentials *E*_1–2_, *E*_1–3_, and *E*_1–4_ account for the pseudobond, pseudoangle, and pseudodihedral interactions involving two, three, and four successive beads, respectively. *E*_bp_ indicates the harmonic interactions between the nucleic acid base pairs, identified by RedMD, which takes equilibrium distances from the initial crystal structure (PDB ID: 4V5H). The *E*_nonbonded_ energy term represents the Morse potential to determine nonbonded interaction energy, considering anharmonicity as

2 $$V\left(r\right)=A_{P,C_{a}}\left(r_{0}\right){\left[1-\exp\left(-a\left(r-r_{0}\right)\right)\right]}^{2}$$

*V*(*r*) is used for both nonbonded local and nonlocal interactions. The local terms were calculated within a cutoff distance, *R*_cutoff_, which was set as 12.0 Å for C_α_ and 20.0 Å for P atoms. For the nonlocal terms, a cutoff distance of 35.0 Å was taken for all nodes. For local interactions, *r*_0_ was taken as the equilibrium distance in the native structure, while for nonlocal interactions, it changes according to the node type. *A*_P,Cα_ is an exponential function, which differs for P···P, C_α_···C_α_, and P···C_α_ interactions and decreases with increasing distance between pseudoatoms. All parameters employed in this work are detailed in our previous study.^80^ In order to account for the solvent–ribosomal complex 70S interactions, Langevin dynamics were applied by adding viscous and random forces to Newton’s equation of motion. Prior to simulations, each system was subjected to an energy minimization as implemented in RedMD. Each system was heated from 10 to 300 K, and then production simulations were run at 300 K with a collision frequency of 2 ps^–1^ for Langevin dynamics. The MD simulations were performed for 37.5 million steps with an integration time step of 0.02 ps, resulting in a nominal trajectory of 750 ns for each of the four independent runs of the *E. coli* 70S ribosomal complex. Due to the smoothing of the potential energy landscape and the reduction of atomic degrees of freedom, CG models typically exhibit accelerated configurational sampling compared to all-atom dynamics.^81^ Consequently, the effective physical time scale sampled by the model may be significantly longer than the nominal integration time.

### Dynamic Cross-Correlation and Network Analysis

Due to its high-amplitude motions, the L1 stalk was excluded from the concatenated CGMD trajectory (comprising four independent runs), and the frames were aligned to the initial structure of the 50S core atoms. Principal component analysis (PCA) was then performed on the 50S-core-aligned concatenated trajectory using the Bio3d software tool.^82^ Residue positional fluctuations were constructed using the first ten principal components (PCs), and a dynamic cross-correlation matrix was calculated from these fluctuations to capture coupled dynamics within the apo *E. coli* 70S ribosome. Cross-correlations between each residue (*i*,*j*) pair, denoted as *C*_*ij*_, were calculated for the concatenated trajectory using the following equation:^83^

3 $$C_{ij}=C\left(\Delta R_{i},\Delta R_{j}\right)=\frac{\left\langle \Delta R_{i}\left(t\right)\cdot \Delta R_{j}\left(t\right)\right\rangle }{\sqrt{\left\langle {\left(\Delta R_{i}\right)}^{2}\right\rangle \left\langle {\left(\Delta R_{j}\right)}^{2}\right\rangle }}$$

The dot product of fluctuation vectors, Δ*R*_*i*_ and Δ*R*_*j*_, representing deviations of two distinct residues from their mean positions, was averaged across the concatenated trajectories of four CGMD simulations, respectively. Resulting cross-correlation values, ranging from −1 to 1, indicate negative correlation and positive correlation when below −0.4 or above 0.4, respectively.

The cross-correlation values were recalculated for each subunit after aligning the concatenated trajectory with the initial structure of the small subunit 30S and then with the large subunit 50S to isolate their internal conformational changes. Cross-correlation values were used to build two separate residue interaction networks for 30S and 50S consisting of nodes and edges.^83^ Nodes were placed at C_α_ and P atoms, and the node pairs within a cutoff distance, which is 10.0 Å for C_α_···C_α_, 15.0 Å for C_α_···P, and 20.0 Å for P···P atom pairs, were connected with edges.^84^ The length of edges between two neighboring nodes was defined as −ln­(*abs*(*C*_*ij*_)).^85^ For each network, the betweenness centrality metric was calculated to identify the hub residues, which are highly visited by the shortest paths. The hub residues have a high capacity for transmitting a perturbation, such as ligand binding, to distant parts of the protein structure through correlated motions. The calculation of the betweenness (*C*_B_) value is as follows:

4 $$C_{B}\left(l\right)=\sum_{i\neq j\neq l}\frac{s_{ij}\left(l\right)}{s_{\left(ij\right)}}$$

*s*_(*ij*)_ represents the total number of shortest paths between nodes *i* and *j*, while *s*_*ij*_(*l*) denotes the number of shortest paths passing through node *l*. The construction of the residue network and the calculation of betweenness centrality (*C*_B_) were performed using in-house scripts.

As two distant regions on a protein complex can communicate through coupled motions of the residues,^86^ then these regions are connected at least through one residue pathway—the shortest path. Here, the RIN model was constructed based on the low-frequency motions of the nucleotides/residues. It provided a suitable framework to investigate the importance of the nodes in the flow of information between functional sites of the bacterial ribosome using low-frequency collective motions. Assuming that the edges are undirected and weighted based on their pairwise cross-correlations, the efficiency of the shortest path *E*_*ij*_ between two nodes *i* and *j*, each located at a distinct region, can be calculated as^87,88^

5 $$E_{ij}=\frac{1}{\sum_{l=1}^{k-1}w_{l,l+1}}$$

where *w*_*l*,*l+1*_ is the edge weight between two sequential nodes *I* and *l* + *1* on the path formed of k nodes. Here, the edge weight *w*_*l*,*l+1*_ is calculated as −ln­(|*C*_*l*,*l*+1_|).

To reveal the effect of a node on the flow of information, the edge weight of the node was set to a high value, obtaining a perturbed network. To quantify the effect of the perturbation, an allosteric coupling change *C_ac,_*_*ij*_ (%) was calculated using the difference between perturbed and unperturbed states weighted by the unperturbed state.^89^ Here, a negative value in *C*_*ac,ij*_ (%) indicates a reduced level of communication of the *i*–*j* pair in the perturbed network.

On the other hand, the local efficiency *E*_loc_ of a node measures the fault tolerance and communication efficiency within its immediate neighborhood. It is defined as the global efficiency of the subgraph *G*_*i*_ formed by the neighbors of node *i*, excluding the node itself^87^ from the network of *N* nodes as

6 $$E_{loc}=\frac{1}{N}\sum_{i\neq j}E\left(G_{i}\right)$$

A high *E*_loc_ indicates a hub’s neighbors are well-connected to each other, making that part of the network more redundant and resistant to errors. On the other hand, a low *E*_loc_ identifies a critical bridge—a residue whose removal would leave its neighbors largely disconnected from one another.

The global efficiency (*E*_glob_) metric was used to quantify how efficiently information is exchanged over the entire network. It is defined as the average inverse shortest-path length between all pairs of nodes in the network as^87^

7 $$E_{glob}=\frac{1}{N\left(N-1\right)}\sum_{i\neq j}\frac{1}{d\left(i,j\right)}$$

where *d*(*i*,*j*) is the shortest path distance between nodes *i* and *j*.

## Results and Discussion

Peptide docking calculations were performed at the decoding center (DC) using the individual *E. coli* 30S ribosome small subunit (PDB ID: 4v7l) in complex with viomycin^55^ and at the peptidyl transferase center (PTC) using the individual 50S large subunit (PDB ID: 4u24) in complex with dalfopristin.^56^ It is worth noting that the docking calculations for the DC region involved only the small subunit 30S, but not the 70S complex. While the DC region is close to 23S rRNA of the 50S large subunit, we aimed to inhibit the assembly of the subunits into a functional complex. Since there is no known peptide bound at the B8 intersubunit bridge region, the SiteMap module within the Schrödinger Suite was employed to predict binding cavities in the proposed binding pocket and B8 intersubunit bridge (Figure 1) using the nonrotated state of the bacterial ribosome (PDB ID: 4v7l). Here, the aim was to identify peptides that could potentially inhibit translocation by stabilizing the classical conformation. Among the four predicted sites (Table 1 and Figure S1a), Site 1 was selected as the putative binding pocket based on its SiteScore, the size of the predicted binding pocket, and its DScore. The selected pocket exhibited a SiteScore of 1.119, indicative of a strong propensity for ligand binding. Additionally, a high DScore of 1.003 suggested that this site is highly druggable, providing a favorable environment for compounds to bind with high affinity and selectivity. Notably, the residues comprising the proposed binding pocket corresponded to those identified as part of Site 1 in the SiteMap analysis, further supporting its selection for the present docking studies. For the B8 intersubunit bridge, SiteMap predicted a binding site with a SiteScore of 1.105 and a DScore of 1.017 to be further used in docking calculations (Figure S1b).

**Figure 1:**
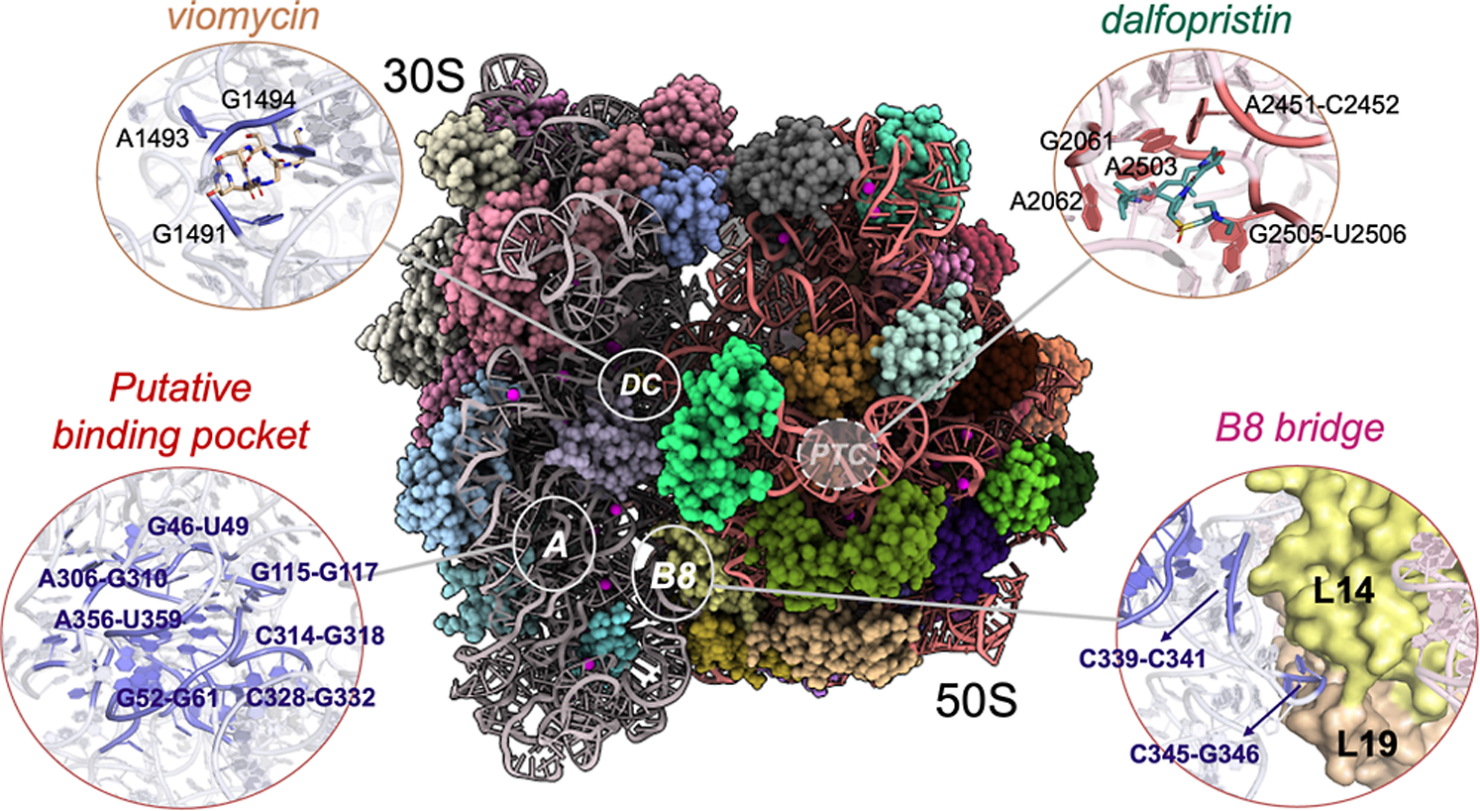
A cartoon representation of the *E. coli* 70S ribosome (PDB ID: 4v7l) is shown, with the small (30S) and large (50S) subunits distinctly displayed along with their associated ribosomal proteins. Four target sites are indicated on the structure: the decoding center (DC) on the 16S rRNA, the peptidyl transferase center (PTC) on the 23S rRNA, the putative binding pocket on the 16S rRNA, and the B8 intersubunit bridge. Magnesium ions present in the crystal structure are depicted as magenta spheres.

**Table 1:** SiteMap Results for the Putative Binding Pocket and B8 Bridge of the *E. coli* Ribosome

target site	site no	residues/nucleotides	SiteScore	size (A^3^)	DScore
putative binding pocket	Site 1	Chain A: 31,46–54,108–115,289–290,305–316,327,329–331,351–361,366–367,369–370,388–395,1611,1625,1632,1653,1677; Chain P: 25–27	1.119	1119	1.003
	Site 2	Chain A: 51–60,315–320,328–339,350–355,1625; Chain T: 7–10	1.230	540	1.034
	Site 3	Chain A: 59–60,110–112,309,330,354,376–378,387–390; Chain P: 24–29	1.147	85	0.912
	Site 4	Chain A: 60,107–111,327,329–332,1632; Chain T: 10,12–13,15	1.042	40	0.514
B8 bridge	Site 1	Chain A: 47,50–61,107–111,114–117,158–164,314–320,327–362,366,368,1432–1433,1468–1471,1604,1611,1616,1625,1632; Chain T: 7–13,15,16; Chain k: 9–17,47–52,64,80–81,91–100,107,110–122; Chain p: 35–36,41–46,65–71	1.105	3975	1.017

### Molecular Docking Calculations

#### Decoding Center

In the validation step of the docking protocol for Glide and rDock, we first redocked the cocrystallized peptide inhibitor viomycin to its binding site in the crystal structure (PDB ID: 4v7l)^55^ shown in Figure 1. The docking scores for viomycin were determined as −7.78 kcal/mol (SP-Peptide in Glide) and −36.49 kcal/mol (rDock) for its redocked poses with low root mean squared deviation (RMSD) values of approximately 1.0 Å from the crystal pose (Figure S2). The interactions of viomycin with the 16S rRNA in the crystal structure were visualized as a 2D diagram using Discovery Studio Free Visualizer 2024, indicating that the peptide kept crystal interactions within the DC with nucleotides G1491, A1493, and G1494 ^10,55^ via hydrogen bonding, salt bridges, and attractive charges (Figure S2a,b). These results confirmed the settings in the docking programs.

Peptide docking calculations were conducted for approximately 23,000 conformers from the DBAASP v3, CyclicPepedia, and CycPeptMPDB databases on the DC region of the *E. coli* 30S ribosome small subunit (PDB ID: 4v7l) using Glide SP-Peptide and rDock programs. The workflow summarizing the virtual screening of three peptide libraries against the active (DC and PTC regions) sites, the putative binding pocket, and the B8 intersubunit bridge of the bacterial ribosome is given in Figure S3. Two docking programs generated differing amounts of conformers, reflecting the differences in their underlying conformational sampling methodologies. The distributions of Glide GScore and rDock docking values (in kcal/mol) in the DC region are depicted as violin plots in Figures S4a and S4b, respectively. The Glide GScore values for the DBAASP v3 data set displayed a broader distribution and a lower median, indicating generally more favorable binding affinities relative to the other data sets. In contrast, the rDock results indicated that DBAASP v3 peptides exhibited a wider range of scores but possessed a median with a less favorable score compared to those from the CyclicPepedia and CycPeptMPDB data sets.^90^

Peptide conformers from both programs were filtered with a threshold z-score value of −1.65. Then, collected potential hit peptides were selected based on the consensus of two programs, as in our previous study on the bacterial ribosome.^66^ This approach was preferred over cross-rescoring strategies, as consensus docking approaches are known to provide more robust predictions.^91,92^ The calculations yielded 20 hit peptides from the DBAASP v3 library—including viomycin—targeting the DC region with sequence lengths ranging from 6 to 15 amino acids, the majority of which comprised 10 amino acids. The obtained peptides, along with their names, database IDs, amino acid sequences, SP-Peptide GScore, and rDock docking scores, are listed in Table S3. The analysis of the peptide sequences identified as potential hits for the DC region revealed the presence of several recurrent sequences. Notably, the 4-mer “YVXL” sequence appeared consistently in the tyrocidine A antibiotic (PDB ID 4M6E)^93^ and its synthetic analogues (PDB IDs 6B34 and 6B35).^94^ Similarly, the 3-mer “FfN” sequence was recurrently identified in this group of peptides. Additionally, the 3-mer “DSF” and “GLM” were observed in lasso peptide chaxapeptin (PDB ID 2N5C),^95^ neurokinin 1 receptor agonist uperolein (PDB ID 2GFR),^96^ and tachykinin peptide neurokinin A (PDB ID 1N6T).^97^ The 2-mer “LI” and “RK” were noted particularly in the antifungal peptide *Cm*-p1 and its derivative *Cm*-p5 (PDB IDs 6CTG and 2MP9),^98,99^ leishmanicidal peptide decoralin-NH_2_ (PDB ID 2N9A),^100^ designed peptide VG13P (PDB ID 5WRX),^101^ and TetraF2W-RK (PDB ID 6NM2).^102^ The frequent occurrence of these sequences across diverse peptide scaffolds suggested that they may contribute to the binding affinity and specificity of the peptides for the DC region.

From the second peptide library CyclicPepedia, a total of 150 peptide conformers (*z* ≤ −1.65) were obtained (Figure S3), where the sequence lengths varied between 2 and 9 amino acids. Based on consensus docking, 7 peptides were obtained, and amino acid sequences and docking scores are listed in Table S4. The “FC” and “YA” sequences were noted in the hit peptides with database IDs CP01246, CP01126, CP01663, CP00080, and CP01660. For the CycPeptMPDB database, a total of 1696 hits were identified from the docking results of both programs (Figure S3), with peptide sequence lengths ranging from 3 to 13 amino acids. Table S4 presents the amino acid sequences and corresponding docking scores of the 5 hit peptides that demonstrated the highest docking scores in both docking programs.

The interactions of the hit peptides with the DC cavity were displayed in 2D diagram maps using Discovery Studio Visualizer and are given in Figures S5–S8. The stacked bar plots summarize the number and type of interactions—such as hydrogen bonds, π-interactions (including π-sigma, π-anion, π-cation, π-sulfur, π–π stacked, and π-alkyl), and salt bridges—formed by nucleotides C1402–C1411 and G1488-G1497 within the DC cavity (Figure S4c). While hydrogen bonds constituted the most prevalent interaction type across all data sets, the G1491 nucleotide was notably involved in additional interaction types, such as π-interactions. In addition, hydrogen bonding and π-alkyl interactions with residues T39-T41 and N46 of the s12 ribosomal protein were noted. Considering docking scores, nonbonded interactions, and recurrent sequences, we selected a list of peptides for MD simulations. From the DBAASP v3 library, the tyrosidine A analogue (PDB: 6B35), neurokinin A (PDB: 1N6T), the lasso peptide chaxapeptin (PDB: 2N5C), and decoralin-NH2 (PDB: 2N9A); from CyclicPepedia, CP01126 and CP01663; and from CycPeptMPDB, IDs 2508 and 2289 were selected for further MD simulations (Figure 2).

**Figure 2:**
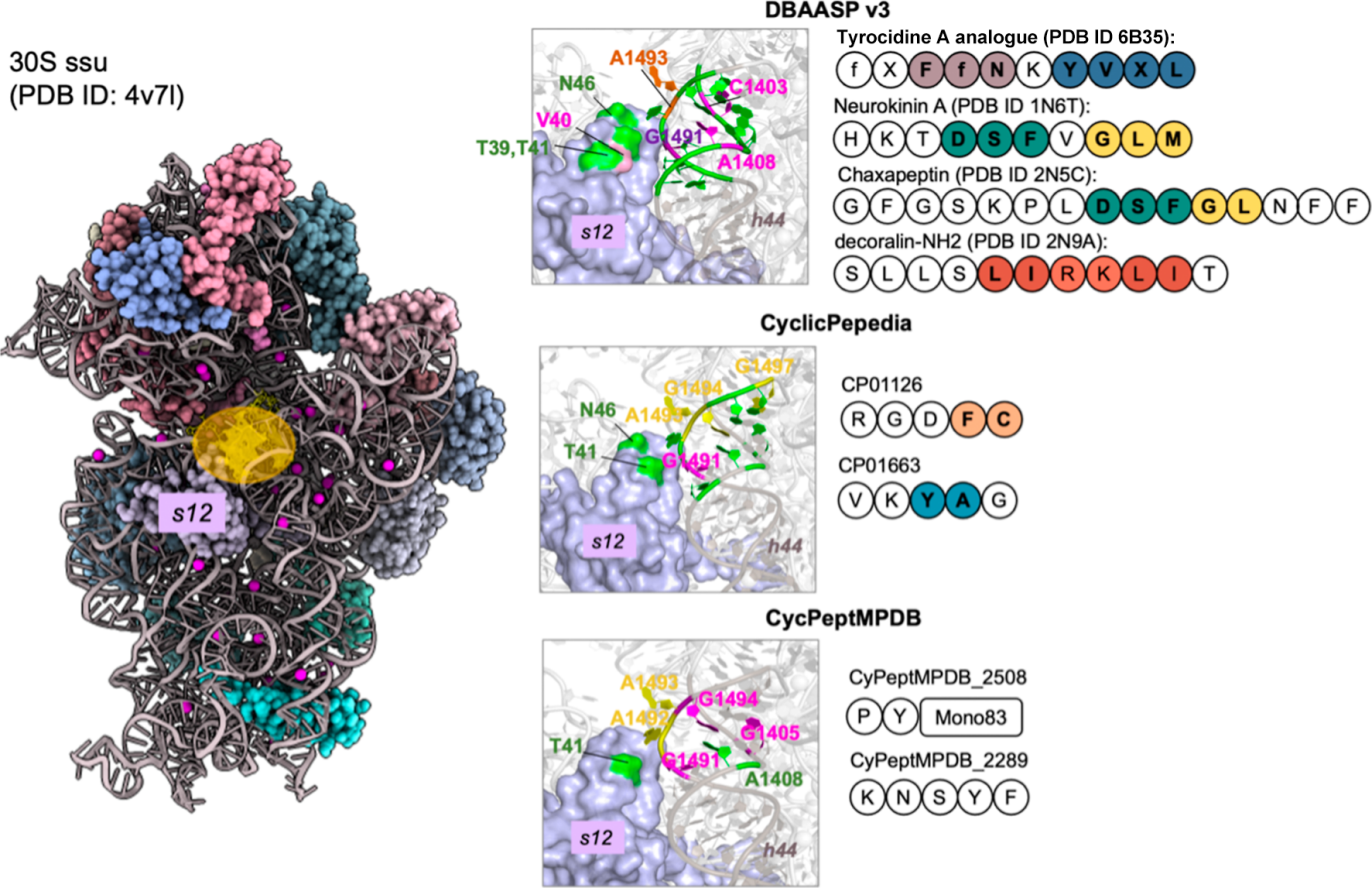
Overview of the peptide selection for the MD simulations and their interaction profile at the decoding center (PDB ID: 4v7l). On the left panel, the 30S is shown with ribosomal protein s12 (purple) and the DC (yellow). On the right panel, the interaction patterns of peptides obtained from the three libraries, based on the consensus docking of Glide and rDock programs, are shown. The recurrent sequences among the selected peptide candidates are shown with distinct colors. Magnesium ions are depicted as magenta spheres. Residues involved in nonbonded interactions are colored according to the interaction type: hydrogen bonds (green), π–cation (orange), π–π interactions (magenta), hydrophobic contacts (slate), and salt bridges (light orange).

#### Peptidyl Transferase Center

In the validation step for the PTC region, cocrystallized peptide inhibitor dalfopristin was redocked to its binding site in the crystal structure (PDB ID: 4u24)^56^ (Figure 1). Dalfopristin redocked poses had docking scores of −12.92 kcal/mol (SP-Peptide in Glide) and −56.11 kcal/mol (rDock), yielding lower RMSD values (1.16 Å for Glide SP-Peptide and 0.82 Å for rDock) compared to the crystal structure (Figure S9). Analysis of the dalfopristin–ribosome 2D interactions within the PTC cavity verified that the peptide maintained its crystal interactions, particularly with nucleotides G2061, A2062, A2503, G2505, and U2506, and π-interactions with nucleotides A2451, C2452, and U2506. The overlap of the ligand conformations in the best pose and the crystal structure, as well as the similarity of the reproduced inhibitor–nucleotide interactions, reconfirmed the parameters of the docking protocol.

The violin plots reveal that peptides from the DBAASP v3 data set exhibited a broad distribution of Glide GScore values in the PTC, with a median score around −9 kcal/mol, indicating a generally favorable binding affinity but with considerable variability (Figure S10a). In contrast, peptides from CyclicPepedia and CycPeptMPDB displayed narrow distributions, with medians around −6 and −7 kcal/mol, respectively, suggesting more consistent but slightly less favorable binding profiles compared to DBAASP v3. Figure S10b shows a similar trend for rDock scores, where DBAASP v3 peptides demonstrate a wide range of docking scores, with a median near −20 kcal/mol, while CyclicPepedia and CycPeptMPDB peptides have more negative median scores (approximately −40 kcal/mol), indicating strong binding affinities.

A total of 52 peptide conformers were filtered with z-scores lower than −1.65 from the DBAASP v3 database in the PTC cavity, with the sequence length of them varying between 6 and 15 amino acids (Figure S3). Based on consensus docking, 17 peptides were obtained (Table S5). Among them, the “IL” and “GF” sequences were frequently observed in hit peptides 536_2 (PDB ID 6RRO),^103^ protonectin (PDB ID 7JHF),^104^ temporin A (PDB ID 2MMA),^105^ BPTI (PDB ID 7M77),^106^ lasso peptide chaxapeptin (PDB ID 2N5C),^95^ and hemagglutinin (PDB ID 2L24).^107^ In addition, 2-mer “WL”, “RK”, and “WW” sequences were noted in designed peptides TetraF2W-RK (PDB ID 6NM2)^102^ and WW295 (PDB ID 6NM3).^108^ Similarly, a total of 143 and 2332 peptide conformers with z ≤ −1.65 were filtered from the CyclicPepedia and CycPeptMPDB databases, respectively (Figure S3). Based on consensus docking, 4 peptides were obtained from the CyclicPepedia database (Table S5) and 8 peptides from the CycPeptMPDB database (Table S6).

The interaction profile plots reveal that peptides from the DBAASP v3 data set form the most diverse and abundant range of noncovalent interactions within the PTC region, including numerous hydrogen bonds and π-interactions (Figure S10c). In contrast, peptides from CyclicPepedia and CycPeptMPDB exhibit fewer and more selective interactions. The interactions of the hit peptides within the PTC cavity are displayed in 2D diagram maps (Figures S11–S14). We selected 5 hit peptides from the DBAASP v3 library, namely, a tyrocidine A analogue (PDB ID: 6B35), hemagglutinin (PDB ID: 2L24) peptide 536_2 (PDB ID: 6RRO), protonectin (PDB ID: 7JHF), and WW291 (PDB ID: 6NM2); 3 peptides from the CyclicPepedia library (CP00937, CP00667, and CP01457); and 4 hit peptides from the CycPeptMPDB library with database IDs 2508, 2476, 2510, and 2289 for MD simulations (Figure 3).

**Figure 3:**
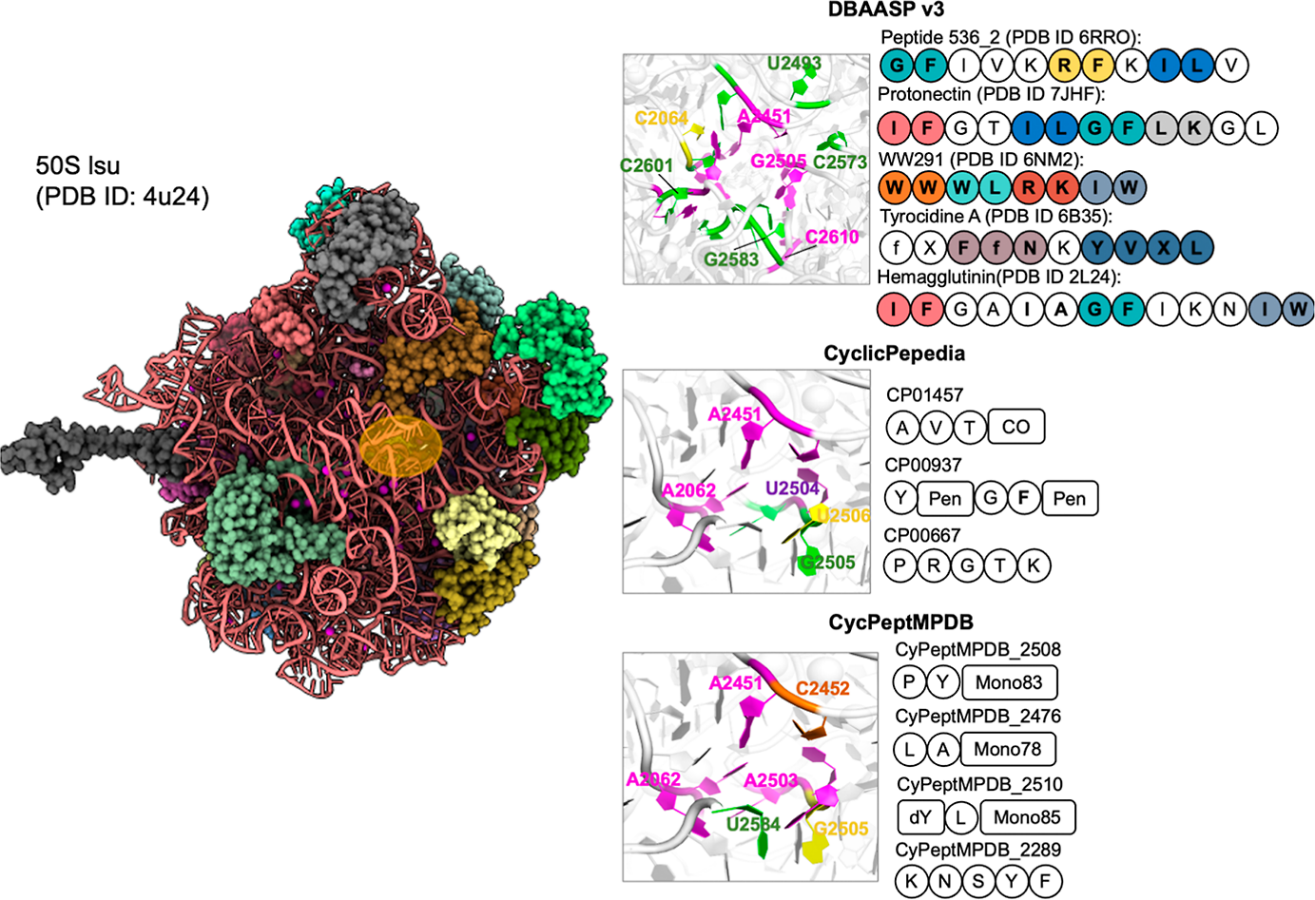
Peptides selected for MD simulations in the PTC cavity. Overview of the peptide selection and their interaction profile on the 50S (PDB ID: 4u24). On the left panel, the 50S is shown, and the PTC pocket is indicated (yellow). On the right panel, the interaction patterns of peptides obtained from the three data sets, based on the consensus docking of Glide and rDock programs within the PTC cavity, are shown. The recurrent sequences among the selected peptide candidates are indicated with distinct colors. Magnesium ions are depicted as magenta spheres. Residues involved in nonbonded interactions are colored according to the interaction type: hydrogen bonds (green), π–cation (orange), π–π interactions (magenta), hydrophobic contacts (slate), and salt bridges (light orange).

#### Putative Binding Pocket

Building on the preceding analysis of results for the orthosteric DC and PTC regions, we next evaluated docking calculations within the putative binding pocket. Since there is no available ligand-bound crystal structure for this pocket, validation of the docking results could not be performed; thus, the docking protocol followed for DC and PTC was applied. Accordingly, the center of the Site 1 pocket identified through SiteMap analysis (*x* = 63.5 Å, *y* = −1.0 Å, and *z* = 69.3 Å) was employed, with an outer grid box size of 25.0 × 25.0 × 25.0 Å^3^ and an inner grid box size of 10.0 × 10.0 × 10.0 Å^3^ for the docking calculations in Glide. For rDock calculations, cavity mapping was performed using the two-sphere method,^36^ utilizing the same center coordinates and a radius of 20 Å.

Glide GScore and rDock docking scores (in kcal/mol) in the DC putative binding pocket showed similar distributions as noted for the active sites (Figure S15a,b). Eight hit peptides were identified using a threshold z-score of −1.65; among these, 3 peptides from the DBAASP v3 database were found to be common to both docking programs (Table S7). Cyclic peptides had a significant affinity for the putative binding pocket, aligned with their enhanced variety of noncovalent interactions. Notably, DC-binding cyclic peptide viomycin also emerged as a candidate for this pocket. It is important to note that recent structural studies (e.g., PDB ID 6LKQ) have identified up to five distinct viomycin binding sites (Vio1–Vio5) on the bacterial ribosome, highlighting its multisite binding nature.^10^ While the docking site at the DC corresponds to the Vio1 site (PDB ID 6LKQ), the proposed putative binding pocket represents a novel site that does not directly overlap with the Vio2–Vio5 sites. The identification of viomycin as a potential ligand for this novel pocket suggests that the structural heterogeneity of viomycin binding may extend beyond the currently characterized sites, offering additional targets for peptide-based modulation. The hit peptides primarily formed hydrogen bonds with the residues in the putative binding pocket. In contrast, peptides from the CyclicPepedia and CycPeptMPDB databases exhibited a broader range of interactions, including hydrogen bonds, π-anion, π-alkyl, salt bridges, and attractive charge interactions (Figure S15c). These findings highlight that while peptide candidates from the DBAASP v3 library relied mainly on polar contacts, hits from the other libraries utilized more diverse binding interactions/poses (Figures S16–S19), potentially enhancing their binding affinity by exploiting their flexibility. We selected 3 hit peptides from the DBAASP v3 library, namely, Arylomycin A2 (PDB ID: 3IIQ), Com5 (PDB ID: 1QVL), and viomycin (PDB ID: 4V7L); 3 peptides from the CyclicPepedia library (CP01953, CP01970, and CP00153); and 3 hit peptides from the CycPeptMPDB library with database IDs 1902, 2508, and 2510 for MD simulations (Figure 4). The recurrent sequence “PG” was frequently observed in hit peptides selected from the CyclicPepedia library.

**Figure 4:**
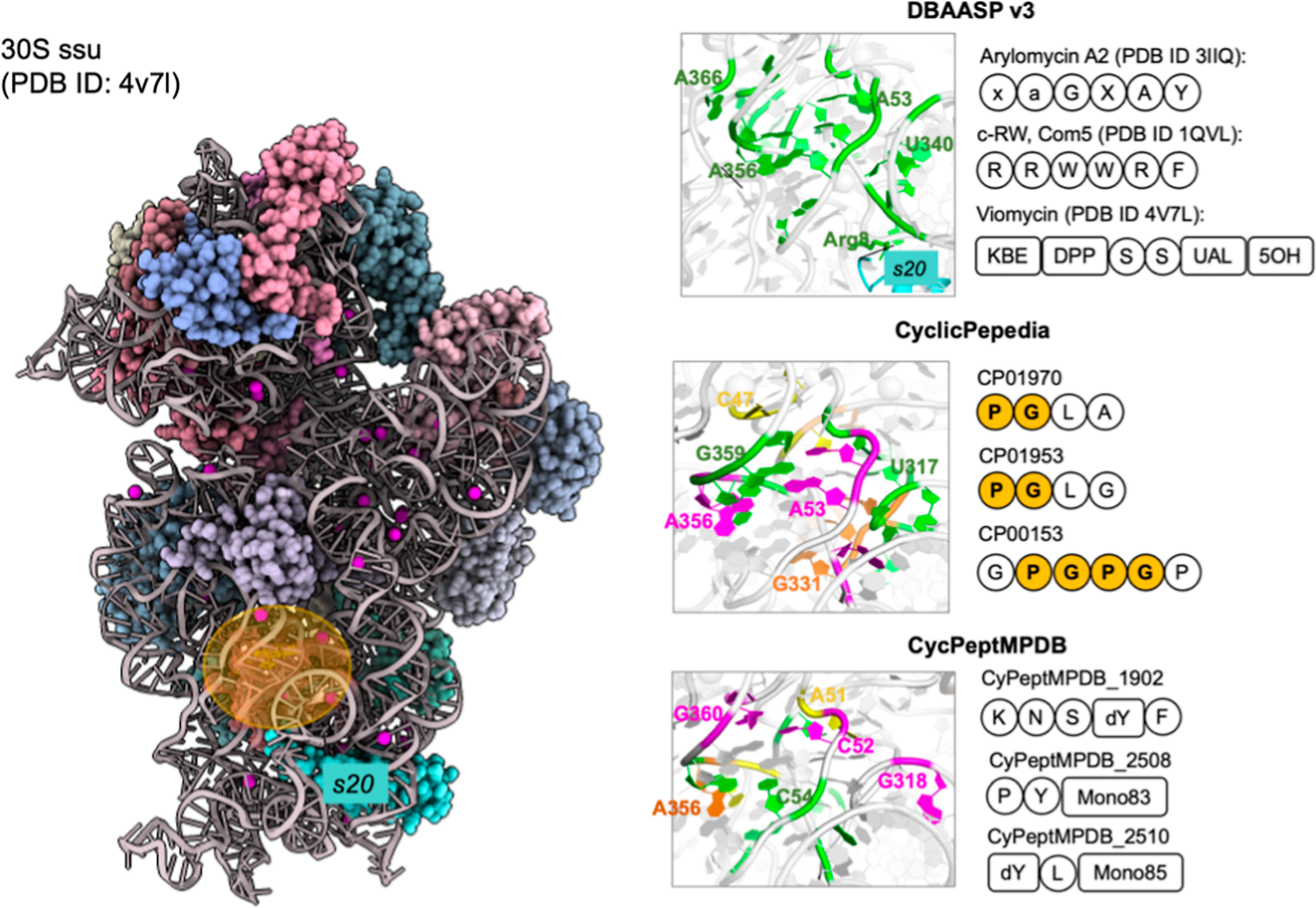
Peptide candidates selected for MD simulations in the putative binding pocket. Overview of the peptide selection and their interaction profile on the 30S ssu (PDB ID: 4v7l). On the left, the 30S ssu is shown with the s20 protein (cyan), and the putative binding pocket is indicated (yellow). On the right, the interaction patterns of peptides obtained from the three data sets, based on the consensus docking of Glide and rDock programs within the binding cavity, are shown. The recurrent sequences among the selected peptide candidates are color-coded. Magnesium ions (Mg^2+^) are depicted as magenta spheres. Residues involved in nonbonded interactions are colored according to the interaction type: hydrogen bonds (green), π–cation (orange), π–π interactions (magenta), hydrophobic contacts (slate), and salt bridges (light orange).

#### B8 Intersubunit Bridge

For the B8 intersubunit bridge region, the center of the grid box was set *x* = 65.1 Å, *y* = −30.9 Å, and *z* = 59.8 Å, respectively. Molecular docking calculations of 23,000 conformers were performed using a grid box with the outer size of 25.0 × 25.0 × 25.0 Å^3^, while the inner grid box size was 10.0 × 10.0 × 10.0 Å^3^. The violin plots of Glide GScore and rDock docking values for the B8 intersubunit bridge region are presented in Figure S20a,b, respectively, where the shapes of the distributions are different for the same libraries, highlighting the difference in the scoring functions. Applying a z-score threshold of −1.65 to the docking results identified 2 consensus peptides from DBAASP v3, 3 from CyclicPepedia, and 11 from CycPeptMPDB (Table S8). Notably, these peptides largely overlap with those identified for the putative binding pocket. The hit peptides from DBAASP v3 primarily formed hydrogen bonds (Figure S21), while those from CyclicPepedia exhibited various π-type and alkyl interactions at the B8 intersubunit bridge (Figures S20c and S22). Peptides from CycPeptMPDB enriched in nonstandard amino acids (e.g., meL, Mono85, Me_dA) display the most diverse interaction landscape, engaging in multiple types of noncovalent interactions such as hydrogen bonds, π-interactions, salt bridges, and attractive charge contacts (Figures S23 and S24). We selected 6 peptides for MD simulations: 2 peptides from the DBAASP v3 library, namely, Arylomycin A2 (PDB ID 3IIQ) and Com5 (PDB ID 1QVL); 2 peptides from the CyclicPepedia library (CP02321 and CP00153); and 2 peptides from the CycPeptMPDB library with database IDs 2508 and 2510 (Figure 5).

**Figure 5:**
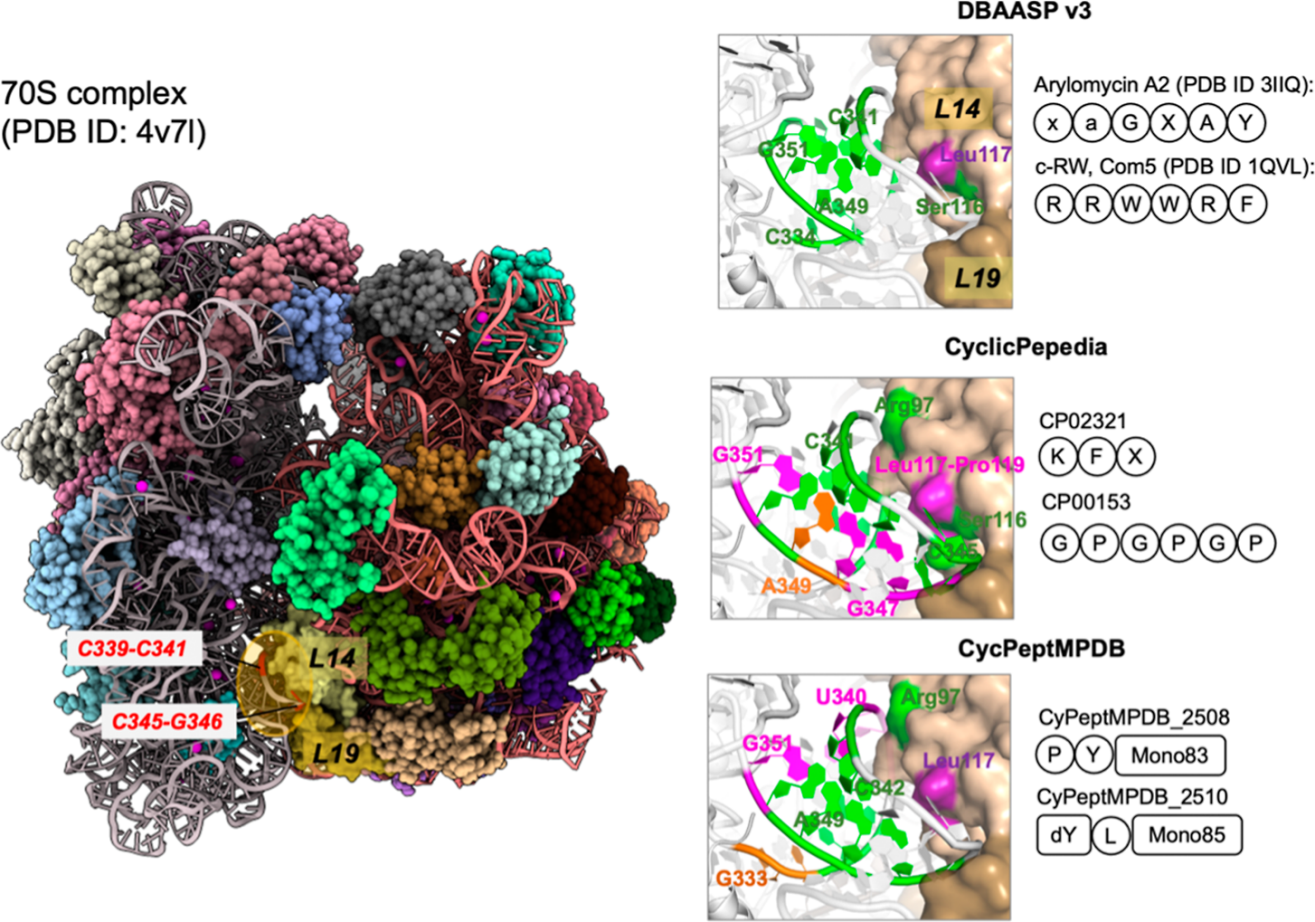
Peptide candidates selected for MD simulations in the B8 bridge. Overview of the peptide selection and their interaction profile on the 70S complex (PDB ID: 4v7l). On the left, the 70S complex is shown, and the B8 bridge is indicated (yellow). On the right, the interaction patterns of peptides obtained from the three data sets, based on the consensus docking of Glide and rDock programs within the binding cavity, are shown. Magnesium ions (Mg^2+^) are depicted as magenta spheres. Residues involved in nonbonded interactions are colored according to the interaction type: hydrogen bonds (green), π-cation (orange), π–π interactions (magenta), hydrophobic contacts (slate), and salt bridges (light orange).

The virtual screening for the B8 bridge was performed using the nonrotated state of the ribosome (PDB ID 4v7l)^55^ while aiming to inhibit translocation by stabilizing the classical conformation. As the B8 bridge undergoes structural remodeling during intersubunit rotation,^109^ these peptides are expected to exhibit state-specific binding, effectively acting as conformational locks.

### Molecular Dynamics Simulations of the Docked Peptide–Ribosome Complexes

#### Decoding Center

The selected hit peptides for the DC region—comprising 4 from DBAASP v3, 2 from CyclicPepedia, and 2 from CycPeptMPDB—and the native ligand viomycin were subjected to three independent 100 ns long explicit MD simulations. These simulations were conducted to evaluate the stability of the peptides within the binding cavity and to assess their interactions with the binding site.

The distribution of RMSD values for the truncated ribosome and the docked peptides to the DC is presented in Figure S25. The RMSD values of the truncated ribosome across all peptide-bound states exhibited fluctuations within the range 0.4–1.2 Å (Figure S25a). Overall, the peptides largely retained their docking poses within the binding site, with the exception of Neurokinin A (PDB ID 1N6T), Decoralin-NH2 (PDB ID 2N9A), and Chaxapeptin (PDB ID 2N5C), which exhibited adjustments in their flexible side chains in one or two replicas (Figure S25b). Then, we analyzed the nonbonded interactions between the docked peptides and the binding site that were present in at least 30% of the MD trajectories (Figure S24c). This analysis was performed using the analyze_trajectory_ppi.py script implemented in Maestro. Notably, the docked peptides retained interactions with one or more nucleotides at the conserved DC anchor (G1491/A1493/G1494) through favorable nonbonded interactions, mirroring the crystallographic contact pattern of viomycin. An interaction fingerprint for each peptide candidate was derived by mapping contact types to the physicochemical class of the contacting peptide residue and to the local nucleotide environment in DC (Figure S26). Two features were most striking of viomycin-like behavior: (i) an aromatic/heteroaromatic moiety forming π–π interactions or backbone amide groups exhibiting hydrogen bond interactions with G1491/A1493/G1494 and (ii) a basic group (Lys/Arg) positioned to engage the backbone or side chain amide group of G1405 via hydrogen bond interactions. In particular, aromatic/heteroaromatic moieties frequently formed π–π stacking or edge–face interactions with G1491, a trend especially pronounced in peptides including the “DSF” sequence (PDB IDs: 2N5C and 1N6T). Peptides expressing both features —most notably neurokinin A (PDB ID: 1N6T) and CycPeptMPDB_2289, followed by chaxapeptin (PDB ID: 2N5C) — displayed interaction fingerprints with high qualitative similarity to viomycin. Nonpolar side chains (Val/Leu/Ile) established persistent hydrogen bond contacts with the G1491-A1493/A1408, reducing peptide mobility within the pocket.

#### Peptidyl Transferase Center

For the PTC region, the native ligand dalfopristin and the selected hit peptides —comprising 5 from DBAASP v3, 3 from CyclicPepedia, and 4 from CycPeptMPDB— were subjected to three independent 100 ns long explicit MD simulations. The distribution of RMSD values for the truncated ribosome and docked peptides is presented in Figure S27. The RMSD values of the truncated ribosome across all peptide-bound states fluctuated within the range 0.3–0.7 Å (Figure S27a). The docked peptides mostly retained their docking poses within the PTC binding site, with minor adjustments observed in the flexible side chains of a few peptides in one or two replicas (Figure S27b) without leaving the binding site. The nonbonded interactions between the docked peptides and the PTC binding site that were present in at least 30% of the MD trajectories are given in Figure S27c. The docked peptides retained interactions with one or more crystallographic contacts of dalfopristin (e.g., G2061, A2062, A2451, C2452, A2503, G2505, and U2506) through favorable nonbonded interactions (hydrogen bonds, π interactions, salt bridges, π–cation contacts, and hydrophobic packing). Dalfopristin maintained its characteristic interactions (Figure S28) involving G2061 (H-bonding), A2451 and C2452 (π interactions), the U2504 and U2506 region (hydrogen bond or hydrophobics), and U2609–C2610 (π-cation). Many peptides recapitulate at least part of this quad, explaining their stable RMSD profile. High-stability peptides typically combine (i) hydrogen bond interaction with G2061 via the backbone carbonyl group or amide nitrogen of nonpolar (Leu) residues, (ii) an aromatic unit stacked against purine bases (often A2062/A2451/A2503), and (iii) hydrogen bonding and/or salt-bridge/π–cation interactions with nucleotides U2504–U2506, mediated by Lys/Arg or N-methylated amide nitrogens. Macrocyclic peptides (e.g., tyrocidine A, 2476, 2508, 2510) presented aromatic surfaces that favor π–π or edge–face interactions with A2062/A2451 while maintaining hydrogen bond interactions with G2505. Peptide candidates with positively charged residues (Lys/Arg) (e.g., peptide 536_2, CP00667, and 2289) frequently formed persistent hydrogen bonds or π–cation contacts with C2063/U2585/U2586/U2609 and neighboring phosphates, potentially enhancing residence within the core of the PTC. Overall, peptides that reproduced dalfopristin’s electrostatic–aromatic dual anchoring and contacted the G2061, A2451, and U2504–U2506 hot spots displayed the most robust interaction fingerprints, suggesting them as prioritized scaffolds for further experimental validation.

#### Putative Binding Pocket

For the putative binding pocket, the selected hit peptides —comprising 3 from DBAASP v3, 3 from CyclicPepedia, and 3 from CycPeptMPDB— were investigated with the MD simulations. The RMSD analyses revealed that the truncated ribosome maintained a high structural stability across all simulations (Figure S29a). Ligand RMSDs were generally lower than those observed at the active sites (Figure S29b), indicating a stable engagement within the confined binding pocket. Interaction fingerprinting further supported these observations (Figure S29c), where recurrent contacts with nucleotides on the 16S rRNA included frequent hydrogen bond, π–π, and salt-bridge interactions. Notably, nucleotides such as C47, G113–U114, and A353–A356 appeared as recurrent hotspots across chemically diverse peptides and replicas, implicating them as key stabilizing elements of the putative binding site. Residues with aromatic side groups frequently stacked with adenine/cytosine bases via π–π or hydrogen bonds, while positively charged residues formed salt bridge contacts, and polar residues exhibited hydrogen bond interactions with guanine/uracil bases in the binding cavity (Figure S30). Polar residues (cyan) appear less often as primary anchors but assist in the stabilization of viomycin and CycPeptMPDB_1902 peptides via hydrogen bond interactions. Peptides containing the special residues (Pro/Gly) exhibited hydrogen bond interactions with A353 and G357, pronounced among CyclicPepedia hits bearing the recurrent “PG” sequence. Accordingly, stable binding in the putative pocket was achieved through a hybrid binding mode: specific hydrogen bonding or salt bridge contacts with the conserved nucleotide triad (C47–A353–A356/C355) coupled with aromatic stacking involving C47 and/or A353. Taken together, these interaction patterns underlie unique binding modes for cyclic peptides and may provide a basis for advancing candidates with enhanced ribosome affinity to experimental validation.

#### B8 Intersubunit Bridge

The B8 intersubunit bridge encompasses the 30S and 50S subunit interface. Here, ribosomal proteins L14 and L19 of 50S contact the loop (nucleotides 345–346) and stem (nucleotides 339–341) regions of helix h14 in the 16S rRNA on 30S, predominantly via electrostatic interactions with the phosphate backbone.^61^ Mutations at B8 have been linked to miscoding, leading to defects in both initial selection and proofreading,^110,111^ and are proposed to act by aberrantly triggering GTP hydrolysis.^112,113^ We therefore focused on the B8 intersubunit bridge as a mechanistically tractable node where electrostatic L14/L19-rRNA contacts couple intersubunit communication to decoding fidelity, making it a promising site for peptides that modulate ribosome function.

MD simulations of the selected peptides bound at the B8 intersubunit bridge showed that the truncated ribosome remained structurally stable across all replicas, with low RMSD fluctuations (Figure S31a). By contrast, the ligand RMSDs displayed broader variability in the conformational dynamics at the subunit interface (Figure S31b). Peptides generally retained their initial docking poses, except peptide CycPeptMPDB_2510 diffused away from the binding site around 200 ns in replica 3. Interaction fingerprinting further revealed that peptide stabilization in the binding site involved both large-subunit proteins L14 and L19 and neighboring 16S rRNA nucleotides (Figure S31c). For example, the R97 residue on the L14 protein engaged in hydrogen bonds with peptide backbone carbonyls —including aromatic rings (2508) and special-case residues (CP00153)— and was involved in π–cation interactions via aromatic rings in CP02321. In addition, h14 nucleotides C339 and C341 established hydrogen bonds with aromatic or special-case groups in Com5, Arylomycin A2, and CP00153 (Figure S32). Besides these residues, the stabilization of the selected peptides at the B8 intersubunit bridge was mostly maintained by the Y68 residue of the L19 protein, the S116 residue of L14, and C337 and G347 on h14, forming hydrogen bonding, π-interactions, salt bridges, and hydrophobic interactions across multiple peptides and replicas, highlighting their potential role as stabilizing hotspots. Notably, crystal structures of mutant 70S ribosomes indicate that the G347U point mutation induces a distortion of helices h8/h14, thereby disrupting the B8 intersubunit bridge.^114,115^ Y68 frequently formed π–π interactions with aromatic rings of the peptides and also participated in π–cation contacts via backbone/side chain amide nitrogens. S116 formed hydrogen bonds with either aromatic moieties or amide nitrogens. C337 and G347 formed hydrogen bonds with polar donors (NH or OH) and, in some cases, salt-bridged contacts with positively charged amino acids. In summary, these results prioritize cyclic scaffolds that present (i) an aromatic unit oriented for stacking with Y68, (ii) H-bond donors/acceptors positioned to engage S116 or C337/G347, and (iii) a cationic handle capable of π–cation or salt-bridge formation. Indeed, proteins containing arginine- and glycine-rich recurrent sequences—such as the RGG—have been identified as functionally involved in the regulation of translation in cells.^116^ Enhancing redundancy around these hotspots may improve the residence time and reduce the risk of dissociation, as suggested by the behavior of peptide 2510 in one replica.

### Investigating the Intrinsic Dynamics and Structural Flexibility of the *E. coli* 70S Ribosome

To assess whether perturbations at the putative binding pocket and the B8 intersubunit bridge may propagate to influence the global dynamics and long-range communication mechanisms of the 70S ribosome, we performed four independent 750 ns long coarse-grained MD (CGMD) simulations and analyzed the intrinsic dynamics and structural flexibility of the 70S complex around its native conformational state. The intrinsic dynamics of the ribosome complex are dictated by its structural flexibility, inherent in its contact topology. These vibrational motions usually correspond to collective functional motions of the structure at the low-frequency end of the spectrum and give useful insights into the pathways through which allosteric signals can propagate.^79^ In this study, CGMD simulations were employed to sample the equilibrium fluctuations that reveal the underlying correlation networks and allosteric infrastructure embedded in the ribosome’s contact topology at a specific functional state. Here, we assumed that a local perturbation due to ligand binding is transmitted via residue-level fluctuations consistent with an allosteric mechanism, as investigated in our previous studies.^80^ Since explicit atomistic interactions between peptides and the ribosome cannot be captured in the CG simulations, we studied the global dynamics of the apo 70S ribosome structure. We focused on the peptide-binding nucleotides/residues identified from the explicit MD simulations and investigated their cross-correlations. In this line, we mapped the intrinsic ribosomal dynamics to reveal how these binding sites are embedded within the native correlation network and its long-range communication pathways supported by energetically inexpensive collective motions.

To measure the conformational stability and flexibility during the simulations, we monitored the RMSD and root-mean-square fluctuations (RMSF), respectively. The system reached local structural equilibration within approximately 150 ns, which was excluded from subsequent analyses (Figure S33a). All analyses were then conducted on the concatenated production trajectories from four replicas of the 70S complex. The RMSD values increased to ∼4.0 Å, largely reflecting pronounced motions of the uL1 stalk (Figure S33b). Since the 4V5H crystal structure lacks experimental *B*-factors, these values were obtained from a high-resolution (3.0 Å) crystal structure with PDB ID 4V9D.^117^ To enable a direct comparison, theoretical *B*-factors were computed from the CGMD-derived RMSF values using $B=\frac{8}{3}\pi^{2}{RMSF}^{2}$ ; both experimental and theoretical *B*-factors were then normalized, and the Pearson product–moment correlation coefficient was calculated, following the procedure described in the RedMD framework.^77^ The experimental and theoretical fluctuation profiles were in good agreement with Pearson product-moment correlation coefficients of 0.74 for 16S rRNA (Figure S33c) and 0.75 for 23S rRNA (Figure S33d). Subsequently, global motions of the *E. coli* 70S ribosome were characterized by principal component analysis (PCA) using Bio3D,^82^ following alignment to the 50S core atoms (excluding the 30S ssu and the uL1 and uL11 stalks). The scree plot shows that the first 20 principal components account for over 30% of the total variance (Figure S34a). PC1 captures the ratchet-like motion between the 30S and 50S subunits^118^ (Figure S34b)—a motion essential for tRNA translocation and driven with or without EF-G-mediated GTP hydrolysis and occurring on slower time scales (seconds) as reported by Cornish et al. (2008)^119^ yet intrinsically sampled during our microsecond-scale simulations. PC2 and PC3 depict the opening/closing dynamics of the subunit interface (Figure S34c,d). Investigation of the 30S subunit suggests motions reminiscent of head and spur movements and body closure toward the 50S large subunit (Figure S34b–d), in agreement with previous observations.^120^

We extended the analysis by computing a dynamic cross-correlation matrix (DCCM) from the first 10 principal components, using concatenated CGMD trajectories aligned to the 50S core atoms after excluding the L1 stalk. The intrinsic dynamics of the *E. coli* 70S ribosome via DCCM were characterized to infer how peptide engagement at the putative binding pocket and the B8 intersubunit bridge would propagate through the complex: where effects originate, how they travel, and how far they extend. This framework has the capability to provide a good reference for predicting the axes along which allosteric effects are likely to propagate upon inhibitor binding. We selected nucleotides C47, A353, and A356 that are identified by the interaction fingerprint at the putative pocket as seed nodes and computed their correlation/anticorrelation profiles with distal regions of the 30S and 50S subunits. To emphasize meaningful couplings and minimize spurious signals, we applied a threshold of |*C*_*ij*_| > 0.4.

The putative binding pocket exhibits positively correlated motions with multiple elements of the small subunit, including the spur, shoulder, beak, helix h44, and h45 of 16S rRNA, as well as ribosomal proteins s2, s3, s4, s5, s8, s10, s12, s14, s16, s17, s19, and s20 and with the large-subunit 23S rRNA nucleotides (Figure 6a). Notably, pocket nucleotides also had correlated motions with residues in proteins L14 and L19, which together participate in the formation of the intersubunit bridge B8.

**Figure 6:**
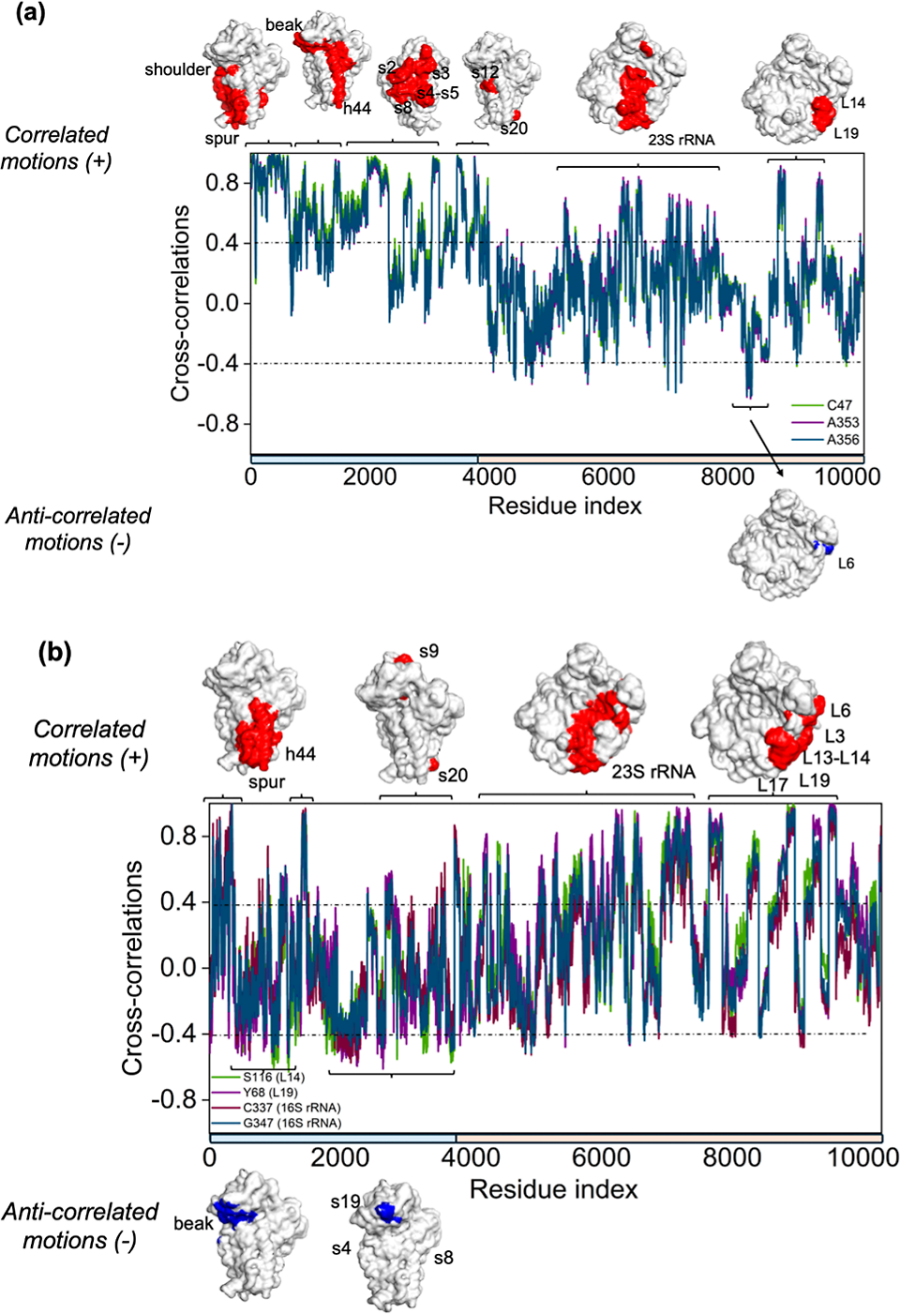
Cross-correlations of the (a) putative binding-pocket nucleotides C47, A353, and A356 and (b) B8 bridge residues/nucleotides S116 (L14), Y68 (L19), C337, and G347 across the 70S ribosome. The central plots in (a) and (b) show residue-wise cross-correlations; dashed lines mark thresholds at *C*_*ij*_ = +0.4 and −0.4. Structural insets highlight subunit regions whose motions are strongly correlated (|*C*_*ij*_| ≥ 0.4): positively correlated regions are colored red, and anticorrelated regions are colored blue on the 30S small and 50S large subunits. Labels indicate rRNA helices/proteins corresponding to selected peaks.

Extending this approach to the intersubunit bridge B8, we analyzed in detail the cross-correlation profiles of residues S116 (L14), Y68 (L19), and the 16S rRNA nucleotides C337 and G347 to identify the subunit regions whose motions are strongly coupled to the B8 interface. The B8 bridge interface shows positively correlated motions with the 16S rRNA spur and helix h44, with small-subunit proteins s9 and s20 and with large-subunit proteins L3, L6, L13, L14, L17, and L19 (Figure 6b). Additionally, we observed negatively correlated motions with ribosomal protein s4 (*C*_*ij*_ = −0.47). Prior genetic studies showed that mutations in the B8 component L19 alleviate phenotypes caused by error-restrictive mutations in s12,^121^ and, consistent with this, Sun et al. (2011) demonstrated that substitutions in 16S rRNA nucleotides forming intersubunit bridge B8 likewise suppress some of the decoding defects associated with s12 mutations.^112^ Fagan et al. (2013) reported that the 16S rRNA ram mutation G299A, located in helix h12 near the s4–s5 interface, is allosterically linked to the B8 intersubunit bridge, induces the largest structural rearrangements in h8/h14 at B8, and facilitates transition to the GTPase-activated state.^114^ Numerous well-characterized mutations in these proteins compromise translational fidelity, suggesting that perturbations in s4 and s5 may modulate decoding by transmitting their effects via the B8 intersubunit bridge.^122,123^ Here, we observed that the B8 intersubunit bridge displays correlated motions with the putative allosteric pocket nucleotides, which themselves also correlate with proteins s4 and s5 (Figure 6a). Intrinsic dynamics and cross-correlations of the *E. coli* 70S ribosome suggest that ligand binding at the B8 intersubunit region has a high propensity to alter the dynamics of the distant functional regions and decoding fidelity of the bacterial ribosome.

### A Potential Allosteric Mechanism due to a Perturbation at the B8 Intersubunit Bridge Region

Many mutational and high-resolution structural studies have shown that distant regions of the ribosome communicate through coordinated conformational changes and long-range tertiary interactions.^124–126^ While these experimental studies delineate the sites engaged in allosteric communication, the mechanistic pathways by which a perturbation at one site is conveyed to another are not yet fully understood. Previously, we determined putative allosteric pathways in the ribosome using k-shortest paths^127^ and a contact-topology-based residue interaction network (RIN) analysis,^52^ and on this basis, we proposed new druggable sites. Here, we investigated potential allosteric communication pathways using a RIN constructed from coupled dynamics derived from the first ten PCs across four independent CGMD simulations. The aim was to identify hub residues/nucleotides near the putative binding pocket and the B8 intersubunit bridge and to assess their potential roles in mediating allosteric signal transmission. Hub residues with betweenness centrality in the top 5% from the RIN were selected to identify residue pairs exhibiting strong correlations—either positive or negative—relative to other pairs (Figure 7a). The complete list of hub residues and nucleotides is given in Tables S9 and S10.

**Figure 7:**
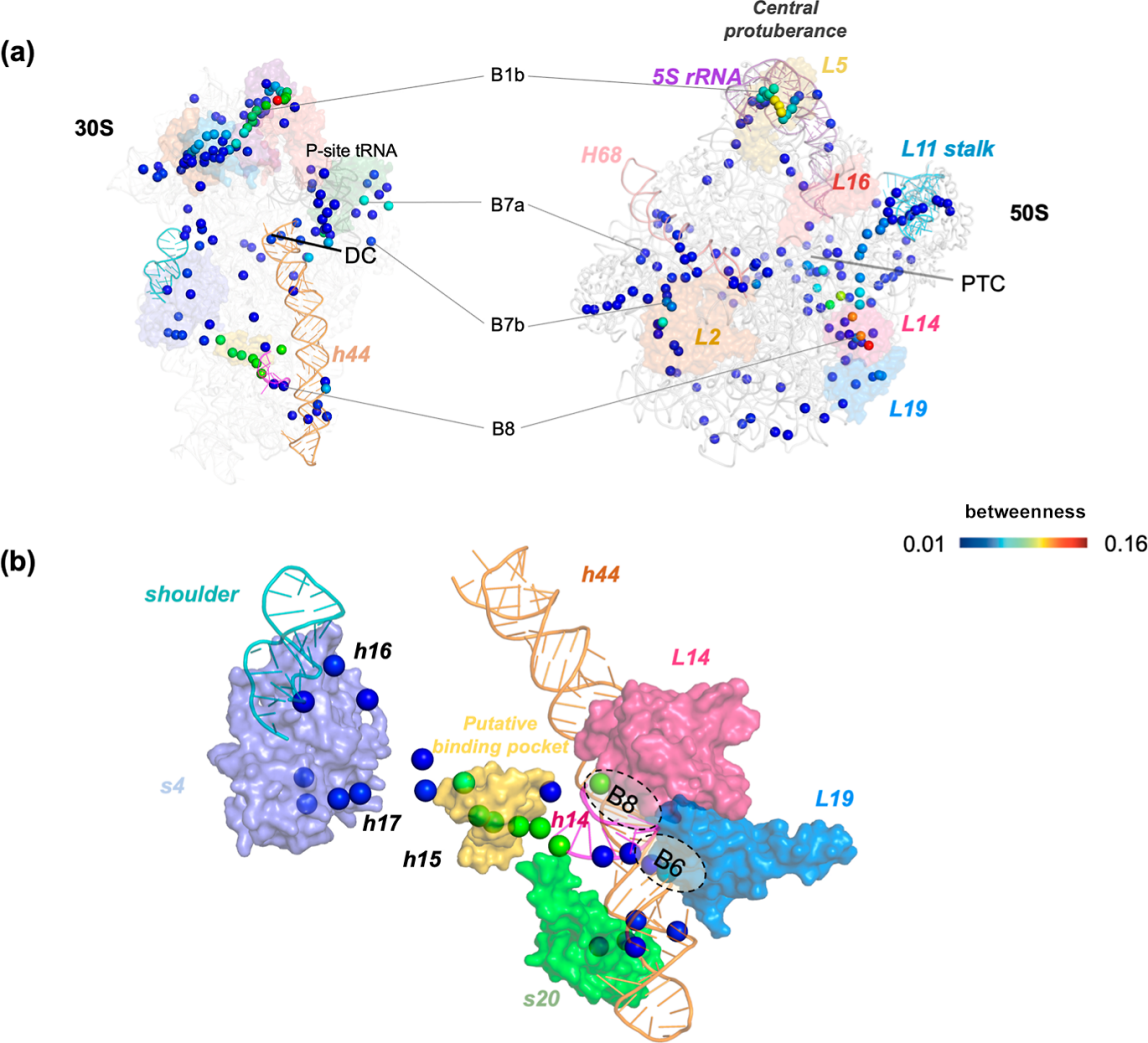
(a) Hub residues from a residue interaction network constructed using cross-correlation values using the top 10 PCs from four independent CGMD simulations of the *E. coli* 70S ribosome (PDB ID: 4V5H) are shown as spheres and color-coded according to their betweenness values. (b) Hubs located between the B8 intersubunit bridge and the shoulder highlight the potential communication pathways.

The hub residues revealed key interaction hotspots within the 70S ribosomal complex. These residues are poised to mediate long-range communication and contribute to allosteric regulation of ribosomal dynamics. Analysis of hub residue distribution indicated that multiple intersubunit bridges host highly connected residues, consistent with a modular network in which the 30S and 50S subunits function as separate dynamic entities.^52^ Residues in the top 0.05 quantile of the betweenness centrality distribution are located on the flexible intersubunit bridges B1b, B7a, B7b, and B8 (Figure 7a), suggesting their roles in mediating intersubunit communication via coupled dynamics. Bridge B1b contains hub residues E58 in s13 and G115 in L5, which is the neighbor of the B1b residue R114.^61^ The flexibility of this bridge is thought to facilitate the swivel motion of the 30S head that drives tRNA translocation.^117^ Bridge B7a emerges as another hub region, where contacts are established between G698 on h23 (the neighbor of the reported B7a residue A702)^61^ and G1846 on H68. These interactions are essential for the structural integrity of intersubunit bridge B7a, which aligns with experimental observations that the truncation of H68 disrupts this bridge and leads to a decrease in overall translation activity.^128^ Nearby, we identified bridge B7b as a hub region that links G774 on h23 to residue L201 of L2 in the large subunit, adjacent to the reported B7b residue M200.^61^ Owing to their location adjacent to the E-site tRNA, bridges B7a and B7b are strategically positioned to facilitate rapid communication of conformational changes across subunits. Notably, E-site tRNA, interacting with s7 and s11 of the 30S as well as the L1 stalk and helix H68 of the 50S, has been identified as an essential contributor to translational accuracy.^129^ Furthermore, hub residues were detected in the B8 bridge, linking C335 and C334 on h14 on the small subunit (adjacent to the reported B8 bridge residues C337 and C345) to L14 in the large subunit. Another group of highly coupled residues links the B8 bridge to the base of the L11 stalk, where the elongation factors bind. By structurally linking conserved rRNA and protein components, bridge B8 acts as a dynamic checkpoint that regulates GTPase activation of EF-Tu to ensure translation fidelity.^61^ In addition, 16S rRNA nucleotides (e.g., h14, h15, h18, h21, h37, h41, and h44) and associated small subunit proteins (e.g., s2, s3, s4, s5, s7, s8, s9, s10, s16, and s20) harboring hub residues are annotated to highlight their putative roles in mediating allosteric communication between distal regions of the ribosome.

Two allosteric communication pathways were identified in the 30S body (Figure 7b). First, the B8 intersubunit bridge and the shoulder on 30S are coupled in their dynamics, with a communication pathway traversing the putative binding pocket through the correlated fluctuations of nucleotides/residues on h14 → h15 → s4 → h16 (shoulder). This long-range pathway suggests that perturbations originating in the B8 intersubunit bridge may propagate to the putative binding pocket and shoulder (helix h16) via correlated, coupled motions of these hub residues and their close neighbors. Such signal transmission has a high capacity to modulate domain-closure movements, particularly during tRNA accommodation and mRNA decoding. In agreement with this finding, ribosomal ambiguity mutations at G299A near the s4–s5 interface and G347U at the intersubunit bridge B8 cause miscoding *in vivo*, most probably due to the GTPase activity of EF-Tu binding at this interface.^114^ This is also consistent with the extended role proposed for B8 through coarse-grained structure-based (SMOG) models, where it acts as a central coordinator for subunit rotation kinetics while influencing tRNA selection fidelity.^130^ The observed coupling between the B8 intersubunit bridge and the shoulder thus points to a plausible mechanism by which the ribosome coordinates local structural perturbations with global conformational rearrangements, highlighting this site as an attractive allosteric binding site. A second pathway was also noted around the B6 bridge at the helix h44, highlighting coupled motions of h44 nucleotides G1435, A1437, G1454, U1460, and A1461 and s20 residues R22 and K31. Given that these nucleotides on h44 neighbor the B6 intersubunit bridge^61^ and the B8 intersubunit bridge, this pathway implies that perturbations at the putative allosteric site can be relayed to the B6 intersubunit bridge through coordinated dynamics.

The conservation analysis indicated that the hub nucleotides connecting B8 and the shoulder (Figure 7b) are highly conserved (>98%) (Figure S35). The overlap of high betweenness centrality and strong evolutionary conservation suggests that these nucleotides/residues have a high potential to play a role in long-range allosteric signaling at this conformational state.

To further understand the role of the hub residues in the flow of information from the B8 intersubunit bridge to the 30S shoulder, their cross-correlations in the network were weakened to obtain a perturbed network for each hub residue, as shown in Figure S35. If the B8 intersubunit bridge and the shoulder are allosterically communicating through coupled motions of the residues, there should be at least one pathway, the shortest pathway. In line with this, the shortest path between A344 (B8 intersubunit bridge) and the base of the shoulder (U409) was calculated for the unperturbed and perturbed RINs. Table S11 indicates that the allosteric coupling values decreased after perturbation of the hub residues shown in Figure S35a. It should be noted that the investigated RIN consists of over 10,000 nodes, and the calculated efficiencies seem to be small. Nevertheless, U367 (h15) at the putative allosteric site showed the most notable decrease in allosteric coupling, identifying it as a major functional bottleneck in the B8 → shoulder communication. Notably, residues with higher betweenness centrality generally corresponded to the highest drops in pathway efficiency upon perturbation.

The effect of a perturbation at these hub residues on the global efficiency of the network was calculated and is presented in Table S11. Perturbations caused minimal changes in global efficiency (Table S11), indicating that while specific communication pathways (e.g., between the B8 bridge and shoulder) are disrupted, the overall ribosome network remains robust and functionally intact. Here, the residues causing the largest drops in pathway efficiency, such as G351 (h14), A356 (h14), and U367 (h15), also showed the most notable impact on global communication efficiency, confirming their role as essential pillars for both specific signaling and overall network communication. In addition, the interconnectedness of the hub residues was measured by their local efficiencies, E_loc_, which shows the efficiency of communication among their neighbors when the hub is removed. High E_loc_ values, such as for K120 (s4) and C440 (h17) indicated robust local communication. Residues with lower local efficiency values, such as G355 (h14) and A356 (h14), were confirmed as critical local bridges whose perturbation could disrupt communication among their neighbors.

Collectively, our findings underscore the value of integrating RIN analysis with CGMD simulations to elucidate allosteric communication pathways linking functionally significant regions in large macromolecular assemblies such as the ribosome in this functional state.

## Conclusion

We presented a coherent, end-to-end strategy for advancing peptide modulators of the bacterial ribosome from discovery of *in silico* toward experimental tractability. Rather than relying solely on docking scores, we linked site mapping, consensus docking of Glide and rDock, and explicit-solvent dynamics to interaction fingerprints that explain why specific scaffolds persist in distinct ribosomal environments. In parallel, coarse-grained dynamics and coupled motions due to the native ribosome dynamics situate these binding events within the native communication architecture of the 70S complex, highlighting residues and regions that are poised to transmit local perturbations into functionally relevant motions. More broadly, the workflow that is augmented here by the first application of the viparr module to truncated ribosome–peptide complexes, which ensures accurate force field parametrization for the RNA-protein system, demonstrates how RNA docking and multiscale dynamics can be integrated to filter peptide candidates before synthesis.

Three outcomes emerge with direct implications for the design. First, recurrent electrostatic–aromatic anchoring patterns—stacking against purines coupled with basic handles and targeted hydrogen bonds—offer portable rules for enhancing peptide residence and selectivity in RNA pockets and/or RNA-protein interfaces. Second, the shared contact chemistry across the active sites, the putative binding pocket, and the B8 intersubunit bridge supports a modular view of peptide–ribosome recognition, possibly enabling reuse of consensus scaffolds, specifically those combining aromatic stacking with cationic or hydrogen-bonding groups, across multiple pockets. CycPeptMPDB_2508 (P-Y-Mono83) exemplifies this outcome by binding across all investigated sites and, thereby, providing a plausible starting core for peptide optimization. In line with previous reports on substitution-driven activity modulation in natural antimicrobial peptides such as ARV-1502 ^131^ and the design of branched peptides with multivalent interaction,^132^ conjugates of aminoglycosides with peptides,^133^ and peptide nucleic acids (PNAs),^134^ our findings suggest that rational modifications based on peptide–RNA-binding cavity interactions may enable the design of more potent peptide-based therapeutics^135^ to combat the global antibiotic resistance crisis. Third, the mapping of dynamic allosteric communication pathways near the putative binding pocket and B8 intersubunit bridge suggests leverage points for tuning decoding fidelity via allosteric intervention.

We propose the following next steps: (i) validate prioritized peptides biophysically and functionally, (ii) test predicted allosteric couplings with targeted mutations at network hubs, and (iii) enhance design using the interaction fingerprints identified here. By closing the loop between modeling and experiment, this framework can accelerate the emergence of peptide therapeutics that exploit both orthosteric and allosteric control of the bacterial ribosome.

## Supplementary Material



## Data Availability

The authors confirm that the data supporting the findings of this study are available within the article and its Supporting Information. The codes used in Residue Interaction Network (RIN) analysis are available at https://github.com/kurkcuoglulevitaslab/Residue-Interaction-Network-Analysis.

## Notes

Theoretical section for computational details; details of the putative binding pocket; details of the MD input structures; results of the molecular docking calculations; list of hub residues; efficiency analysis; docking sites around B8; validation results for the docking calculations; workflow; comparative analysis of peptide data sets for the target regions; 2D interaction maps for docking calculations; results for the all-atom MD simulations; interaction fingerprints; analysis for the CGMD simulations; PCA results; and analysis of the effect of hub residues on network efficiency (PDF)
