# Supplementary material for: Multiscale Modeling of the Bacterial Ribosome to Identify Potential Peptide Modulators and Their Allosteric Effects
Source: Biochemistry. 2026 Apr 27;65(13):2089–107. doi: 10.1021/acs.biochem.5c00832 (PMC13348021; doi:10.1021/acs.biochem.5c00832)
Supplement: Supplementary file 1 [file bi5c00832_si_001.pdf]

Supporting Information

Multiscale Modeling of the Bacterial Ribosome to Identify Potential Peptide Modulators and their Allosteric Effects

Merve Yuce[a], Fethiye Aylin Sungur[b*], and Ozge Kurkcuoglu[a*]

[a]Department of Chemical Engineering, Istanbul Technical University, Istanbul, 34469, Turkey

[b]Computational Science and Engineering Division, Informatics Institute, Istanbul Technical University, Istanbul, 34469, Turkey

*Email: aylin.sungur@itu.edu.tr, olevitas@itu.edu.tr

Table of contents

Computational methods **S2**

Supplementary tables **S4**

Supplementary figures **S14**

Computational methods

Binding pocket detection using Sitemap

The SiteScore for a given pocket is calculated as follows:

$$SiteScore = 0.0733\sqrt{n} + 0.6688e - 0.20p \quad (1)$$

where n is the number of site points (capped at 100), e is the enclosure score, and p is the hydrophilic score (capped at 1.0 to limit the influence of hydrophilicity in highly charged or polar sites). According to SiteMap's knowledge base, a SiteScore greater than 1.0 indicates a druggable site, a score between 0.8 and 1.0 suggests a region that is challenging for binding, and a score below 0.8 denotes a non-druggable site.[1] DScore uses the same properties as SiteScore but applies different coefficients, as shown below:

$$DScore = 0.094\sqrt{n} + 0.60e - 0.324p \quad (2)$$

Molecular Docking to the Peptidyl Transferase Center of the *E. coli* ribosome:

Setting the parameters of Glide / Validation of the Docking Protocol: In order to obtain a reliable protocol for docking with Glide SP-Peptide, the x-ray poses of viomycin were reproduced by setting the parameters as described in the **Methods**. In SP-docking mode, the minimized poses obtained during the docking were ranked using the empirical GlideScore (GScore) function with the following equation,[2]

$$SP\ GlideScore = C_{lipo-lipo}\sum f(r_{lr}) + C_{hbond-neut-neut}\sum g(\Delta r)h(\Delta\alpha) + C_{hbond-neut-charged}\sum g(\Delta r)h(\Delta\alpha) + C_{hbond-charged-charged}\sum g(\Delta r)h(\Delta\alpha) + C_{max-metal-ion}\sum f(r_{lm}) + C_{rotb}H_{rotb} + C_{polar-phob}V_{polar-phob} + C_{coul}E_{coul} + C_{vdW}E_{vdW} + solvation\ terms \quad (3)$$

where C values represent the coefficients, namely the lipophilic-lipophilic, hydrogen bonding (neutral-neutral, neutral-charged, and charged-charged donor and acceptor), metal-ion interactions, rotatable bonds, penalty for polar and hydrophobic parts, Coulomb, van der Waals, and solvation terms. The SP-Peptide mode of Glide is specifically optimized for parameters such as funnel width, number of directions, diameter rotational angle, grid density, van der Waals scaling, rigid docking, and the inclusion of crystallographic conformers in order to predict the binding affinity and binding poses of polypeptides more accurately.

During the validation step, the docked poses are compared to the native structures (PDB IDs: 4v7l, 4u24), and the parameters of the calculations that yield a low RMSD were noted. With this purpose, viomycin and dalfopristin were extracted from the complex ribosome structure, prepared with the Protein Preparation Wizard implemented in Maestro, and re-docked to the binding site by changing/adjusting the default values of the parameters in the software. The 30S small subunit structure (16S rRNA, ribosomal proteins, crystal waters, Mg^{2+} ions) in the protein preparation module (Schrodinger Release 2024-3) had a net charge of -936, and viomycin was protonated with a charge of +3. The 50S large subunit structure (23S rRNA, 5S rRNA, ribosomal proteins, crystal waters, Mg^{2+} ions) in the protein preparation module (Schrodinger Release 2024-3) had a net charge of -2312, and dalfopristin was protonated with a charge of +1.

To evaluate the robustness of the docking protocol in addition to redocking, cross-docking calculations were performed using capreomycin, a tuberactinomycin antibiotic that binds to the decoding center (DC) as viomycin. It was docked into both its native structure (PDB ID 4v7m, small subunit) and the viomycin-bound structure (PDB ID 4v7l, small subunit). Docking studies were performed using Maestro-Glide at standard precision peptide (SP-Peptide) mode. The results demonstrated that the docking pose maintained the experimental binding mode with an RMSD of 1.55 Å and a Glide docking score of -7.48 kcal/mol in the native structure (PDB ID 4v7m). The cross-docking into the alternative structure yielded an RMSD of 1.60 Å and a Glide docking score of -7.39 kcal/mol. In both cases, the cyclic core of

capreomycin aligned well with the crystallographic pose. These results showed that the docking protocol was capable of reproducing experimentally observed binding modes across different ribosomal structures.

Setting the Parameters of rDock. rDock employs a combination of stochastic and deterministic search techniques to generate low-energy ligand poses.[3] The standard protocol for generating a single ligand pose involves a three-stage Genetic Algorithm search (GA1, GA2, GA3), followed by low-temperature Monte Carlo (MC) sampling and Simplex minimization (MIN) steps. The primary scoring function in rDock, denoted as S^{total}, is calculated as a weighted sum of several components: intermolecular interactions (S^{inter}), intramolecular interactions within the ligand (S^{intra}), intramolecular interactions within the binding site (S^{site}), and external restraint terms ($S^{restraint}$):

$$S^{total} = S^{inter} + S^{intra} + S^{site} + S^{restraint} \quad (4)$$

$$S^{inter} = W_{vdw}^{inter}.S_{vdw}^{inter} + W_{polar}^{inter}.S_{polar}^{inter} + W_{repul}^{inter}.S_{repul}^{inter} + W_{arom}^{inter}.S_{arom}^{inter} + W_{solv}.S_{solv} + W_{rot}.N_{rot} + W_{const} \quad (5)$$

$$S^{intra} = W_{vdw}^{intra}.S_{vdw}^{intra} + W_{polar}^{intra}.S_{polar}^{intra} + W_{repul}^{intra}.S_{repul}^{intra} + W_{dihedral}^{intra}.S_{dihedral}^{intra} \quad (6)$$

$$S^{site} = W_{vdw}^{site}.S_{vdw}^{site} + W_{polar}^{site}.S_{polar}^{site} + W_{repul}^{site}.S_{repul}^{site} + W_{dihedral}^{site}.S_{dihedral}^{site} \quad (7)$$

$$S^{restraint} = W_{cavity}.S_{cavity} + W_{tether}.S_{tether} + W_{nmr}.S_{nmr} + W_{ph4}.S_{ph4} \quad (8)$$

The S^{inter} term represents the protein–ligand (or RNA–ligand) interaction score. S^{intra} denotes the change in the ligand's internal energy relative to its input conformation. Similarly, S^{site} reflects the relative energy of the flexible regions within the binding site. The $S^{restraint}$ term comprises a collection of non-physical constraint functions that can be applied during docking calculations.

For validation studies with rDock, the 30S subunit (PDB ID 4v7l) and 50S large subunits (PDB ID 4u24) were prepared using the Protein Preparation Wizard module in Maestro (Schrödinger Release 2024-3), and the receptor was saved in .mol2 format for use in docking calculations. Metal ions were retained as part of the receptor structure. All peptide molecules were prepared using the Maestro-LigPrep module and converted to MDL SDFile (SDF) format prior to docking calculations.

For all docking calculations, 100 poses were generated for each peptide using Glide and rDock to ensure comprehensive sampling of the binding pocket. For the validation of co-crystallized viomycin and dalfopristin ligands, the top-scoring poses were found to be structurally consistent with the experimental binding modes. For all subsequent docking of potential peptide modulators, the top-ranked pose (lowest docking score from Glide GScore and rDock score) was selected for further analysis.

Supplementary tables

Table S1. Residues and nucleotides comprising the putative binding pocket.

G44, G46-U49, G52-G61, G108-C110, G115-G117, G289, G306-G310, C314-G318, C328-G332, G351, G354, A356-U359, C366, C369, U387, G388, C390

Table S2. The details of the MD input structures

Binding site	PDB ID	RNA	Ribosomal proteins	# Mg^{2+} ions	# crystal waters	Net charge	# atoms
**DC**	4v7l	16S rRNA (646 nucleotides)	s12 (124 residues)	74	-	-465	14,884
**PTC**	4u24	23S rRNA (1479 nucleotides)	L2 (74 residues), L3 (104 residues), L4 (53 residues), L13 (125 residues), L14 (122 residues), L15 (54 residues), L16 (136 residues), L20 (63 residues), L21 (24 residues), L22 (110 residues), L32 (25 residues), L34 (46 residues)	143	503	-1025	39,708
**Putative binding pocket**	4v7l	16S rRNA (704 nucleotides)	s4 (208 residues), s12 (124 residues), s16 (83 residues), s17 (99 residues), s20 (99 residues),	55	-	-494	20,154
**B8 bridge**	4v7l	16S rRNA (433 nucleotides) 23S rRNA (349 nucleotides)	L14 (122 residues), L19 (137 residues), s20 (99 residues)	64	-	-597	19,685

Table S3. Molecular docking calculations of **DBAASP v3** peptide candidates for the **decoding center (DC) region**.

**Peptide name / PDB ID**	**Sequence**	**SP-Peptide GScore (kcal/mol)**	**rDock score (kcal/mol)**
Dissulfin-02 (6FGM)	ACFLTRLGT**YV**C	-10.37	-56.88
Dynobactin A (7T3H)	WNSNVHSYRF	-11.49	-55.65
c-RW, Com5 (1QVL)	RRWWRF	-10.52	-54.80
Arylomycin A2 (3IIQ)	xaGXAY	-9.46	-50.16
**Tyrocidine A (6B35)**	fX**FfN**K**YVXL**	-9.94	-49.44
Tsushimycin (1W3M)	DXxXDGDGxVP	-10.80	-49.38
Tyrocidine A (6B34)	fX**FfN**Q**YVXL**	-9.75	-46.90
**Chaxapeptin (2N5C)**	GFGSKPL**DSFGL**NFF	-10.88	-44.36
**Decoralin-NH2 (2N9A)**	SLLS**LIRKLI**T	-10.75	-41.27
Uperolein (2GFR)	XPDPNAFY**GLM**	-9.15	-41.00
VG13P (5WRX)	VARGWG**RK**CPLFG	-10.92	-40.43
Tyrocidine A (4M6E)	fP**FfN**Q**YVXL**	-9.79	-38.72
Melanotropin alpha, alpha-MSH (7F4D)	SYSMEHFRWGKPV	-11.52	-38.11
Temporin-L (6GS5)	FVQWFSKFLGRIL	-9.02	-35.65
Octyl Tridecaptin A1 (2N5Y)	vxGswSXxFEVxA	-10.63	-35.19
Cm-p5 (2MP9)	SRSE**LI**VHQRLF	-9.75	-34.85
**Neurokinin A (1N6T)**	HKT**DSF**V**GLM**	-10.87	-34.58
Cm-p1 (6CTG)	SRSE**LI**VHQR	-10.45	-34.13
TetraF2W-RK-AMD, WW291 (6NM2)	WWWL**RK**IW	-13.14	-33.58

*Non-standard amino acids are represented by letter X.
*Lowercase letters indicate a modified form of a standard amino acid.

Table S4. Molecular docking calculations of **CyclicPepedia** and **CycPeptMPDB** peptide candidates for the **decoding center (DC) region**.

Peptide library name	Peptide name / PDB ID	Sequence	SP-Peptide GScore (kcal/mol)	rDock score (kcal/mol)
	CP01246	YCG**FC**	-8.04	-30.64
	CP00153	GPGPGA	-7.93	-29.25
	CP00076	YKG	-7.61	-31.10
**CyclicPepedia**	**CP01663**	VK**YA**G	-7.32	-32.07
	CP00080	**YA**FN	-7.27	-30.13
	**CP01126**	RGD**FC**	-7.10	-30.64
	CP01660	**YA**FSG	-7.00	-31.90
	**2508**	['P', 'Y', 'Mono83']	-6.44	-32.02
	**2289**	['K', 'N', 'S', 'Y', 'F']	-7.76	-31.73
**CycPeptMPDB**	2511	['dS', 'V', 'Mono85']	-6.12	-29.50
	2499	['dA', 'L', 'Mono90']	-6.33	-28.54
	2483	['A', 'L', 'Mono87']	-6.08	-28.48

Table S5. Molecular docking calculations of **DBAASP v3** peptide candidates for the **peptidyl transferase center (PTC) region**.

**Peptide name / PDB ID**	**Sequence**	**SP-Peptide GScore (kcal/mol)**	**rDock score (kcal/mol)**
Dynobactin A (7T3H)	WNSNVHSY**RF**	-15.16	-54.87
**WW291 (6NM2)**	**WWWLRKIW**	-16.22	-47.53
Tyrocidine A (6B34)	fX**FfN**Q**YVXL**	-13.96	-46.58
PW2 (1M02)	HP**LK**QY**WW**RPSI	-14.96	-44.72
**Tyrocidine A (6B35)**	fX**FfN**K**YVXL**	-14.98	-44.69
WW295 (6NM3)	**RKIWWWWL**	-15.36	-42.00
Temporin A (2MAA)	FLPLIGRVLSG**IL**	-14.20	-41.00
Daptomycin (1XT7)	WnDTGXDaDGsXX	-15.64	-39.92
**Peptide 536_2 (6RRO)**	**GF**IVK**RF**K**IL**V	-14.59	-37.86
Agelaia-MP (7JGX)	**IFWL**FRGKADVAL	-15.29	-37.82
Chaxapeptin (2N5C)	**GF**GSKPLDSFGLNFF	-16.66	-36.73
Temporin-B (6GIL)	LLPIVGNL**LK**SLL	-13.84	-35.60
**Protonectin (7JHF)**	**IF**GT**ILGFLK**GL	-14.35	-35.58
**Hemagglutinin (2L24)**	**IF**GAIA**GF**IKN**IW**	-13.57	-35.20
Decoralin-NH2 (2N9A)	SLLSLI**RK**LIT	-13.11	-33.50
Protegrin 1 (2MQ5)	LYRR**RF**VVGR	-14.42	-32.65
BPTI (7M77)	G**IL**DIKNKVSNLF	-14.31	-32.50

Table S6. Molecular docking calculations of **CyclicPepedia and CycPeptMPDB** peptide candidates for the peptidyl transferase center (**PTC**) region.

Peptide library name	Peptide name / PDB ID	Sequence	SP-Peptide GScore (kcal/mol)	rDock score (kcal/mol)
**CyclicPepedia**	**CP01457**	AVT-CO	-10.50	-54.29
	CP01968	AGF	-9.64	-53.90
	**CP00667**	PRGTK	-9.50	-54.40
	**CP00937**	Y-Pen-GF-Pen	-9.09	-61.03
**CycPeptMPDB**	2470	['A', 'L', 'Mono77']	-10.25	-49.50
	2504	['Me_dA', 'L', 'Mono90']	-10.21	-50.46
	**2508**	['P', 'Y', 'Mono83']	-10.19	-55.91
	**2476**	['L', 'A', 'Mono78']	-10.09	-49.28
	1122	['Mono35', 'G', 'L', 'G']	-9.97	-50.18
	**2510**	['dY', 'L', 'Mono85']	-9.80	-50.18
	2490	['meA', 'L', 'Mono78']	-9.62	-50.12
	**2289**	['K', 'N', 'S', 'Y', 'F']	-9.33	-50.33

Table S7. Molecular docking calculations of **DBAASP v3, CyclicPepedia, and CycPeptMPDB** peptide candidates for the **putative binding pocket**.

Peptide library name	Peptide name / PDB ID	Sequence	SP-Peptide GScore (kcal/mol)	rDock score (kcal/mol)
**DBAASP v3**	**Arylomycin A2 (3IIQ)**	xaGXAY	-10.09	-61.44
	**c-RW, Com5 (1QVL)**	RRWWRF	-10.25	-56.25
	**Viomycin (4V7L)**	XXSSXX	-9.17	-56.06
**CyclicPepedia**	CP00127	YTY	-8.03	-68.30
	CP02162	KFAV	-8.32	-61.70
	**CP01970**	**PG**LA	-8.24	-57.43
	CP00934	YSY	-7.83	-61.99
	**CP01953**	**PG**LG	-7.70	-57.46
	CP00224	KP	-7.48	-62.64
	**CP00153**	G**PGPG**P	-7.13	-59.92
**CycPeptMPDB**	2513	['P', 'Y', 'Mono85']	-8.34	-50.64
	**1902**	['K', 'N', 'S', 'dY', 'F']	-8.19	-53.05
	**2510**	['dY', 'L', 'Mono85']	-7.80	-50.83
	**2508**	['P', 'Y', 'Mono83']	-7.69	-58.50

Table S8. Molecular docking calculations of **DBAASP v3, CyclicPepedia, and CycPeptMPDB** peptide candidates for the **B8 bridge**.

Peptide library name	Peptide name / PDB ID	Sequence	SP-Peptide GScore (kcal/mol)	rDock score (kcal/mol)
**DBAASP v3**	**Arylomycin A2 (3IIQ)**	xaGXAY	-7.84	-49.59
	**c-RW, Com5 (1QVL)**	RRWWRF	-10.35	-28.77
**CyclicPepedia**	CP00224	KP	-8.18	-26.73
	**CP02321**	KFX	-7.83	-27.84
	**CP00153**	GPGPGP	-7.39	-26.02
	CP02342	WPA	-7.23	-27.15
**CycPeptMPDB**	1898	['Lys(Me2)', 'N', 'Ser(Me)', 'Tyr(Me)', 'N']	-8.48	-20.25
	2289	['K', 'N', 'S', 'Y', 'F']	-8.47	-22.01
	2512	['dL', 'S', 'Mono85']	-8.19	-20.45
	2479	['F', 'A', 'Mono86']	-7.77	-22.84
	**2508**	['P', 'Y', 'Mono83']	-7.72	-24.80
	1885	['K', 'A', 'S', 'Y', 'F']	-7.63	-21.15
	1123	['Mono35', 'G', 'L', 'A']	-7.61	-19.38
	**2510**	['dY', 'L', 'Mono85']	-7.48	-18.97
	2487	['dA', 'dL', 'Mono87']	-7.32	-19.99
	2504	['Me_dA', 'L', 'Mono90']	-7.27	-19.68
	2497	['dA', 'meL', 'Mono78']	-7.01	-19.62

Table S9. Residues/nucleotides with high betweenness values (top 5% of the distribution) in the 30S small subunit.

Subunit	Component	Nucleotides/Residues
30S ssu	16S rRNA	G6-A7, G9, G11-U12, G39, C43, A53, A59, A66, C110, U114, A129, C132, C135, C136, A223, C308, C330-G331, U333-C334, C339, G347-G348, G350, C352, G354, A356, A366, G376-G380, G388-A389, C392-A393, C396, A408, C488, A499-C501, C511-C513, C526, A533-U534, G541, U543, G597, A607-A608, U610-C612, C620-A621, C623-C624, G628, A630-U632, U636, A663, U678, A681, G698, G713, A715-A716, G721, G774, G776, U793, U813, A816, G829-G830, G833-U834, A865-C866, C876, A909, C932, U1056-C1059, G1061, G1068-U1075, A1092-U1095, C1096-C1100, G1108, C1112-C1114, G1127-C1128, C1149-A1157, A1180-U1183, C1203-G1207, G1241-G1244, A1269-A1271, A1280-A1285, U1326-A1329, A1360-A1362, U1380-U1381, C1384, C1388-U1390, G1392, G1415, U1440, C1460-G1461, U1463, G1529
	S2	R20, G70, T71, L160, F161, I163, E174, G179, P181, V182, F183, A184, I185, D187, T188
	S4	K7, L8, K9, R61, L81, G86, A91, L92, G95, Q115, L116, V117, K120, A121, I131, V136
	S5	V17, A34, V37, V38, G41, N42, G43, R44, Y49, G50, K51, A52, E54, A62, M63, R67, I71, K85, V87, T89, G90, S91, Q96, S99, T102, I105, V113, L114, E115, V116, V119, H120, N121, G128, I133, N134, N145
	S7	V5, F25, L29, T37, A38, G81, S82, Q85, V86, R91, R94, A97, K148
	S8	T11, R12, Q17, N20, K21, A36, N37, V71, E72, Q75, V77, E90, L91, S106, G108, V109
	S9	R11, S14, A15, G68, G69, A77, H80, T104, R105
	S10	A12, F13, H15, L17, I18, P41, L42, R45, T69
	S11	A24, P59, G87, P88
	S13	G5, V24, G25, R28, E58, V59
	S14	K2, E9, N61, R62, G67
	S16	R5, L6, R8, K12, R14, P15, Q18, V19, V21, N26, G37, A65, T66
	S18	S41, A55, R56, I58
	S20	K4, A16, R28, T29, A62
	P-tRNA	U65
	mRNA	C16

Table S10. Residues/nucleotides with high betweenness values (top 5% of the distribution) in the 50S large subunit.

Subunit	Component	Nucleotides/Residues
**50S lsu**	23S rRNA	A64, A71, C129, A131, G169, U206, A226, C236, G259, C274, G277, G315, C357, G396, G400, A402, G408, C413, U419, A429, A460, A627, U642, G682, U686, G822, C835, U846-U847, C876, G883, U929-G930, U932, A975, U1083, A1090, G1093-U1094, U1184, A1213, G1381-G1382, U1438, G1519, C1771, U1820, G1831, C1833, U1846, U1917, C1925, U2106, U2111, C2207-U2210, A2241, U2243, G2250, U2259, C2260, C2263, G2269, G2293, C2310, U2320, C2342, C2359, A2366, A2439, A2451, G2458, G2487, U2489, C2496, A2541, G2550-C2551, U2555, U2584-U2585, A2654, A2670-G2671, G2673-G2674, A2740, C2745, A2750, C2774, A2887, G2890, A2893
	5S rRNA	G61, U65, C68
	L1	V39, A45, V46, N47, A84, E85, A86, M97, E98, I104, K105, K106, G107, R134, T173, T174, I175, G176, T202, Q203, G206, V207, Y208, I209
	L2	Q116, P125, A157, V171, R174, V194, L201, R213, T256
	L3	V9, K55, A132, T133, P205
	L4	E2, K6, T18, A192, V193
	L5	R109, G115, A118, D122, G123, N126, M129, V148, G150
	L6	P125
	L9	T124
	L11	V97
	L13	R116
	L14	R49, A60, A83, N90, S91, T97, R105, S117
	L16	G29, I63, V67, P72, E90, K123
	L17	R86
	L18	A23, T31, H34
	L19	W30, T59, L96, R100, N114
	L20	K15-I16
	L27	G64
	L28	T24, L32
	L30	E38, N48
	L36	C27, A29, K34-Q35

Table S11. Effect of the hubs on the flow of information through the shortest pathway between the B8 inter-subunit bridge and the 30S shoulder.

**Hub residues**	**Betweenness score, C_B**	**Allosteric coupling $C_{ac,ij}$ change (%)**	**Local efficiency, E_{loc}**	**Global efficiency E_{glob} change (%)**
G351 (h14)	0.071	-0.35	8.94	-0.05
C352 (h14)	0.058	-0.35	7.54	-0.02
G354 (h14)	0.059	-0.35	6.95	-0.02
C355 (h14)	0.054	-0.35	6.33	-0.02
A356 (h14)	0.049	-0.35	6.84	-0.03
U367 (h15)	0.046	-1.27	7.30	-0.03
C395 (h15)	0.023	-0.20	7.84	-0.01
A441 (h17)	0.021	-0.20	15.67	-0.01
C440 (h17)	0.023	-0.20	16.75	-0.01
K120 (s4)	0.019	-0.01	20.99	-0.01

Supplementary figures

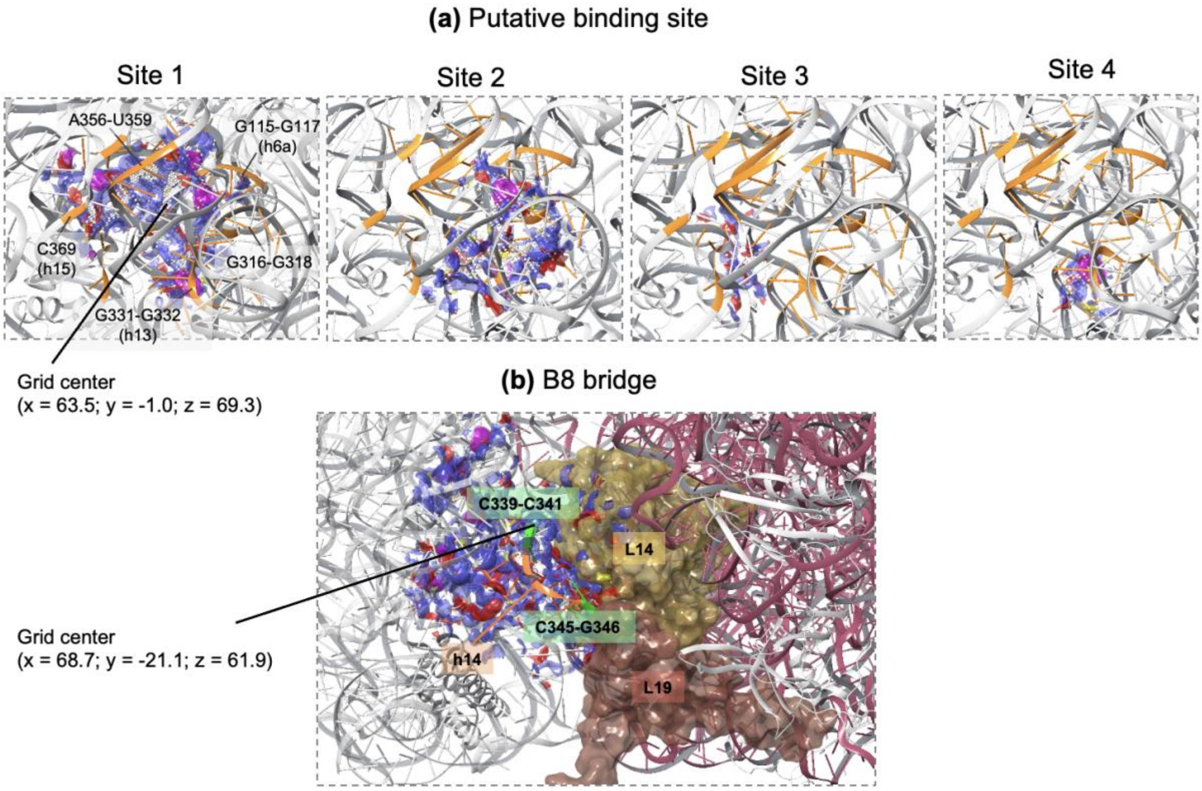

Figure S1. SiteMap-generated pockets for **(a)** the putative binding pocket and **(b)** the B8 bridge. Key nucleotides previously identified through GNM fast mode analysis[4] for the putative binding pocket are shown in light orange. The helix h14 of 16S rRNA is shown in dark orange, and nucleotides that contribute to the formation of the B8 bridge are marked in green. The grid center for each site is indicated by black arrows.

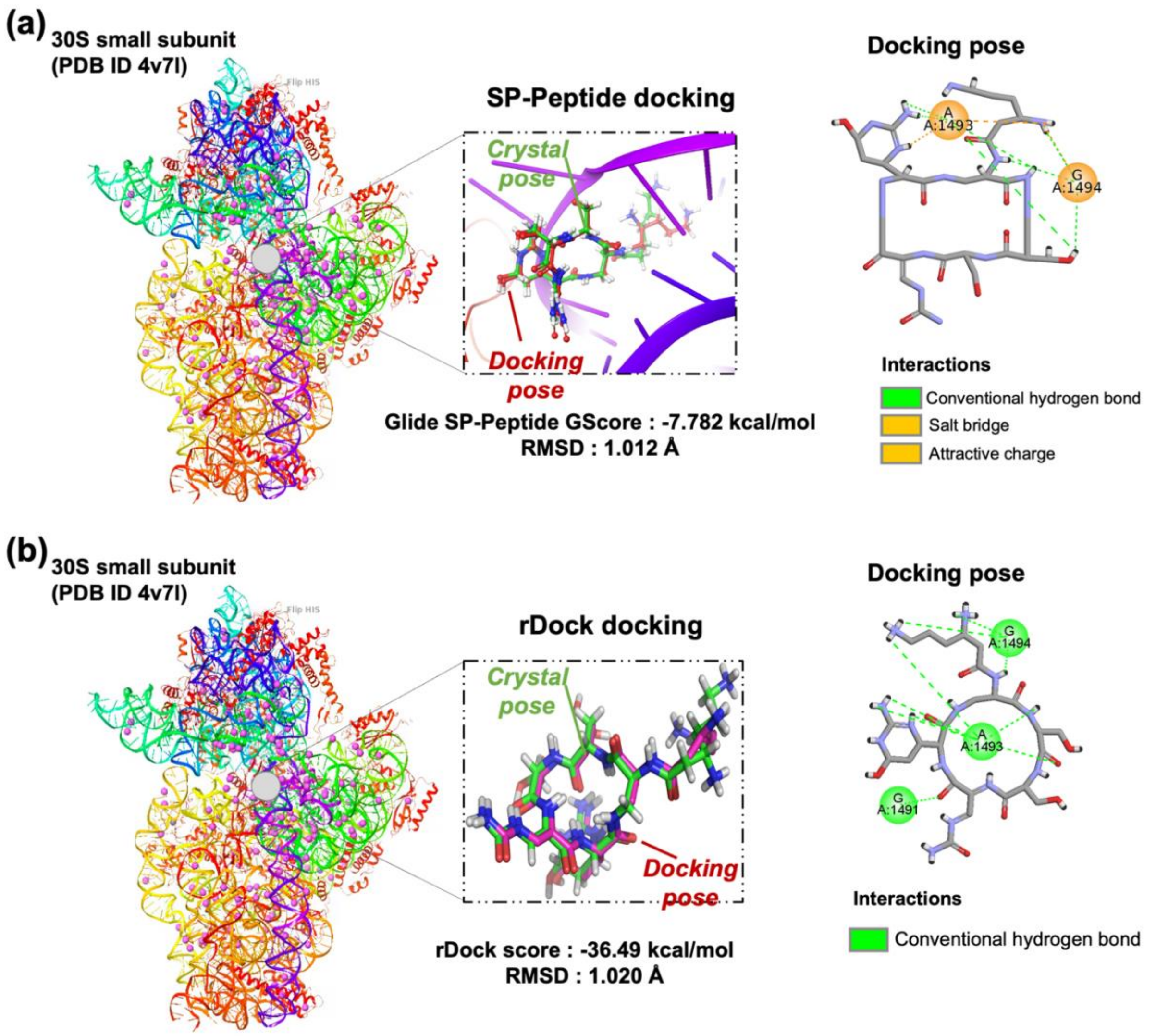

Figure S2. (a) Comparison of the best SP-Peptide docking pose of viomycin, obtained from Glide SP-Peptide docking validation, with its crystallographic pose (crystal pose: green; best SP-Peptide docking pose: red). Two-dimensional interaction diagrams illustrating viomycin–ribosome interactions for the best SP-Peptide docking pose are also provided. **(b)** Comparison of the best rDock docking pose of viomycin, obtained from rDock docking validation, with its crystallographic pose (crystal pose: green; best rDock docking pose: red). Two-dimensional interaction diagrams depicting viomycin–ribosome interactions for the best rDock docking pose are also shown.

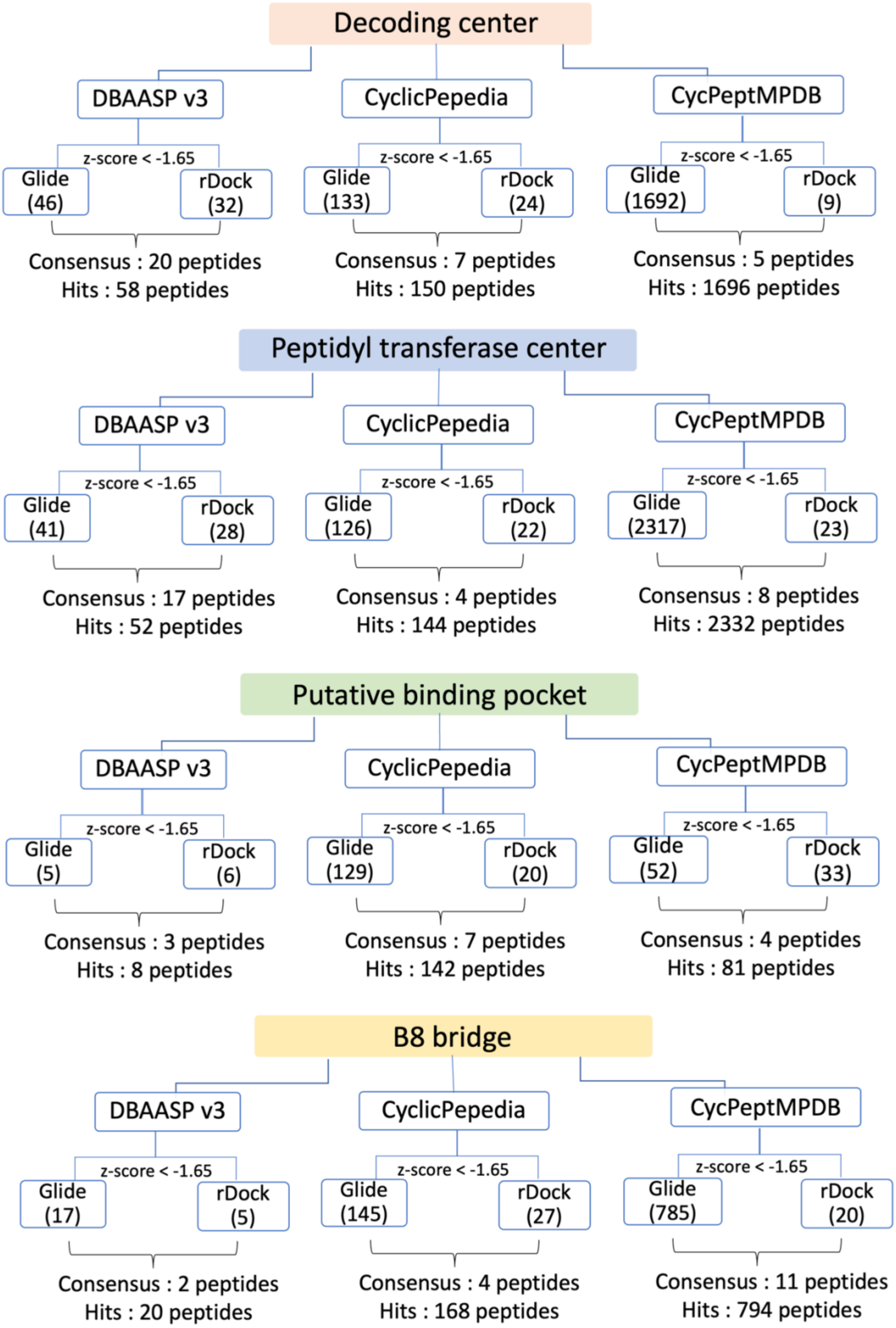

Figure S3. The workflow summarizing the virtual screening of three peptide libraries against the orthosteric sites (DC and PTC), the putative binding pocket, and the B8 bridge of the bacterial ribosome.

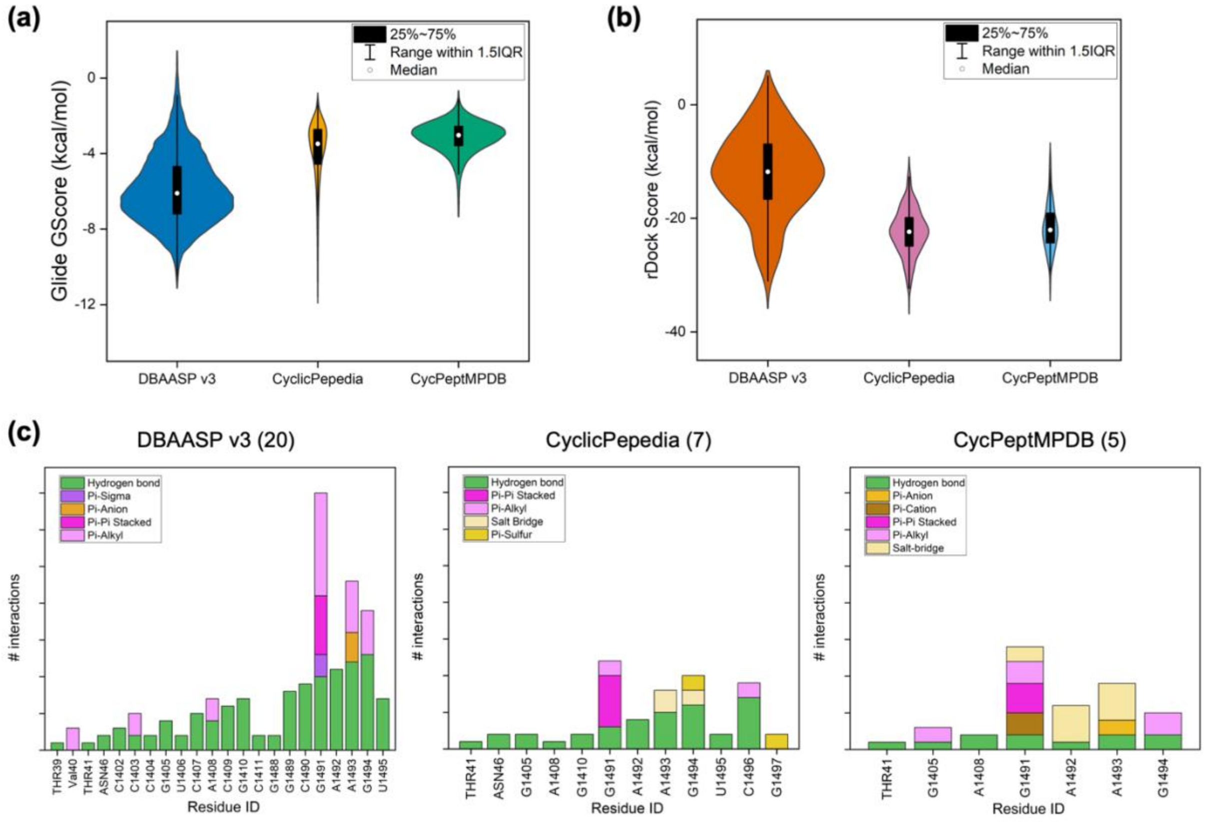

Figure S4. Comparative analysis of peptide datasets based on docking scores and 2D interaction profiles within the DC region. **(a)** Violin plots of Glide GScore values and **(b)** rDock docking scores for peptides derived from the DBAASP v3, CyclicPepedia, and CycPeptMPDB databases. **(c)** Bar plots illustrating the frequency and types of peptide–ribosome interactions—including hydrogen bonds, π-interactions, and salt bridges—for selected peptides through consensus docking of Glide and rDock filtered for z-scores below -1.65.

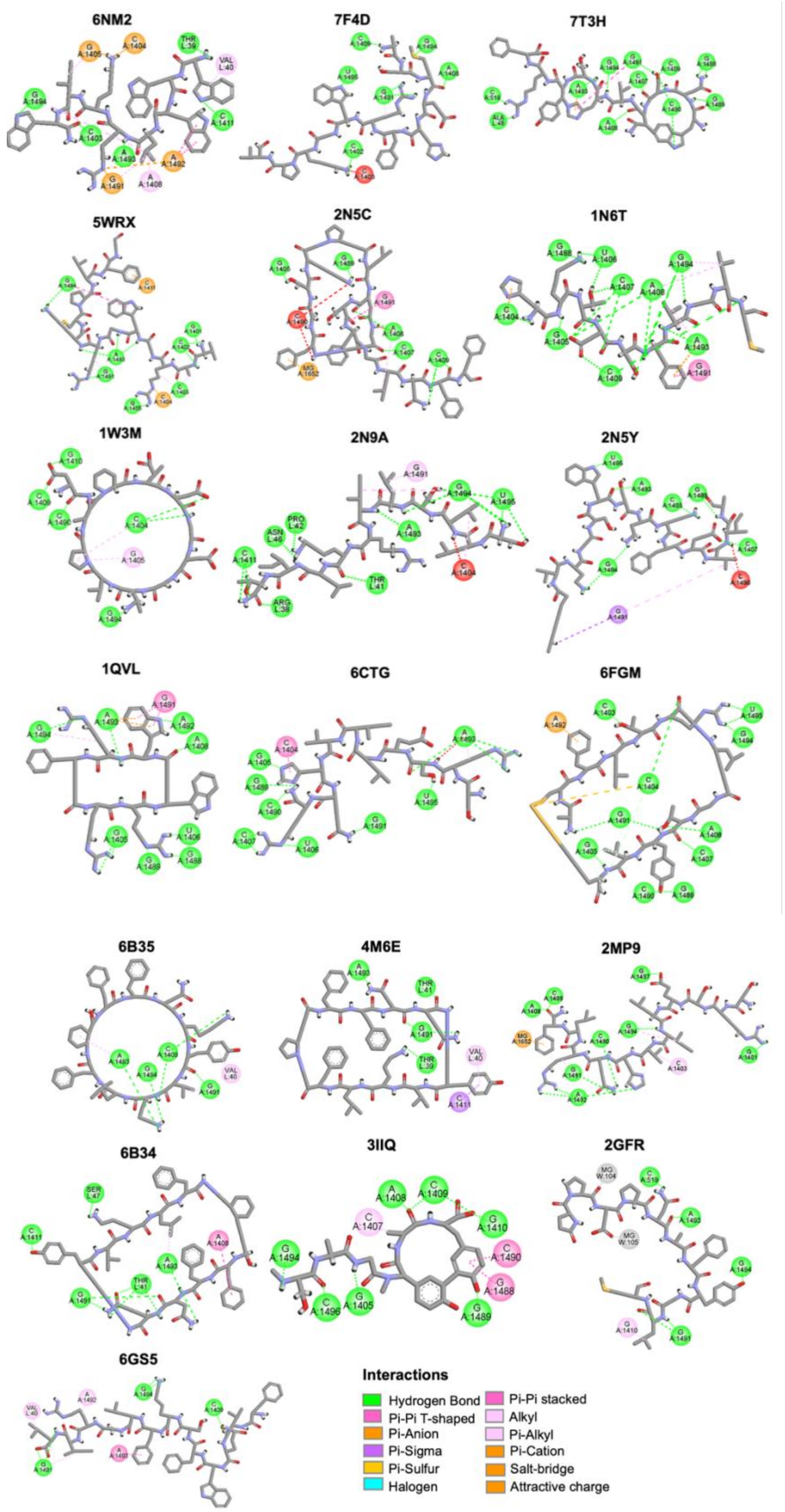

Figure S5. 2D interaction maps for DBAASP v3 hit peptides for the DC region obtained from Glide SP-Peptide docking. Only polar hydrogens are displayed on 2D interaction maps. The non-bonded interactions between the ligand and the surrounding nucleotides based on the color-codes are displayed with dashed lines on 2D maps.

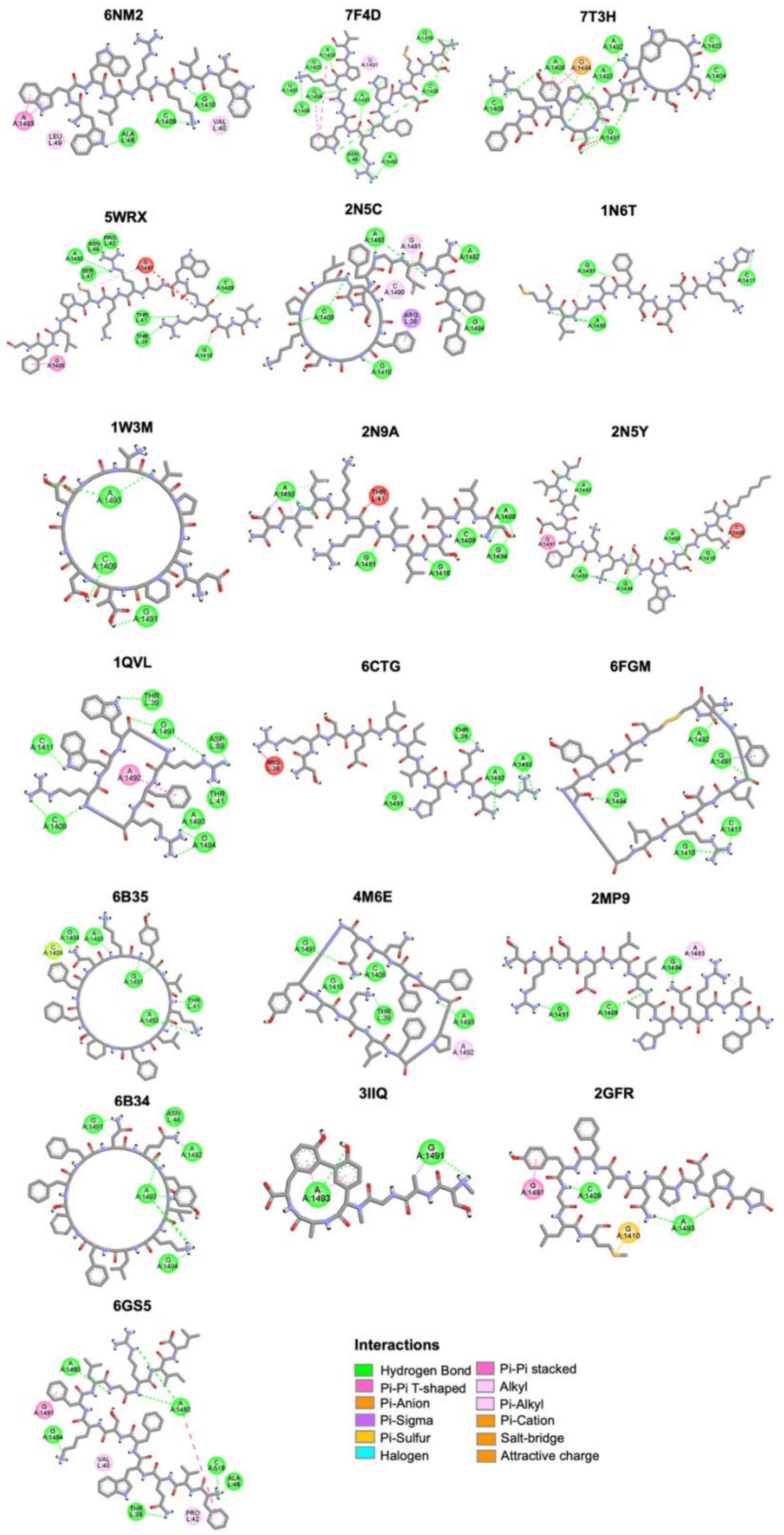

Figure S6. 2D interaction maps for DBAASP v3 hit peptides for the DC region obtained from rDock docking. Only polar hydrogens are displayed on 2D interaction maps. The non-bonded interactions between the ligand and the surrounding nucleotides based on the color-codes are displayed with dashed lines on 2D maps.

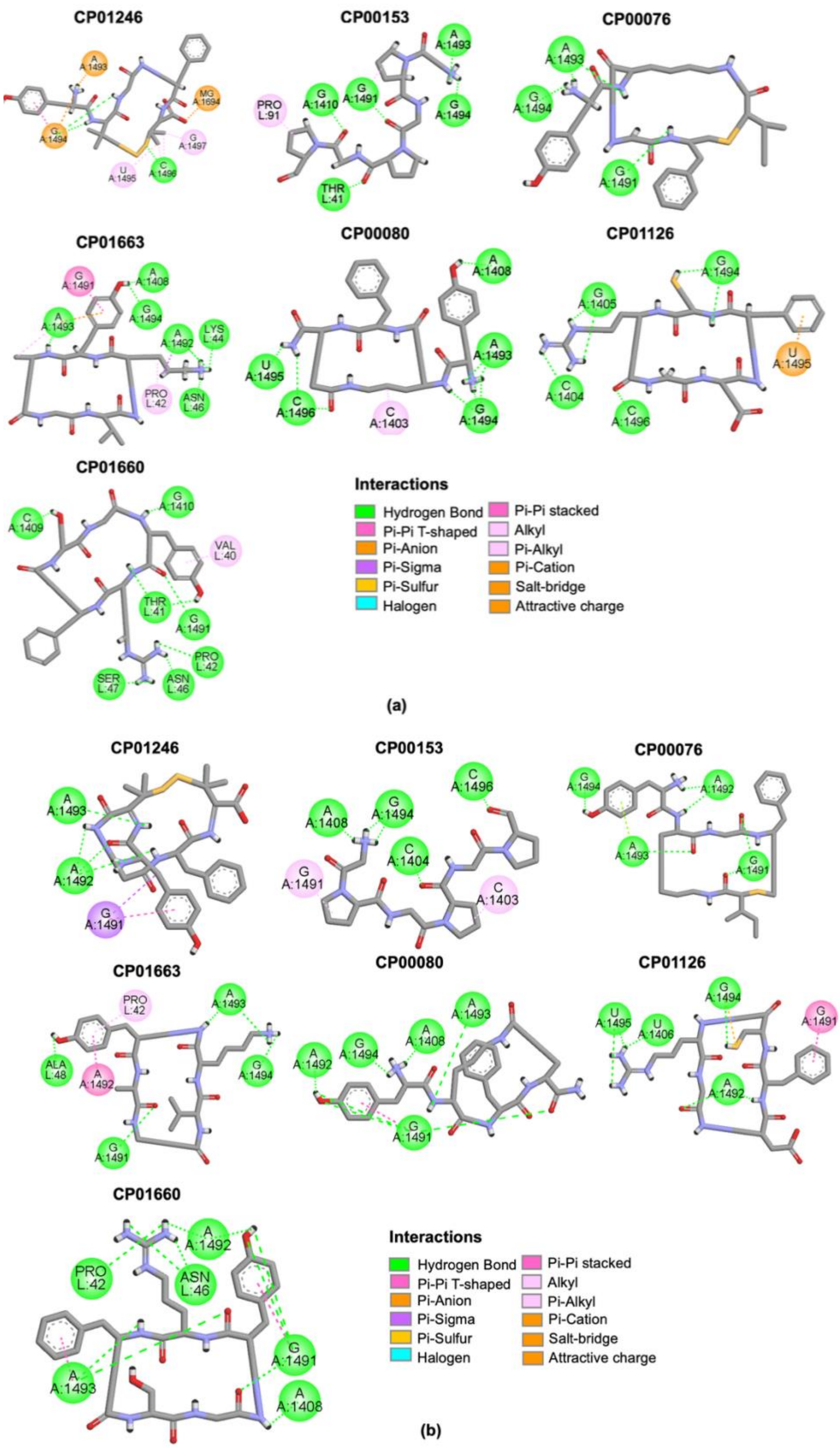

Figure S7. 2D interaction maps for CyclicPepedia hit peptides for the DC region obtained from **(a)** Glide SP-Peptide and **(b)** rDock docking. Only polar hydrogens are displayed on 2D interaction maps. The non-bonded interactions between the ligand and the surrounding nucleotides based on the color-codes are displayed with dashed lines on 2D maps.

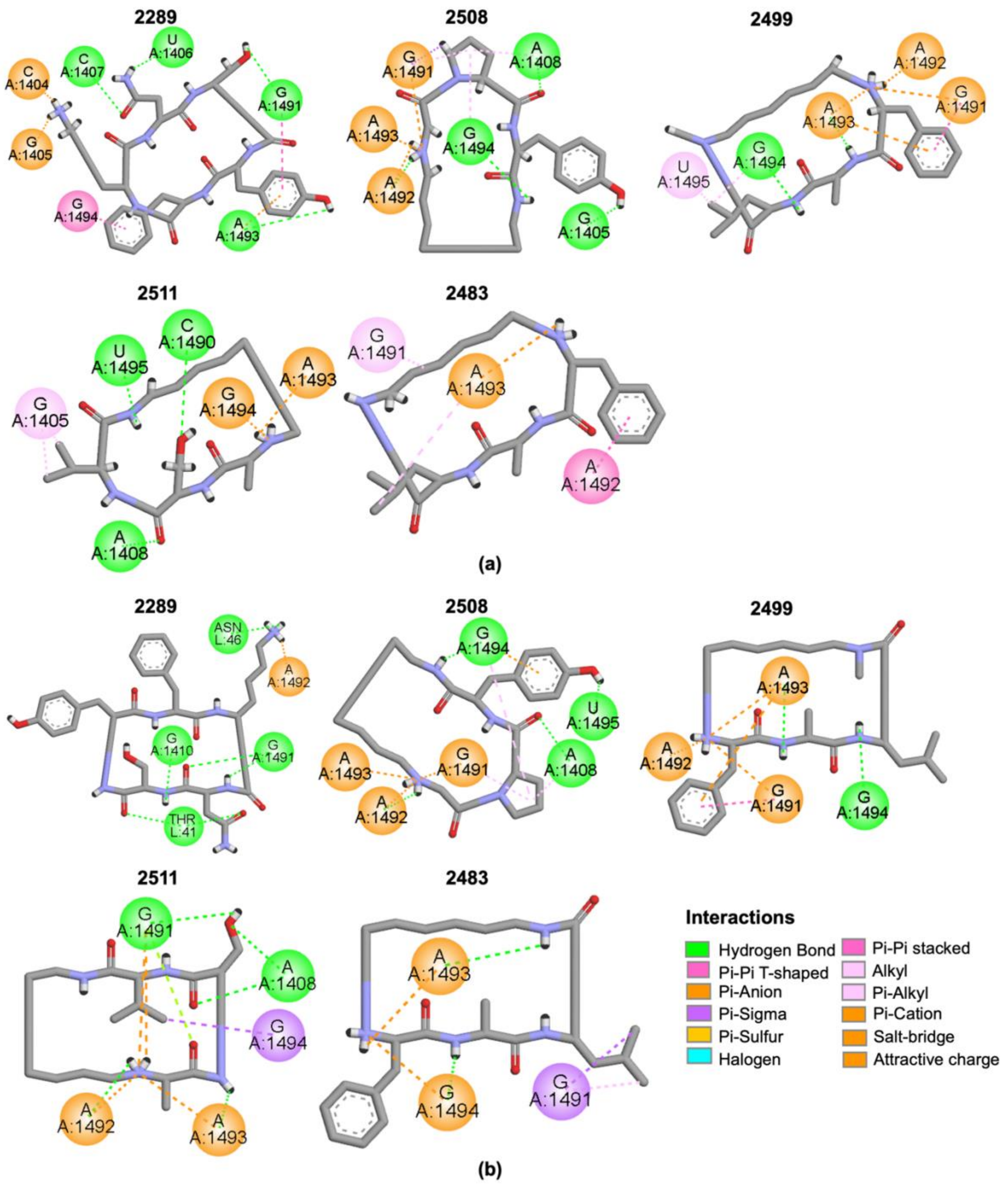

Figure S8. 2D interaction maps for CycPeptMPDB hit peptides for the DC region obtained from **(a)** Glide SP-Peptide and **(b)** rDock docking. Only polar hydrogens are displayed on 2D interaction maps. The non-bonded interactions between the ligand and the surrounding nucleotides based on the color-codes are displayed with dashed lines on 2D maps.

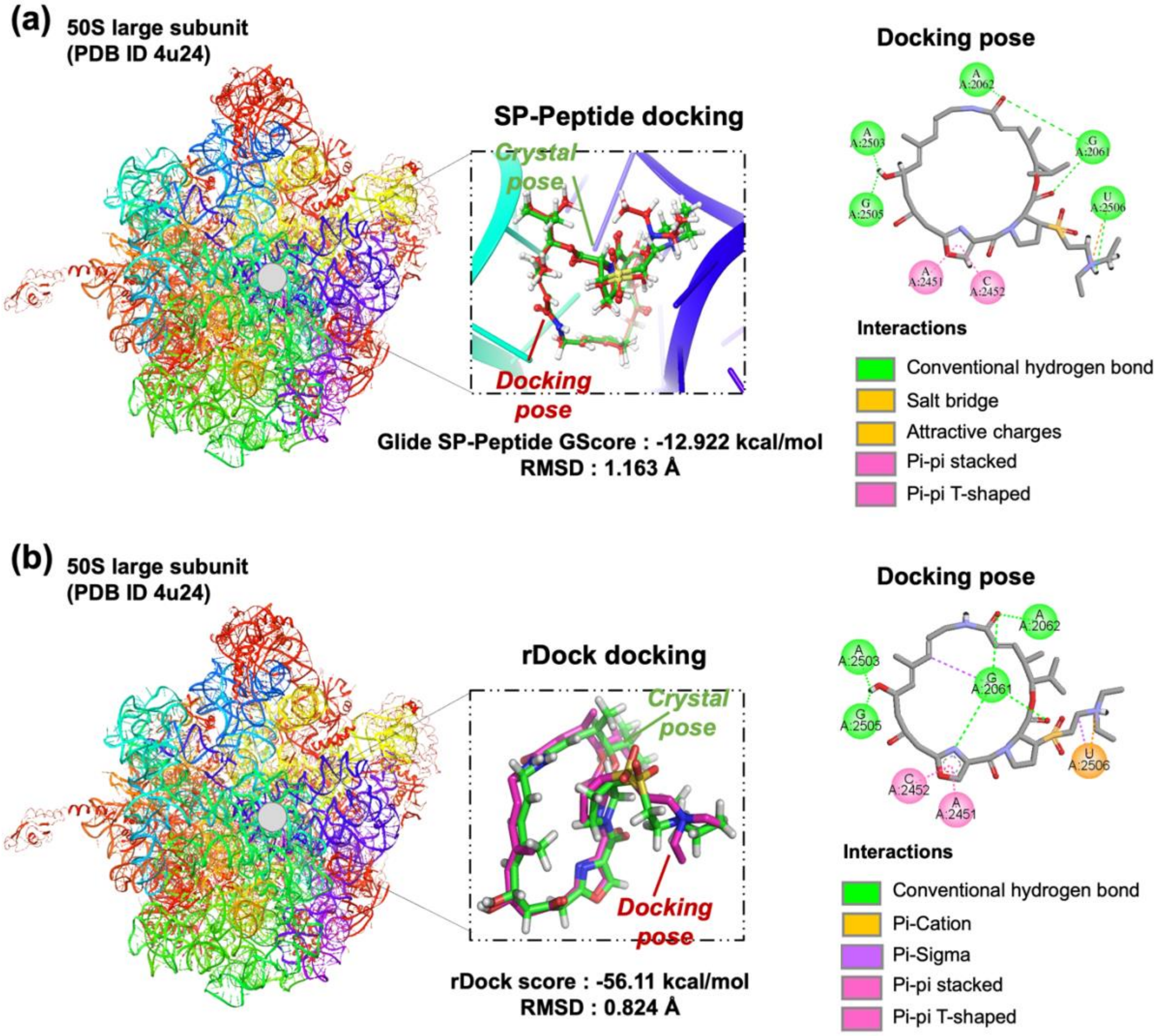

Figure S9. (a) Comparison of the best SP-Peptide docking pose of dalfopristin, obtained from Glide SP-Peptide docking validation, with its crystallographic pose (crystal pose: green; best SP-Peptide docking pose: red). Two-dimensional interaction diagrams illustrating dalfopristin–ribosome interactions for the best SP-Peptide docking pose are also provided. **(b)** Comparison of the best rDock docking pose of dalfopristin, obtained from rDock docking validation, with its crystallographic pose (crystal pose: green; best rDock docking pose: red). Two-dimensional interaction diagrams depicting dalfopristin–ribosome interactions for the best rDock docking pose are also shown.

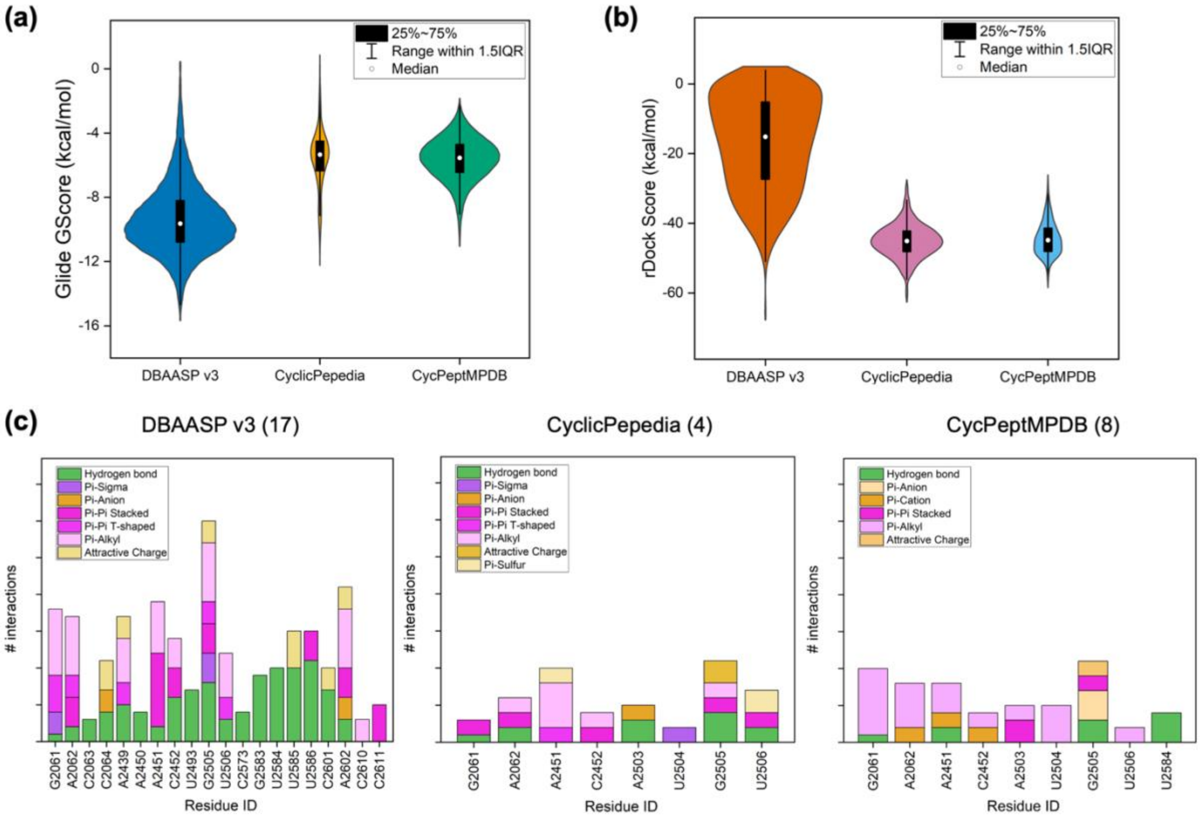

Figure S10. Comparative analysis of peptide datasets based on docking scores, amino acid positional frequencies, and 2D interaction profiles within the PTC region. **(a)** Violin plots of Glide GScore values and **(b)** rDock docking scores for peptides derived from the DBAASP v3, CyclicPepedia, and CycPeptMPDB databases. **(c)** Bar plots illustrating the frequency and types of peptide–ribosome interactions—including hydrogen bonds, π-interactions, and salt bridges—for selected peptides through consensus docking of Glide and rDock filtered for z-scores below -1.65.

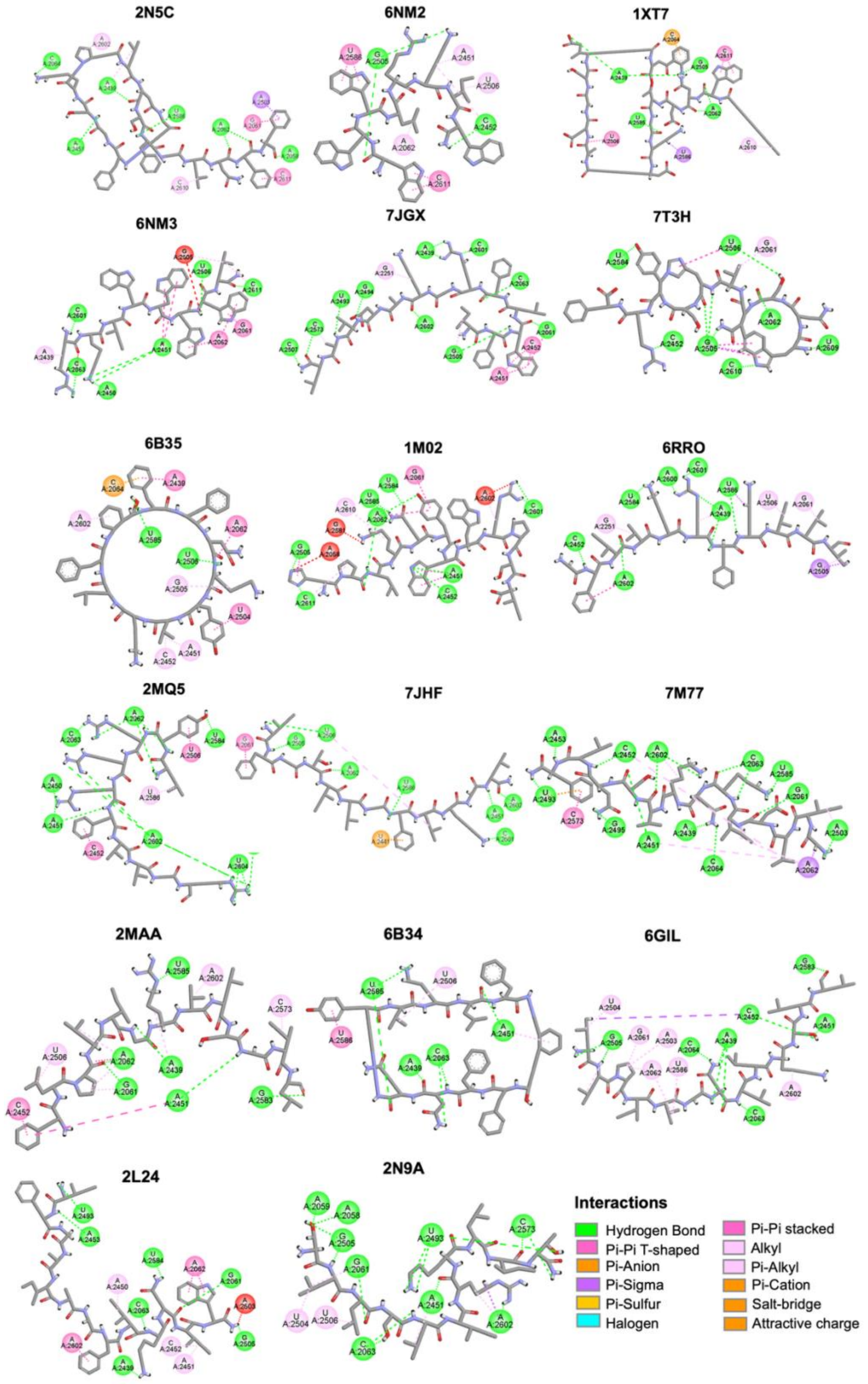

Figure S11. 2D interaction maps for DBAASP v3 hit peptides for the PTC region obtained from Glide SP-Peptide docking. Only polar hydrogens are displayed on 2D interaction maps. The non-bonded interactions between the ligand and the surrounding nucleotides based on the color-codes are displayed with dashed lines on 2D maps.

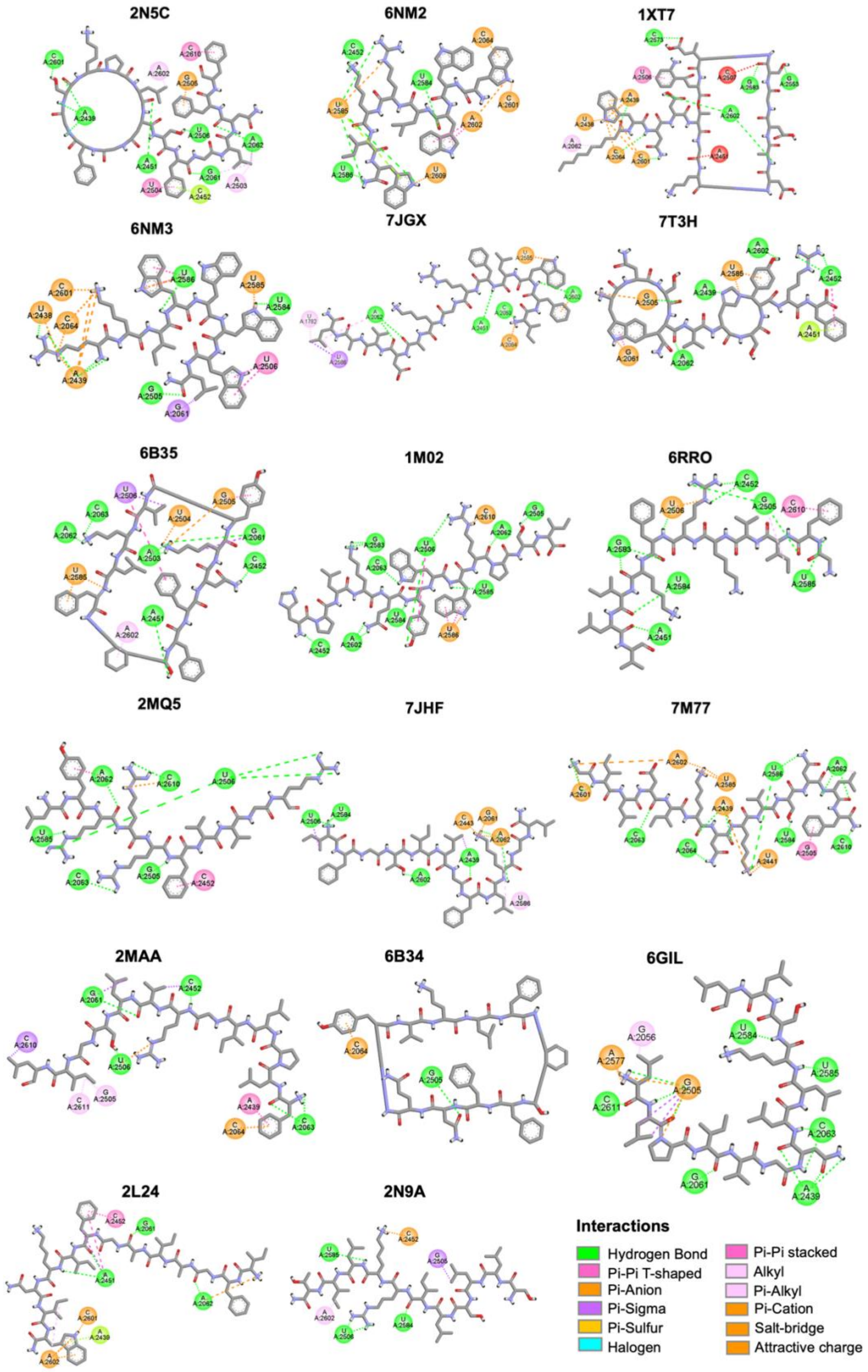

Figure S12. 2D interaction maps for DBAASP v3 hit peptides for the PTC region obtained from rDock docking. Only polar hydrogens are displayed on 2D interaction maps. The non-bonded interactions between the ligand and the surrounding nucleotides based on the color-codes are displayed with dashed lines on 2D maps.

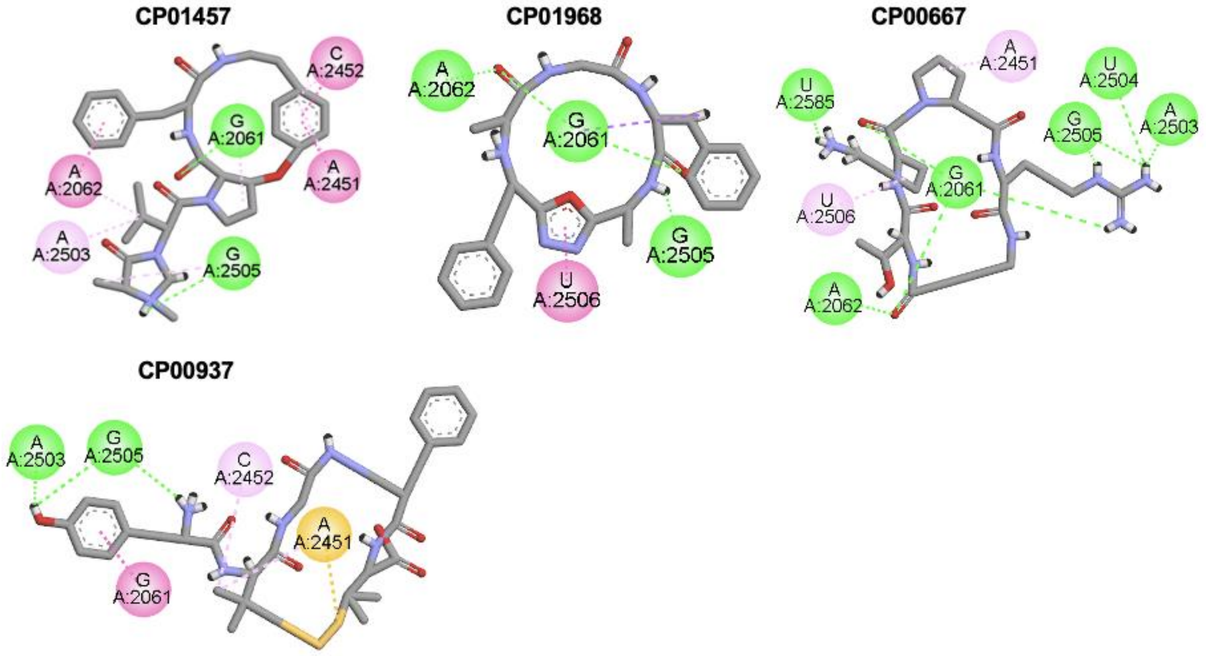

(a)

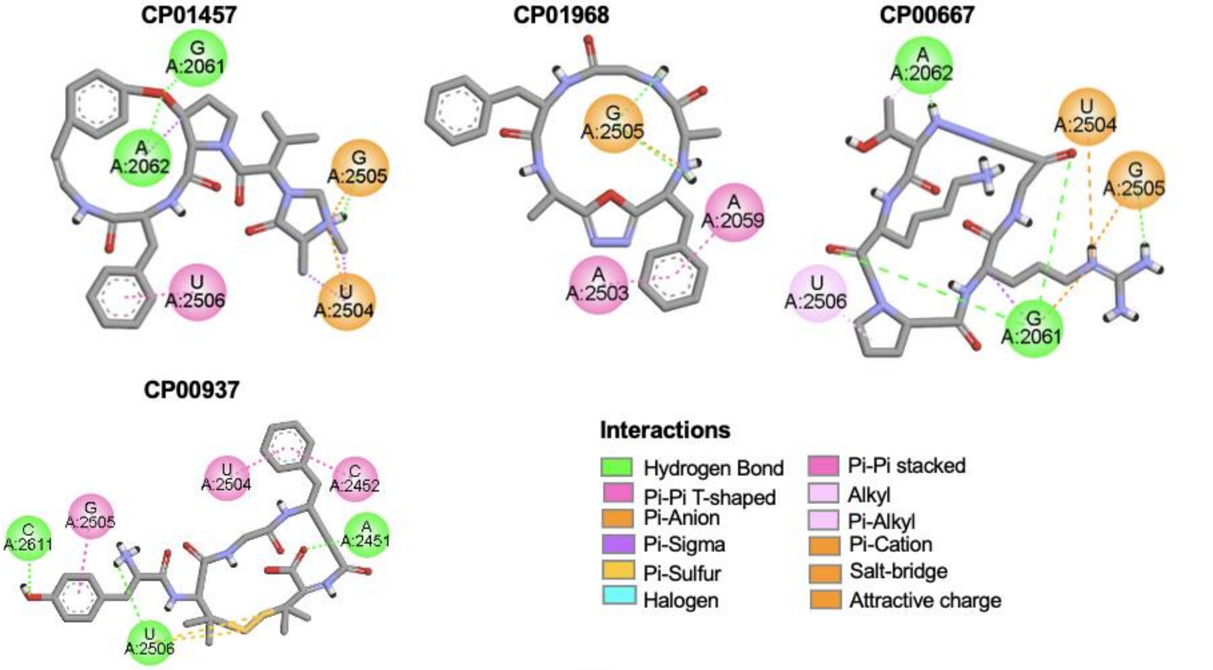

(b)

Figure S13. 2D interaction maps for CyclicPepedia hit peptides for the PTC region obtained from **(a)** Glide SP-Peptide and **(b)** rDock docking. Only polar hydrogens are displayed on 2D interaction maps. The non-bonded interactions between the ligand and the surrounding nucleotides based on the color-codes are displayed with dashed lines on 2D maps.

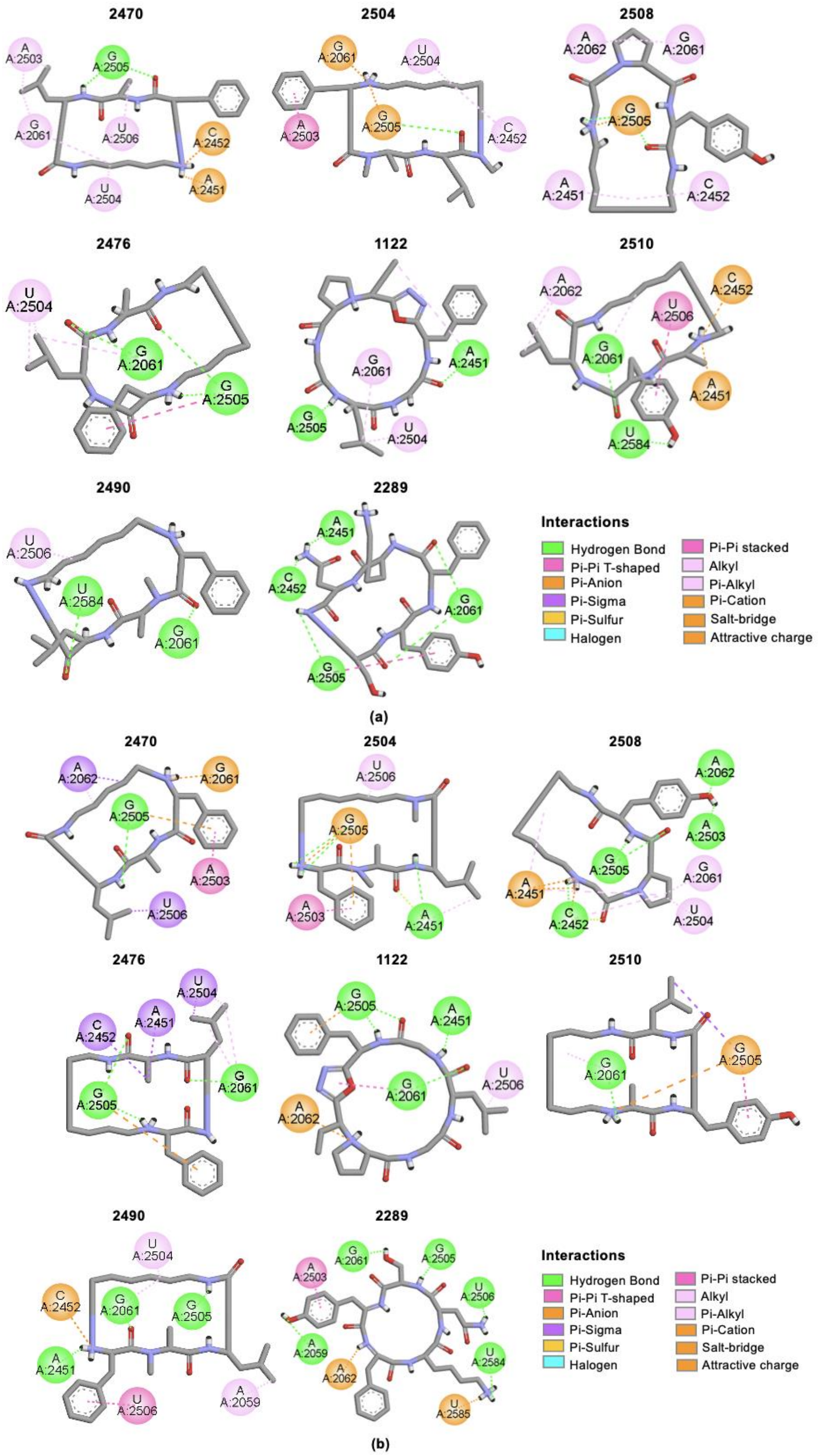

Figure S14. 2D interaction maps for CycPeptMPDB hit peptides for the PTC obtained from **(a)** Glide SP-Peptide and **(b)** rDock docking. Only polar hydrogens are displayed on 2D interaction maps. The non-bonded interactions between the ligand and the surrounding nucleotides based on the color-codes are displayed with dashed lines on 2D maps.

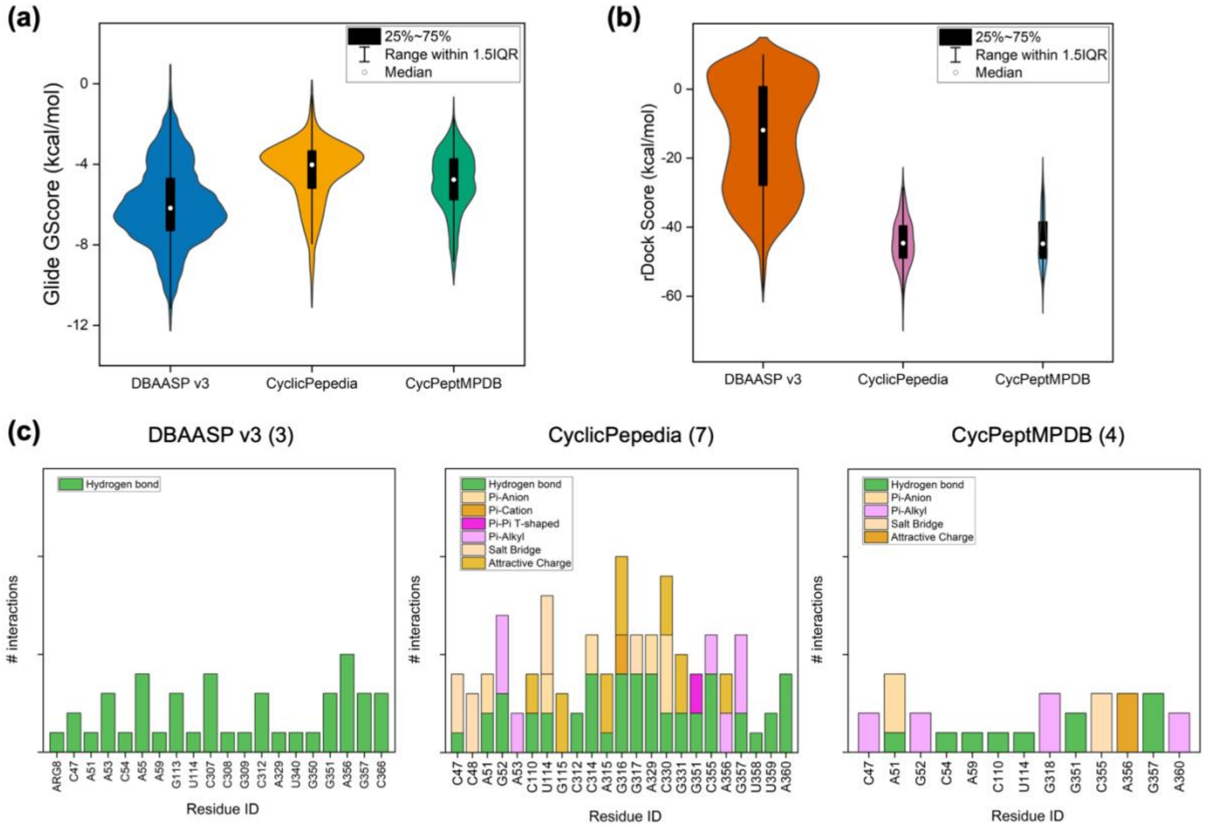

Figure S15. Comparative analysis of peptide datasets based on docking scores, amino acid positional frequencies, and 2D interaction profiles within the putative binding pocket. **(a)** Violin plots of Glide GScore values and **(b)** rDock docking scores for peptides derived from the DBAASP v3, CyclicPepedia, and CycPeptMPDB databases. **(c)** Bar plots illustrating the frequency and types of peptide–ribosome interactions—including hydrogen bonds, π-interactions, and salt bridges—for selected peptides through consensus docking of Glide and rDock filtered for z-scores below -1.65.

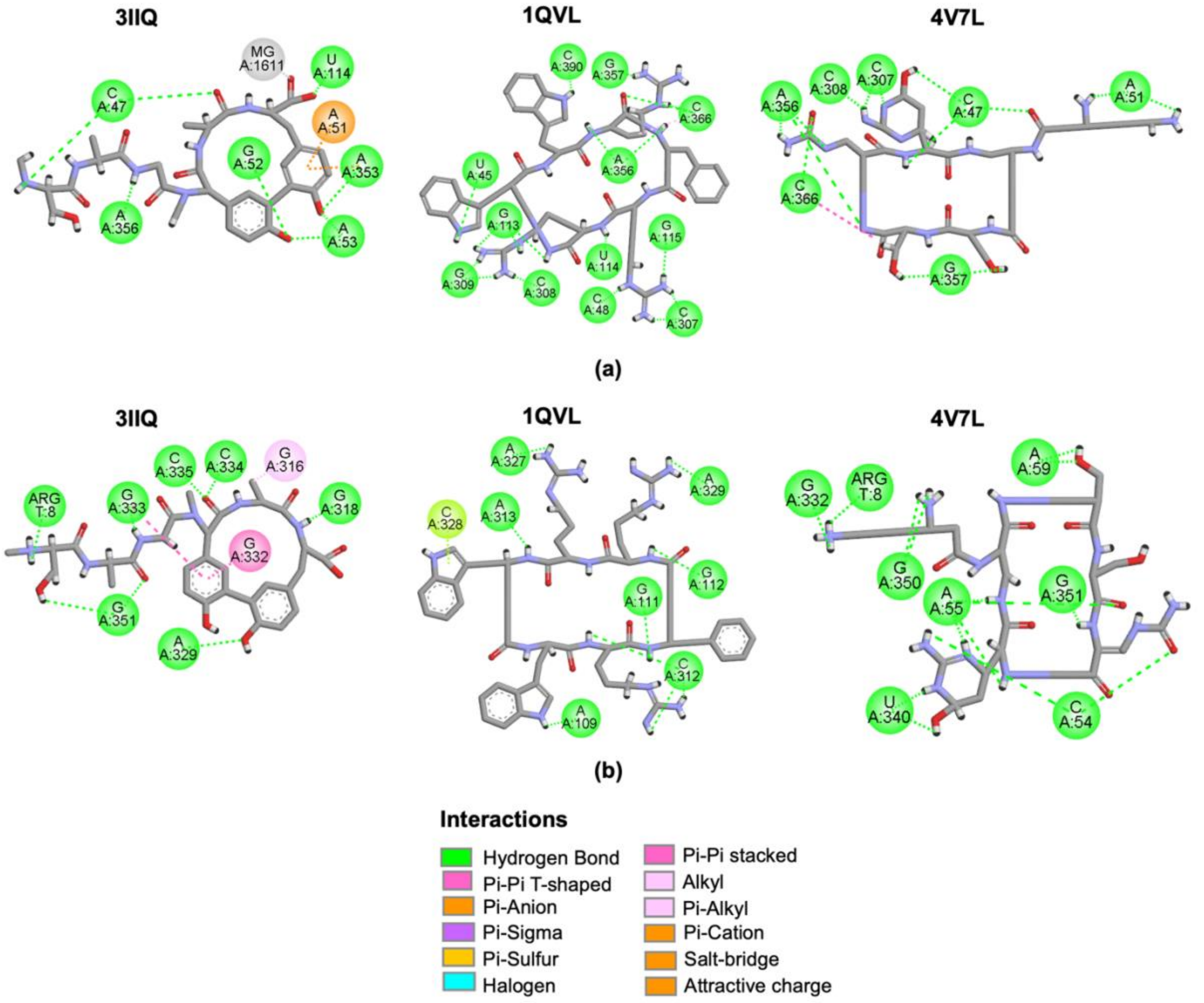

Figure S16. 2D interaction maps for DBAASP v3 hit peptides for the putative binding pocket obtained from **(a)** Glide SP-Peptide and **(b)** rDock docking. Only polar hydrogens are displayed on 2D interaction maps. The non-bonded interactions between the ligand and the surrounding nucleotides based on the color-codes are displayed with dashed lines on 2D maps.

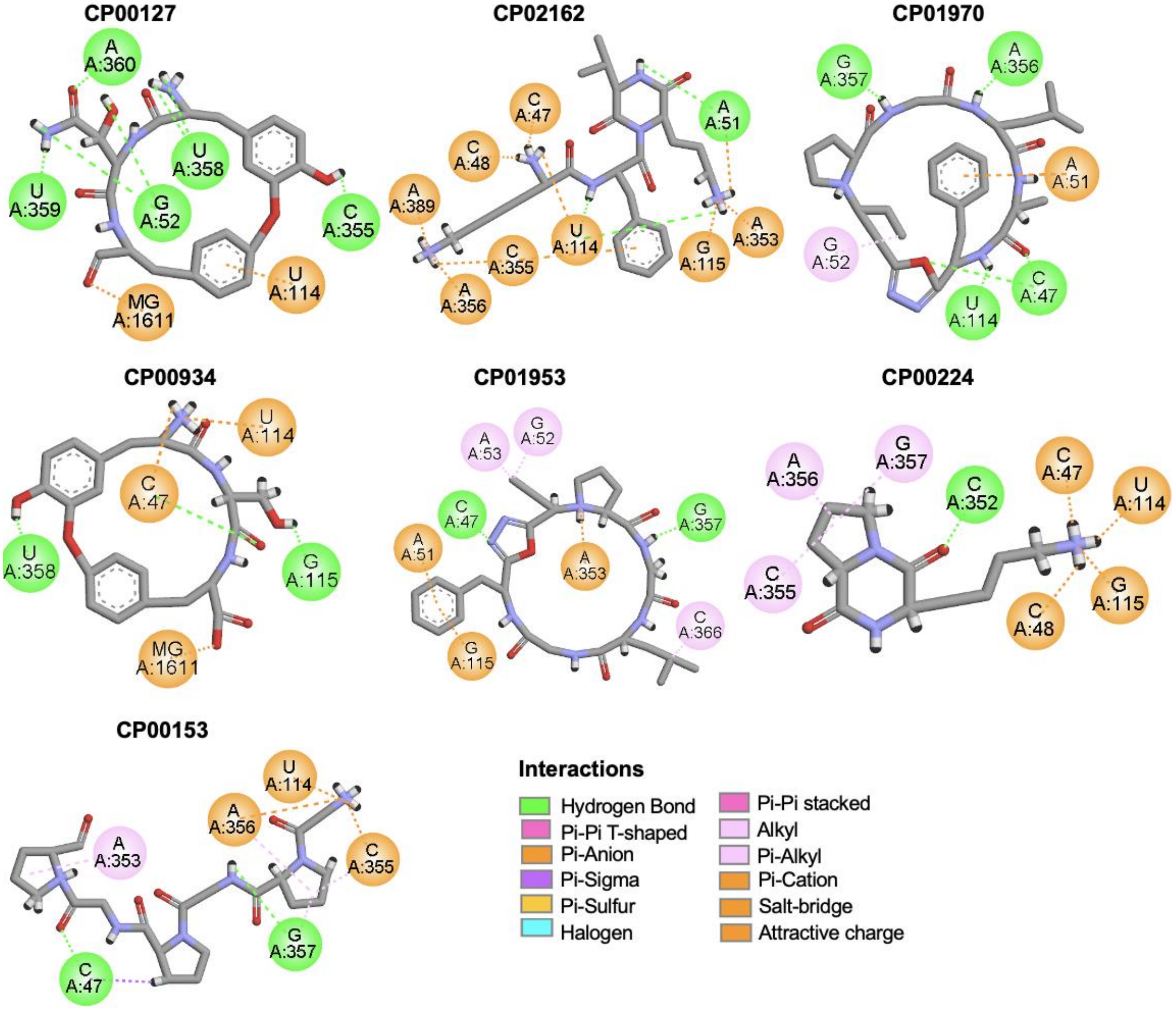

Figure S17. 2D interaction maps for CyclicPepedia hit peptides for the putative binding pocket obtained from Glide SP-Peptide docking. Only polar hydrogens are displayed on 2D interaction maps. The non-bonded interactions between the ligand and the surrounding nucleotides based on the color-codes are displayed with dashed lines on 2D maps.

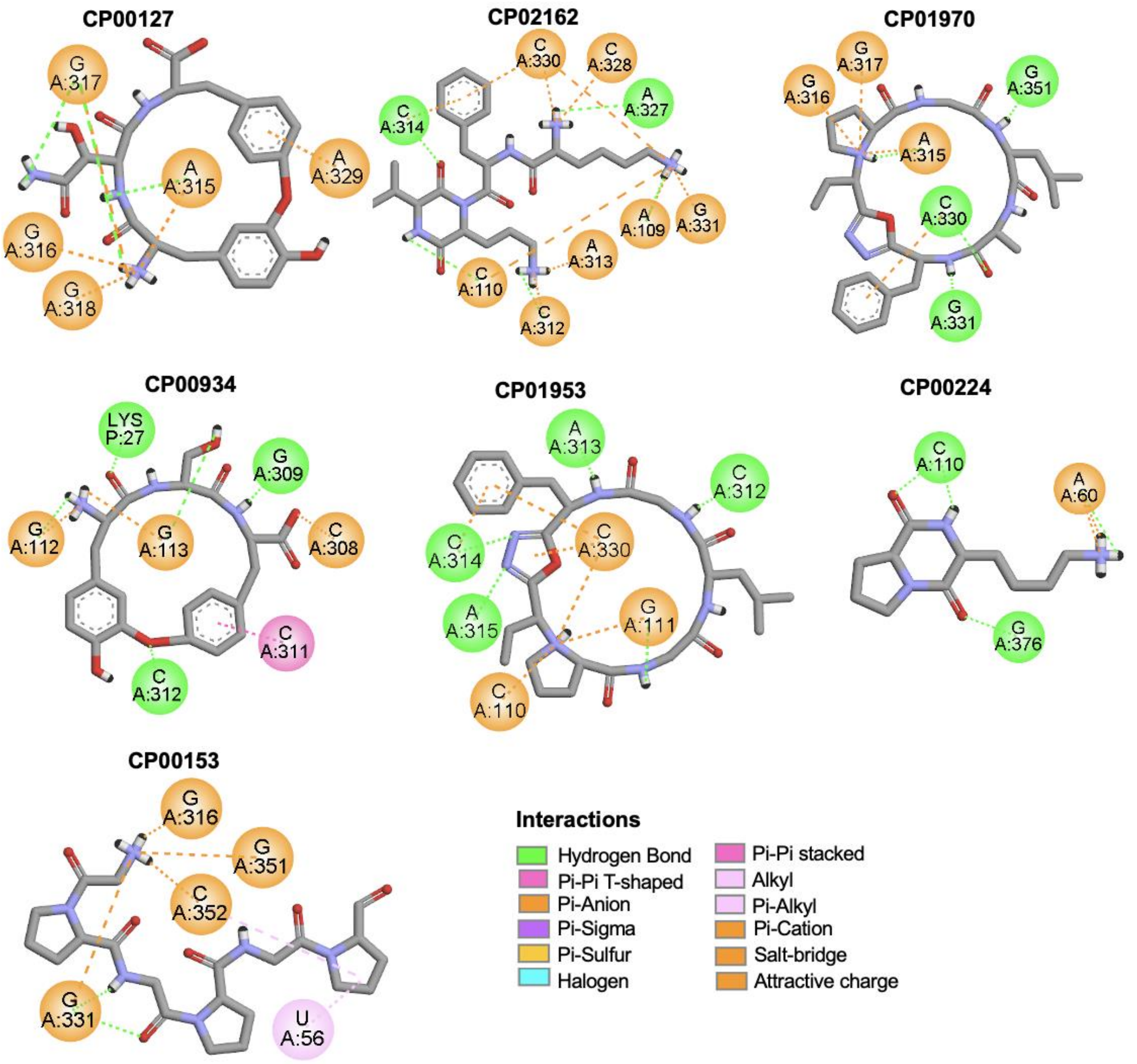

Figure S18. 2D interaction maps for CyclicPepedia hit peptides for the putative binding pocket obtained from rDock docking. Only polar hydrogens are displayed on 2D interaction maps. The non-bonded interactions between the ligand and the surrounding nucleotides based on the color-codes are displayed with dashed lines on 2D maps.

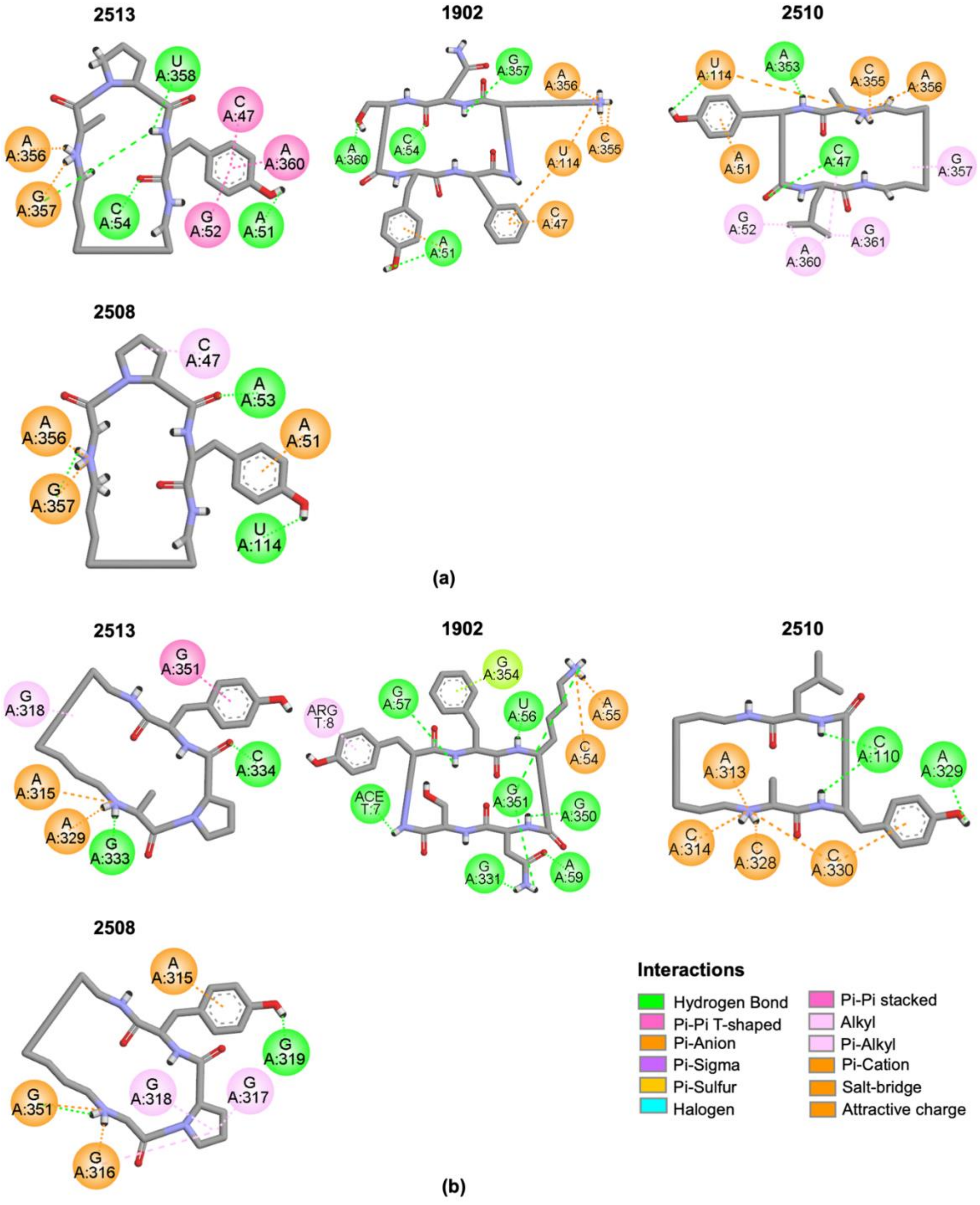

Figure S19. 2D interaction maps for CycPeptMPDB hit peptides for the putative binding pocket from **(a)** Glide SP-Peptide and **(b)** rDock docking. Only polar hydrogens are displayed on 2D interaction maps. The non-bonded interactions between the ligand and the surrounding nucleotides based on the color-codes are displayed with dashed lines on 2D maps.

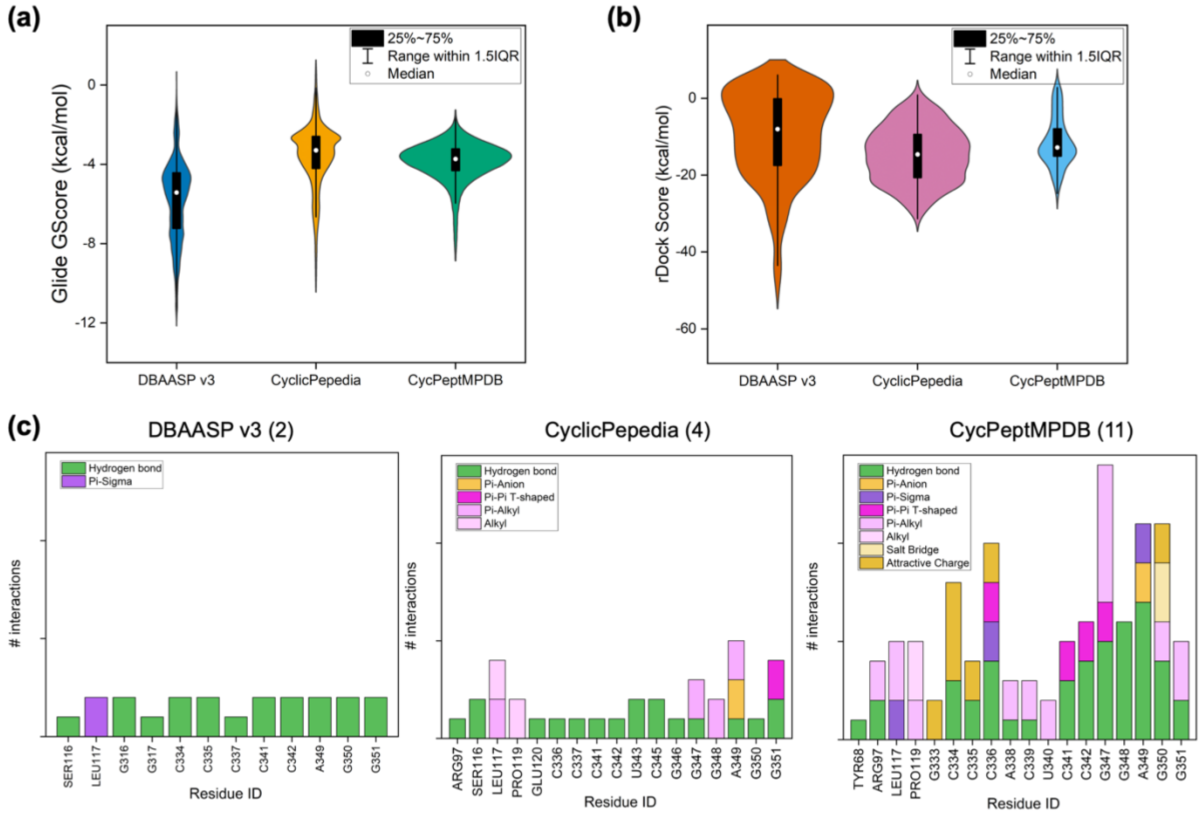

Figure S20. Comparative analysis of peptide datasets based on docking scores, amino acid positional frequencies, and 2D interaction profiles within the B8 bridge. **(a)** Violin plots of Glide GScore values and **(b)** rDock docking scores for peptides derived from the DBAASP v3, CyclicPepedia, and CycPeptMPDB databases. **(c)** Bar plots illustrating the frequency and types of peptide–ribosome interactions—including hydrogen bonds, π-interactions, and salt bridges—for selected peptides through consensus docking of Glide and rDock filtered for z-scores below -1.65.

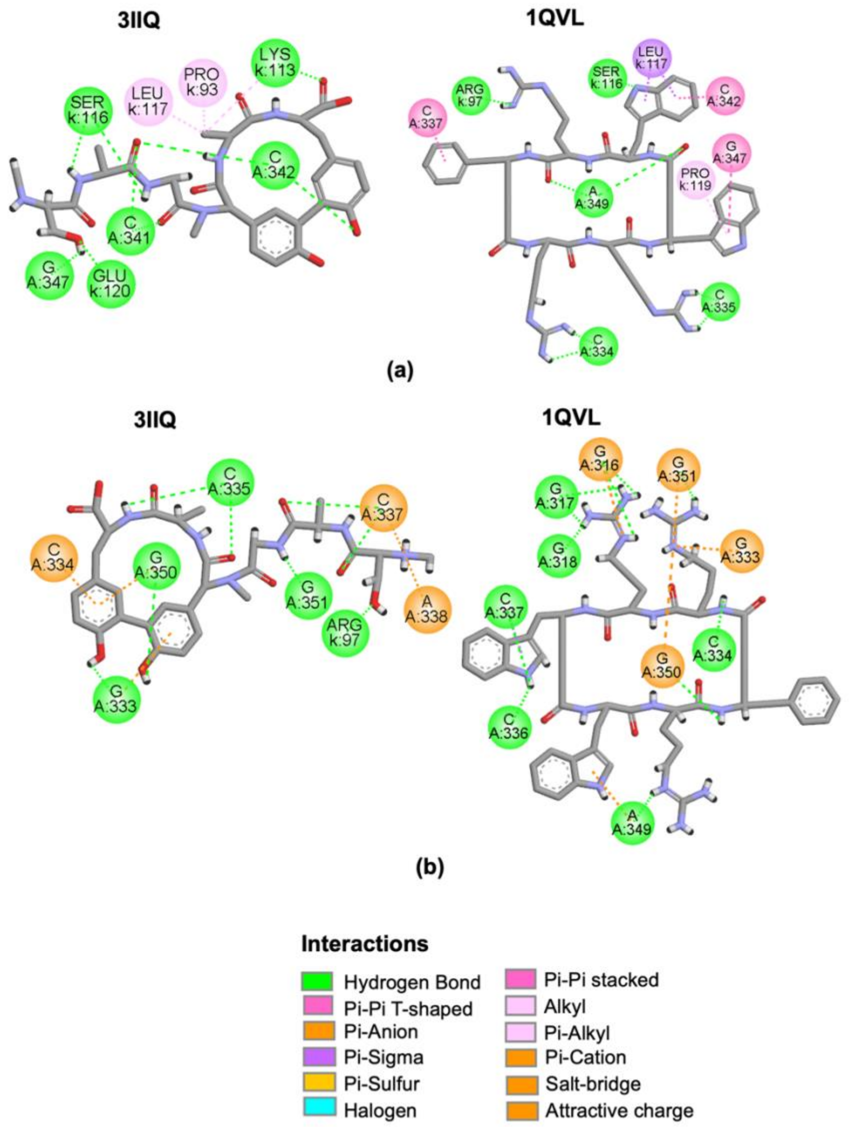

Figure S21. 2D interaction maps for DBAASP v3 hit peptides for the B8 inter-subunit bridge obtained from **(a)** Glide SP-Peptide and **(b)** rDock docking. Only polar hydrogens are displayed on 2D interaction maps. The non-bonded interactions between the ligand and the surrounding nucleotides based on the color-codes are displayed with dashed lines on 2D maps.

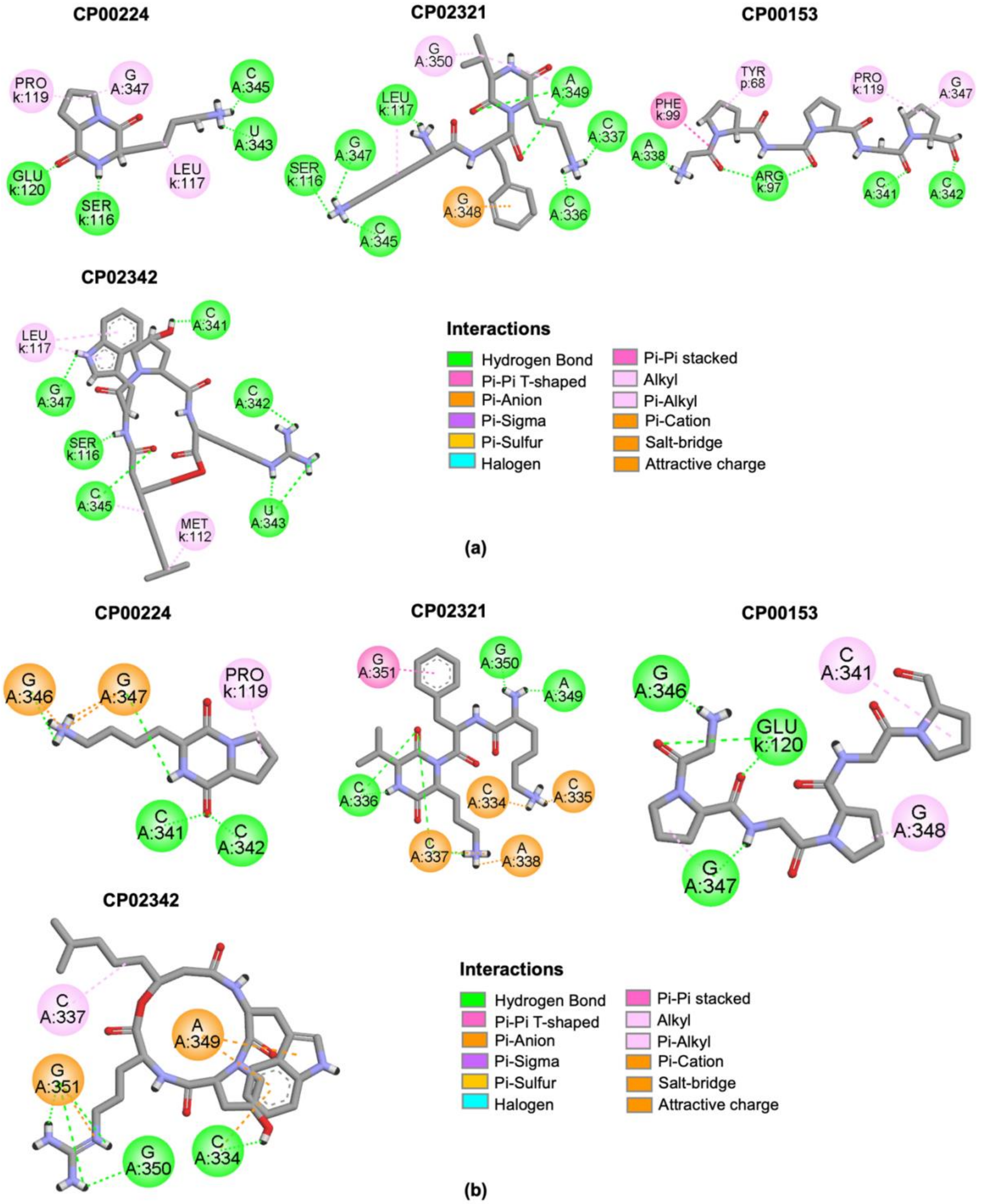

Figure S22. 2D interaction maps for CyclicPepedia hit peptides for the B8 inter-subunit bridge obtained from **(a)** Glide SP-Peptide and **(b)** rDock docking. Only polar hydrogens are displayed on 2D interaction maps. The non-bonded interactions between the ligand and the surrounding nucleotides based on the color-codes are displayed with dashed lines on 2D maps.

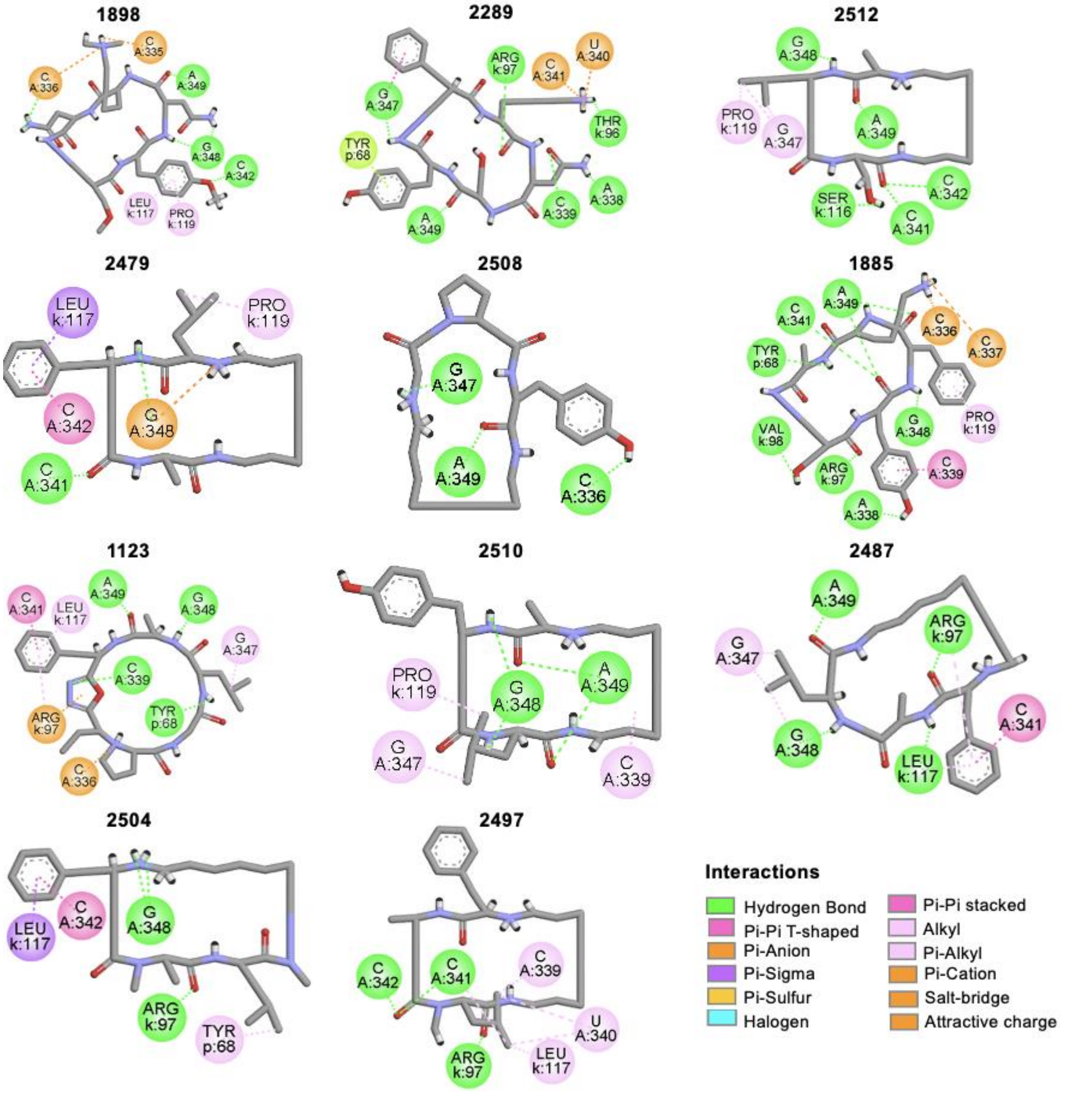

Figure S23. 2D interaction maps for CycPeptMPDB hit peptides for the B8 inter-subunit bridge obtained from Glide SP-Peptide docking. Only polar hydrogens are displayed on 2D interaction maps. The non-bonded interactions between the ligand and the surrounding nucleotides based on the color-codes are displayed with dashed lines on 2D maps.

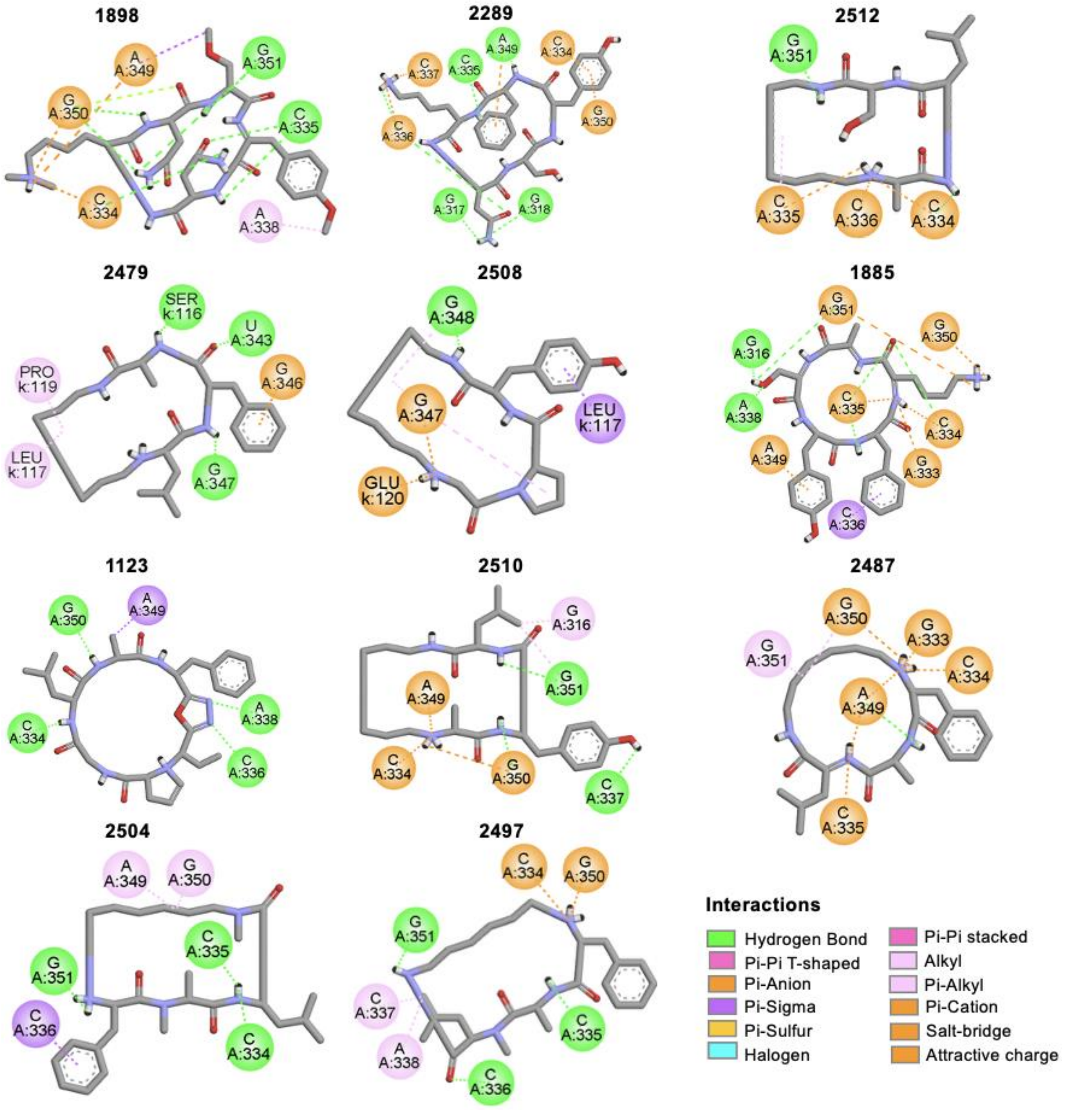

Figure S24. 2D interaction maps for CycPeptMPDB hit peptides for the B8 inter-subunit bridge obtained from rDock docking. Only polar hydrogens are displayed on 2D interaction maps. The non-bonded interactions between the ligand and the surrounding nucleotides based on the color-codes are displayed with dashed lines on 2D maps.

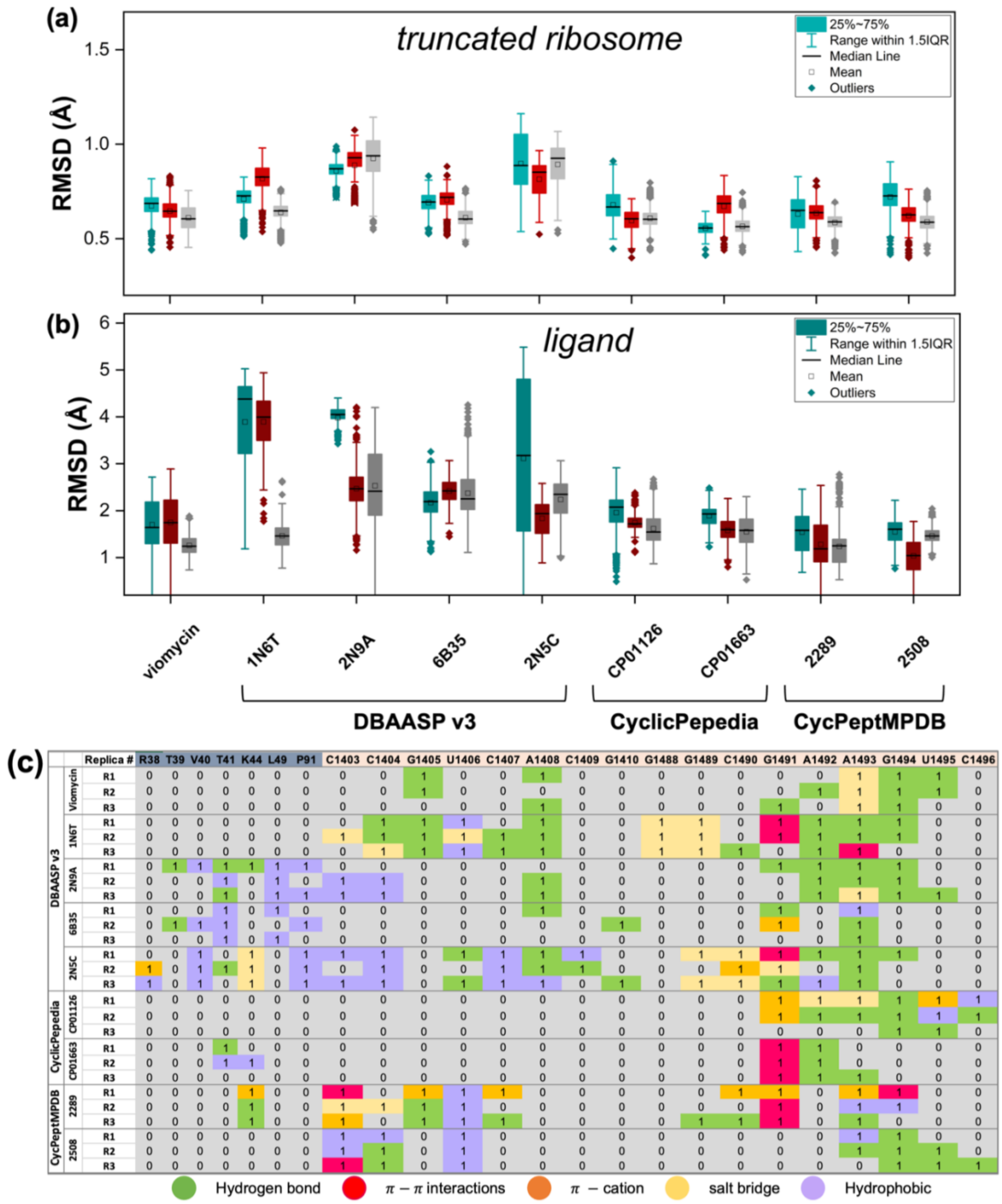

Figure S25. Root mean square deviation (RMSD) on all atoms of **(a)** truncated *E. coli* ribosome (PDB ID 4V7L) and **(b)** hit peptide molecules selected for **DC region** from three peptide libraries in 100 ns long MD simulations of three independent replicas. **(c)** Ribosome-peptide interaction fingerprinting for the native ligand viomycin and the selected 8 hit peptides from three peptide libraries in the DC region. Residues of the s12 ribosomal protein (blue) and nucleotides of the 16S rRNA (wheat) with high occurrence in non-bonded interactions with the peptides are indicated at the top. The involvement of these residues in non-bonded interactions is color-coded based on interaction type: hydrogen bond (green), π-π interaction (pink), π-cation (orange), salt-bridge (light orange), and hydrophobic (slate) interactions. Residues involved in non-bonded interactions observed in more than 30% of the 100 ns production runs are marked with '1', while their absence is marked with '0'.

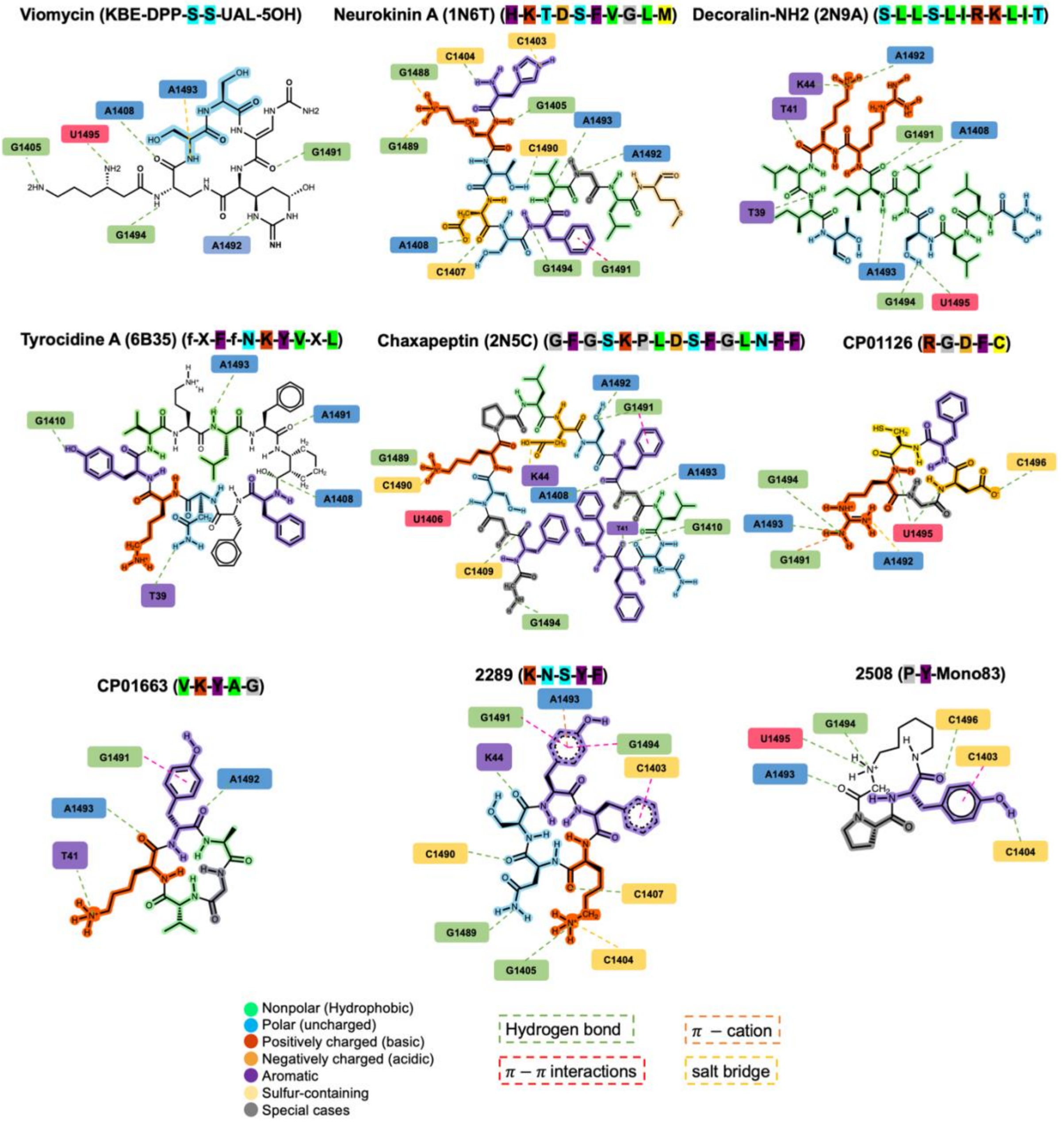

Figure S26. Interaction fingerprinting of co-crystalized peptide (viomycin) and the selected peptide candidates in 100 ns long MD simulations of three independent replicas within the DC cavity. 2D interactions are annotated as hydrogen bonds (green dashed lines), π–π interactions (red dashed lines), π–cation contacts (orange dashed lines), salt bridges (orange dashed lines). Amino-acid side chains are color-coded by physicochemical properties: nonpolar (green), polar uncharged (cyan), positively charged (dark orange), negatively charged (light orange), aromatic (purple), sulfur-containing (yellow), and special cases (grey). Nucleotides are color-coded as blue (adenine), green (guanine), yellow (cytosine), and magenta (uracil), while residues from protein s12 are shown in slate.

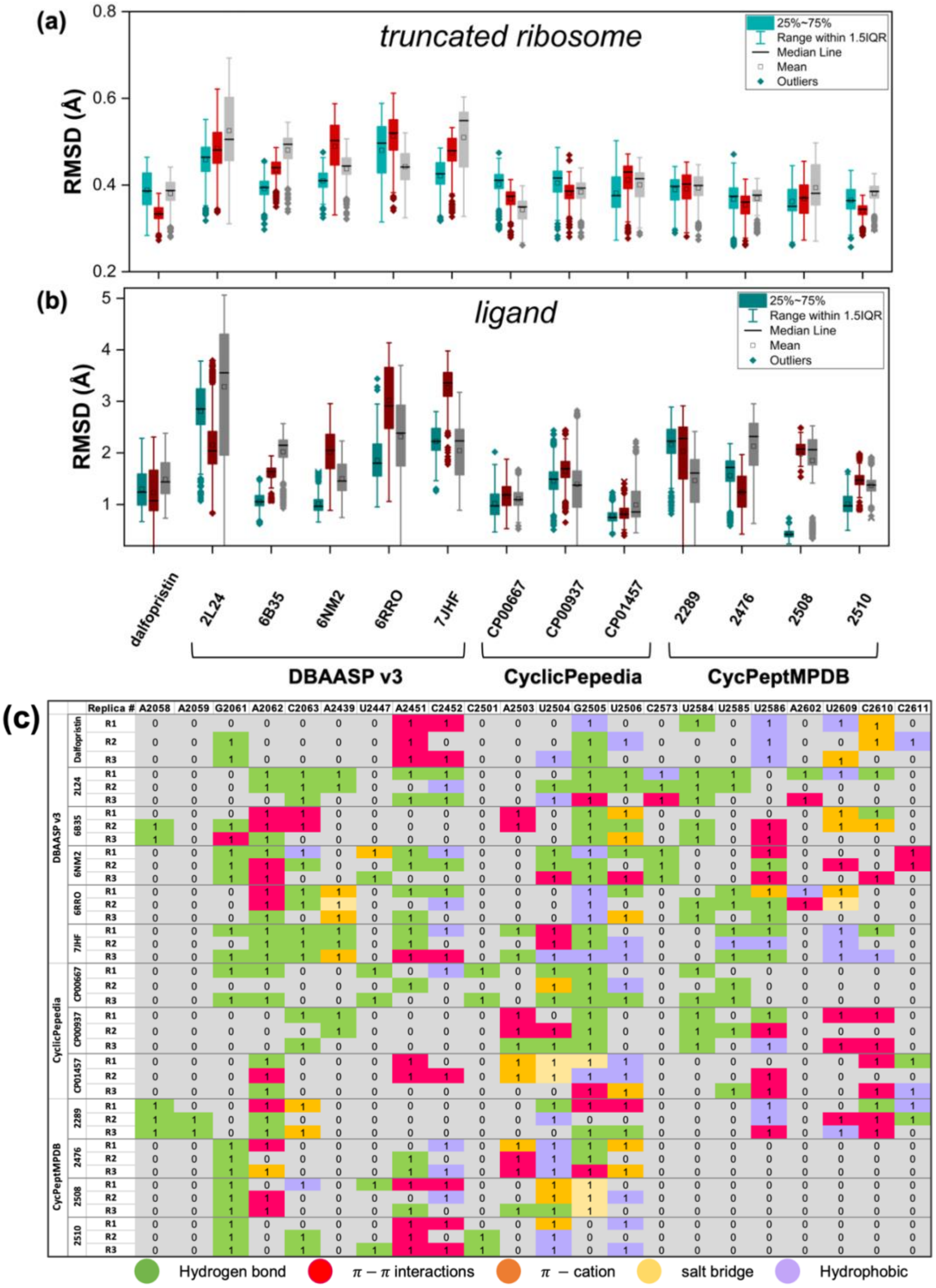

Figure S27. Root mean square deviation (RMSD) on all atoms of **(a)** truncated *E. coli* ribosome (PDB ID 4U24) and **(b)** hit peptide molecules selected for the **PTC region** from three peptide libraries in 100 ns long MD simulations of three independent replicas. **(c)** Ribosome-peptide interaction fingerprinting for the native ligand dalfopristin and the selected 12 hit peptides from three peptide libraries in the PTC region. Nucleotides of the 23S rRNA with high occurrence in non-bonded interactions with the peptides are indicated at the top. The involvement of these residues in non-bonded interactions is color-coded based on interaction type: hydrogen bond (green), π-π interaction (pink), π-cation (orange), salt-bridge (light orange), and hydrophobic (slate) interactions. Residues involved in non-bonded interactions observed in more than 30% of the 100 ns production runs are marked with '1', while their absence is marked with '0'.

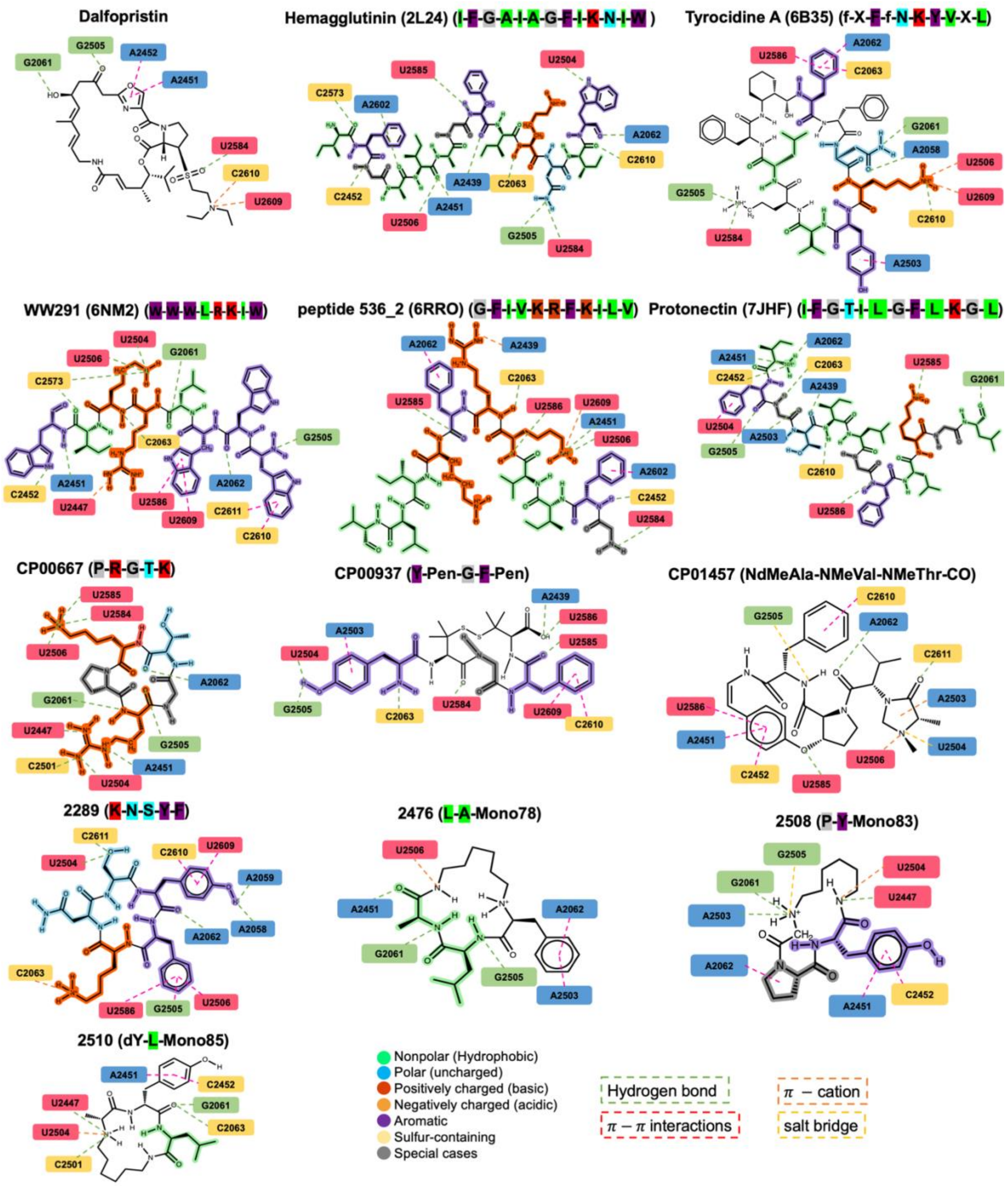

Figure S28. Interaction fingerprinting of co-crystalized peptide (dalfopristin) and the selected peptide candidates in 100 ns long MD simulations of three independent replicas within the PTC cavity. 2D interactions are annotated as hydrogen bonds (green dashed lines), π–π interactions (red dashed lines), π–cation contacts (orange dashed lines), salt bridges (orange dashed lines). Amino-acid side chains are color-coded by physicochemical properties: nonpolar (green), polar uncharged (cyan), positively charged (dark orange), negatively charged (light orange), aromatic (purple), sulfur-containing (yellow), and special cases (grey). Nucleotides are color-coded as blue (adenine), green (guanine), yellow (cytosine), and magenta (uracil).

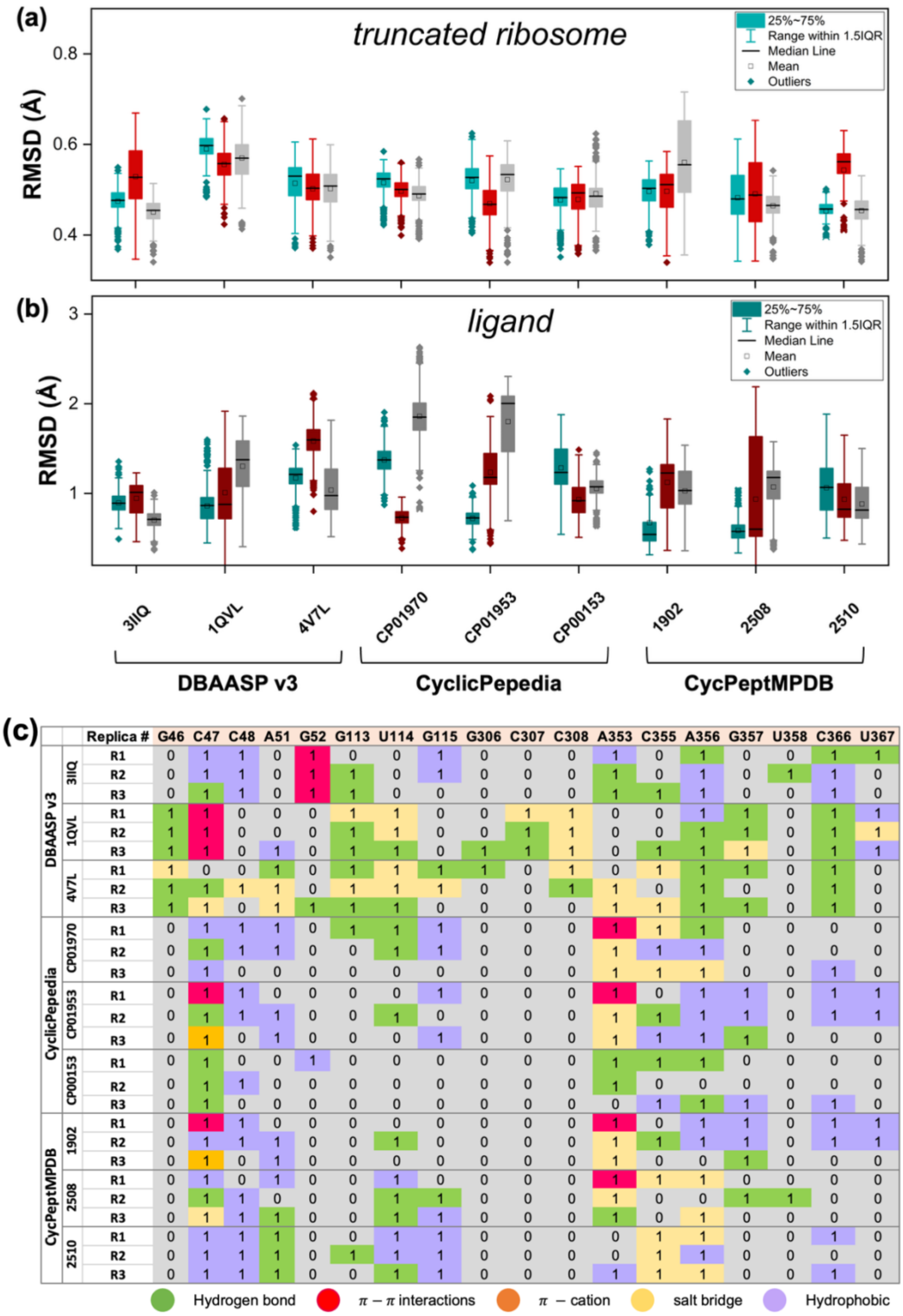

		Replica #	G46	C47	C48	A51	G52	G113	U114	G115	G306	C307	C308	A353	C355	A356	G357	U358	C366	U367
DBAASP v3	3IIQ	R1	0	1	1	0	1	0	0	1	0	0	0	1	0	1	0	0	1	1
		R2	0	1	1	0	1	1	0	1	0	0	0	1	0	1	0	1	1	0
		R3	0	1	1	0	1	1	0	0	0	0	0	1	1	1	0	0	1	0
	1QVL	R1	1	1	0	0	0	1	1	0	0	1	1	0	0	1	1	0	1	1
		R2	1	1	0	0	0	1	1	0	0	1	1	0	0	1	1	0	1	1
		R3	1	1	0	1	0	1	1	0	1	1	1	0	1	1	1	0	1	1
	4V7L	R1	1	0	0	1	0	1	1	1	1	0	1	0	1	1	1	0	1	0
		R2	1	1	1	1	0	1	1	1	0	0	1	1	0	1	0	0	1	0
		R3	1	1	0	1	1	1	1	0	0	0	0	1	1	1	1	0	1	0
CyclicPepedia	CP01970	R1	0	1	1	1	0	1	1	1	0	0	0	1	1	1	0	0	0	0
		R2	0	1	1	1	0	0	1	1	0	0	0	1	1	1	0	0	0	0
		R3	0	1	0	0	0	0	0	0	0	0	0	1	1	1	0	0	1	0
	CP01953	R1	0	1	1	0	0	0	0	1	0	0	0	1	0	1	1	0	1	1
		R2	0	1	1	1	0	0	1	0	0	0	0	1	1	1	1	0	1	1
		R3	0	1	0	1	0	0	0	1	0	0	0	1	1	1	1	0	0	0
	CP00153	R1	0	1	0	0	1	0	0	0	0	0	0	1	1	1	0	0	0	0
		R2	0	1	1	0	0	0	0	0	0	0	0	1	0	0	0	0	0	0
		R3	0	1	0	0	0	0	0	0	0	0	0	0	1	1	1	0	1	0
CycPeptMPDB	1902	R1	0	1	1	0	0	0	0	0	0	0	0	1	0	1	1	0	1	1
		R2	0	1	1	1	0	0	1	0	0	0	0	1	1	1	1	0	1	1
		R3	0	1	0	1	0	0	0	0	0	0	0	1	0	0	1	0	0	0
	2508	R1	0	1	0	1	0	0	1	0	0	0	0	1	1	1	0	0	0	0
		R2	0	1	1	0	0	0	1	1	0	0	0	1	0	0	1	1	0	0
		R3	0	1	1	1	0	0	1	1	0	0	0	1	0	1	0	0	0	0
	2510	R1	0	1	1	1	0	0	1	1	0	0	0	0	1	1	0	0	1	0
		R2	0	1	1	1	0	1	1	1	0	0	0	0	1	1	0	0	0	0
		R3	0	1	1	1	0	0	1	1	0	0	0	1	1	1	0	0	1	0

Figure S29. Root mean square deviation (RMSD) on all atoms of **(a)** truncated *E. coli* ribosome (PDB ID 4V7L) and **(b)** hit peptide molecules selected for **putative binding pocket** from three peptide libraries in 100 ns long MD simulations of three independent replicas. **(c)** Ribosome-peptide interaction fingerprinting for the selected 9 hit peptides from three peptide libraries in putative binding pocket. Nucleotides of the 16S rRNA (wheat) with high occurrence in non-bonded interactions with the peptides are indicated at the top. The involvement of these residues in non-bonded interactions is color-coded based on interaction type: hydrogen bond (green), π-π interaction (pink), π-cation (orange), salt-bridge (light orange), and hydrophobic (slate) interactions. Residues involved in non-bonded interactions observed in more than 30% of the 100 ns production runs are marked with '1', while their absence is marked with '0'.

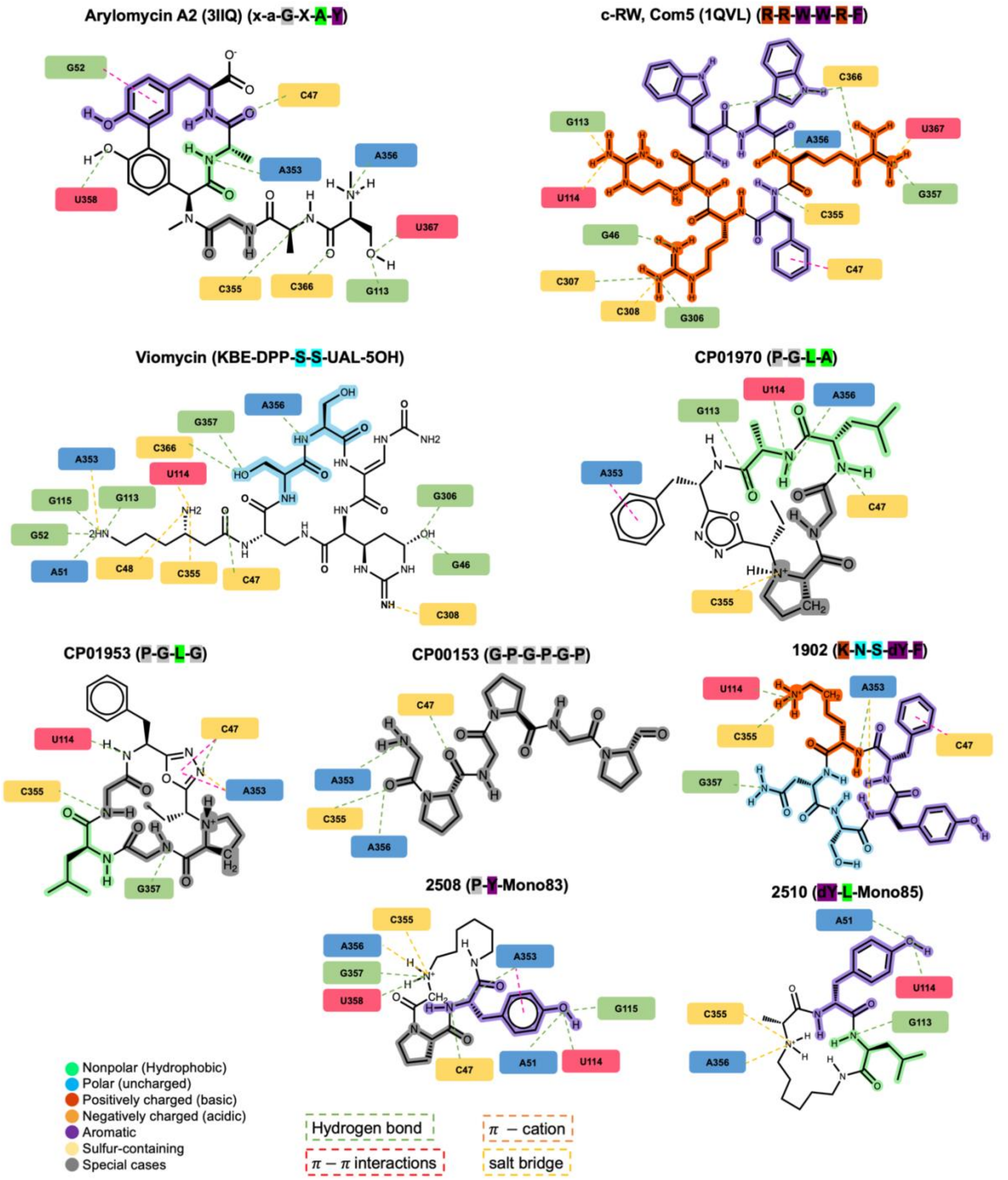

Figure S30. Interaction fingerprinting of the selected peptide candidates in 100 ns long MD simulations of three independent replicas within the putative binding pocket. 2D interactions are annotated as hydrogen bonds (green dashed lines), π–π interactions (red dashed lines), π–cation contacts (orange dashed lines), salt bridges (orange dashed lines). Amino-acid side chains are color-coded by physicochemical properties: nonpolar (green), polar uncharged (cyan), positively charged (dark orange), negatively charged (light orange), aromatic (purple), sulfur-containing (yellow), and special cases (grey). Nucleotides are color-coded as blue (adenine), green (guanine), yellow (cytosine), and magenta (uracil).

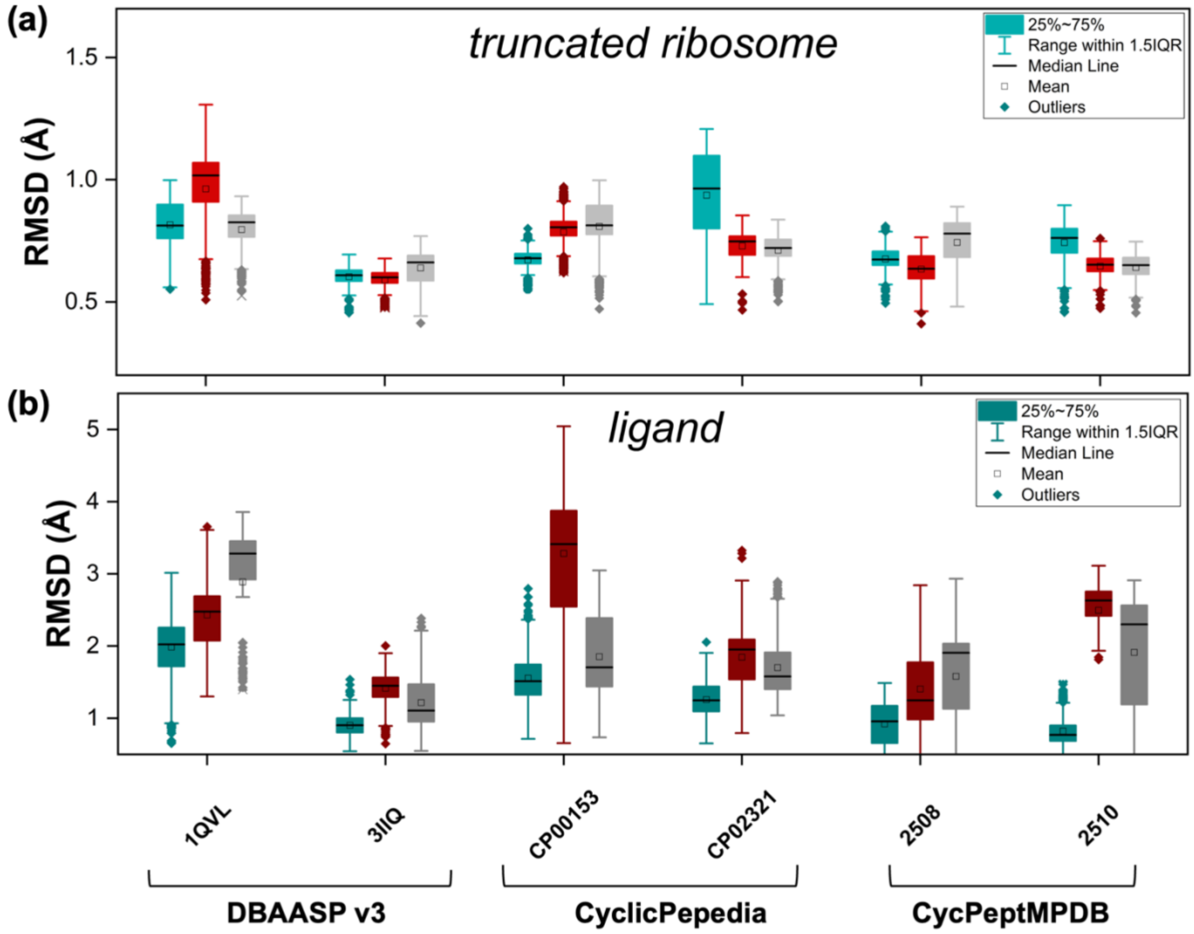

(c)

		Replica #	Y68	E92	R97	G100	R107	K113	V115	S116	A118	P119	G333	C334	C335	C336	C337	C339	C341	C342	G347	G348	A349	G350
DBAASP v3	1QVL	R1	1	0	1	0	0	0	0	0	0	0	1	1	1	1	1	1	1	0	0	1	1	1
		R2	1	0	1	0	0	0	0	1	0	1	1	1	0	1	1	1	0	0	1	1	1	0
		R3	1	0	1	0	0	0	0	0	0	1	1	1	1	1	1	1	1	0	0	1	1	1
	3IIQ	R1	0	1	0	0	1	1	0	1	1	1	0	0	0	0	0	0	1	1	1	0	0	0
		R2	0	1	0	0	1	1	1	1	1	0	0	0	0	0	0	0	1	1	0	0	0	0
		R3	0	0	0	0	0	1	1	1	1	0	0	0	0	0	0	0	1	1	1	0	0	0
CyclicPepedia	CP00153	R1	1	0	0	1	0	0	0	0	0	1	0	0	0	0	1	0	0	0	1	0	0	0
		R2	1	0	1	1	0	0	0	0	0	0	0	0	0	1	1	0	0	0	0	0	0	0
		R3	1	0	1	1	0	0	0	0	0	1	0	0	0	0	0	1	0	0	0	0	1	0
	CP02321	R1	1	0	1	0	0	0	0	1	1	1	0	0	0	0	0	0	0	0	1	1	1	1
		R2	1	0	0	0	0	0	1	0	1	1	0	0	1	1	1	0	0	0	1	1	1	1
		R3	1	0	0	0	0	0	1	1	1	0	0	0	0	1	0	0	0	0	1	1	0	0
CycPeptMPDB	2508	R1	1	0	1	0	0	0	0	0	1	1	0	0	0	1	1	0	0	0	0	0	0	0
		R2	1	0	0	0	0	0	0	1	1	1	0	0	0	0	0	0	0	0	1	1	0	0
		R3	1	0	1	0	0	0	0	1	0	1	0	0	0	0	0	0	0	0	1	1	0	0
	2510	R1	1	0	0	0	0	0	0	0	0	1	0	0	1	1	1	0	0	0	0	0	0	0
		R2	1	0	0	0	0	0	0	0	0	0	0	0	0	1	1	0	0	0	0	0	0	0
		R3	1	0	0	0	0	0	0	0	0	1	0	0	1	1	0	0	0	0	0	0	0	0

Hydrogen bond | $\pi-\pi$ interactions | π − cation | salt bridge | Hydrophobic

Figure S31. Root mean square deviation (RMSD) on all atoms of **(a)** truncated *E. coli* ribosome (PDB ID 4V7L) and **(b)** hit peptide molecules selected for **B8 bridge** from three peptide libraries in 100 ns long MD simulations of three independent replicas. **(c)** Ribosome-peptide interaction fingerprinting for the selected 6 hit peptides from three peptide libraries in B8 bridge. Residues of the L14 ribosomal protein (yellow), L19 ribosomal protein (orange), and nucleotides of the 16S rRNA (purple) with high occurrence in non-bonded interactions with the peptides are indicated at the top. The involvement of these residues in non-bonded interactions is color-coded based on interaction type: hydrogen bond (green), π-π interaction (pink), π-cation (orange), salt-bridge (light orange), and hydrophobic (slate) interactions. Residues involved in non-bonded interactions observed in more than 30% of the 100 ns production runs are marked with '1', while their absence is marked with '0'.

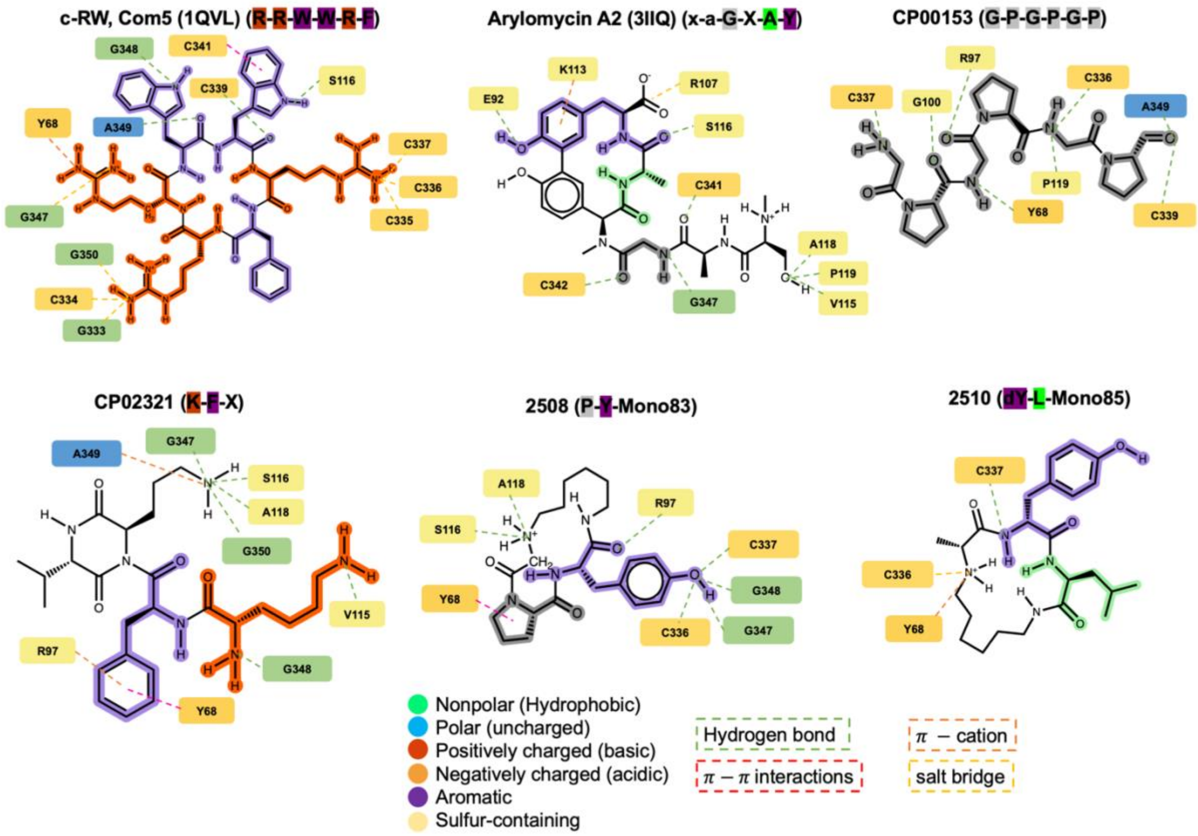

Figure S32. Interaction fingerprinting of the selected peptide candidates in 100 ns long MD simulations of three independent replicas within the B8 bridge region. 2D interactions are annotated as hydrogen bonds (green dashed lines), π–π interactions (red dashed lines), π–cation contacts (orange dashed lines), salt bridges (orange dashed lines). Amino-acid side chains are color-coded by physicochemical properties: nonpolar (green), polar uncharged (cyan), positively charged (dark orange), negatively charged (light orange), aromatic (purple), sulfur-containing (yellow), and special cases (grey). Nucleotides are color-coded as blue (adenine), green (guanine), yellow (cytosine), and magenta (uracil).

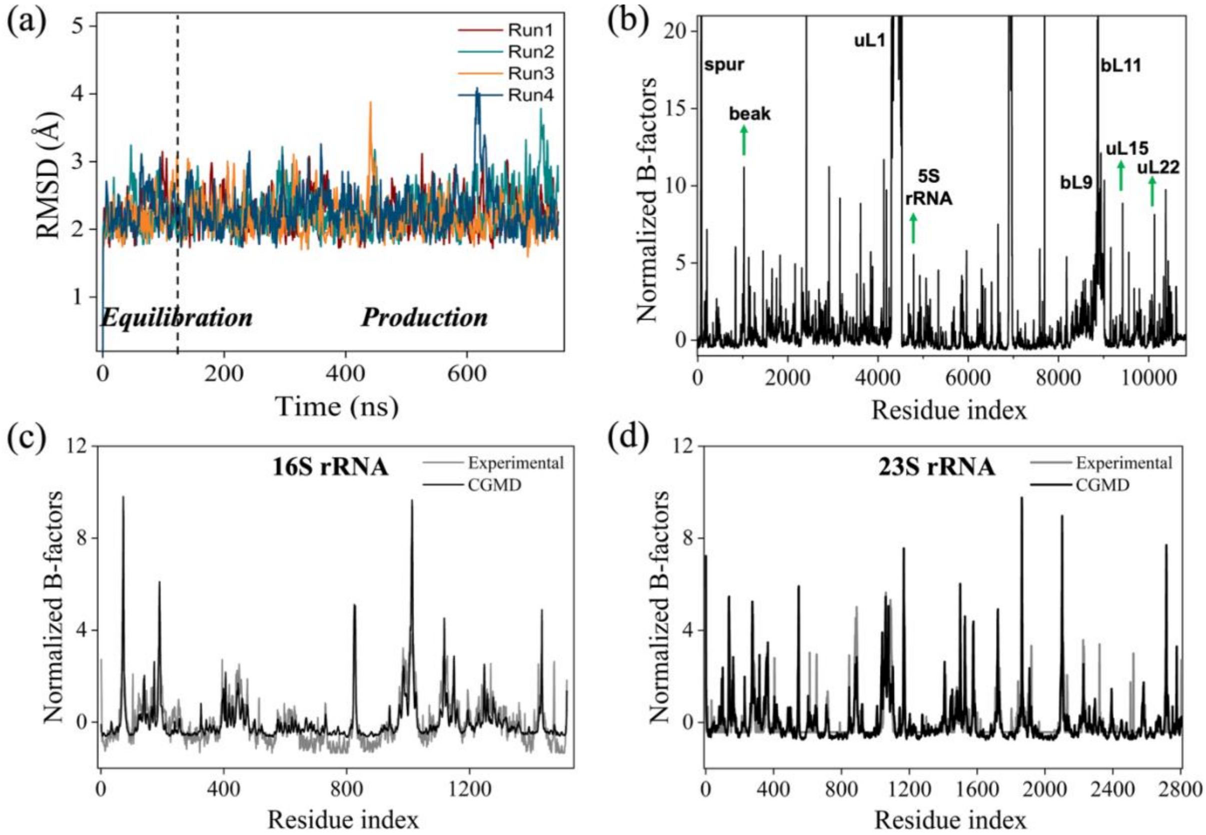

Figure S33. Conformational stability and flexibility of the *E. coli* 70S ribosome in 750 ns long CGMD simulations. (a) Root mean squared deviation (RMSD) and (b) theoretical normalized B-factor profiles for the CGMD simulations of *E. coli* 70S complex (PDB ID: 4v5h). Experimental and theoretical normalized B-factors for *E. coli* (c) 16S rRNA and (d) 23S rRNA. Theoretical B-factors were calculated from CGMD-derived RMSF values and normalized using Z-score transformation ($B_i^{norm} = (B_i - \mu)/\sigma$) for direct comparison with experimental B-factors from the high-resolution structure PDB ID: 4v9d.

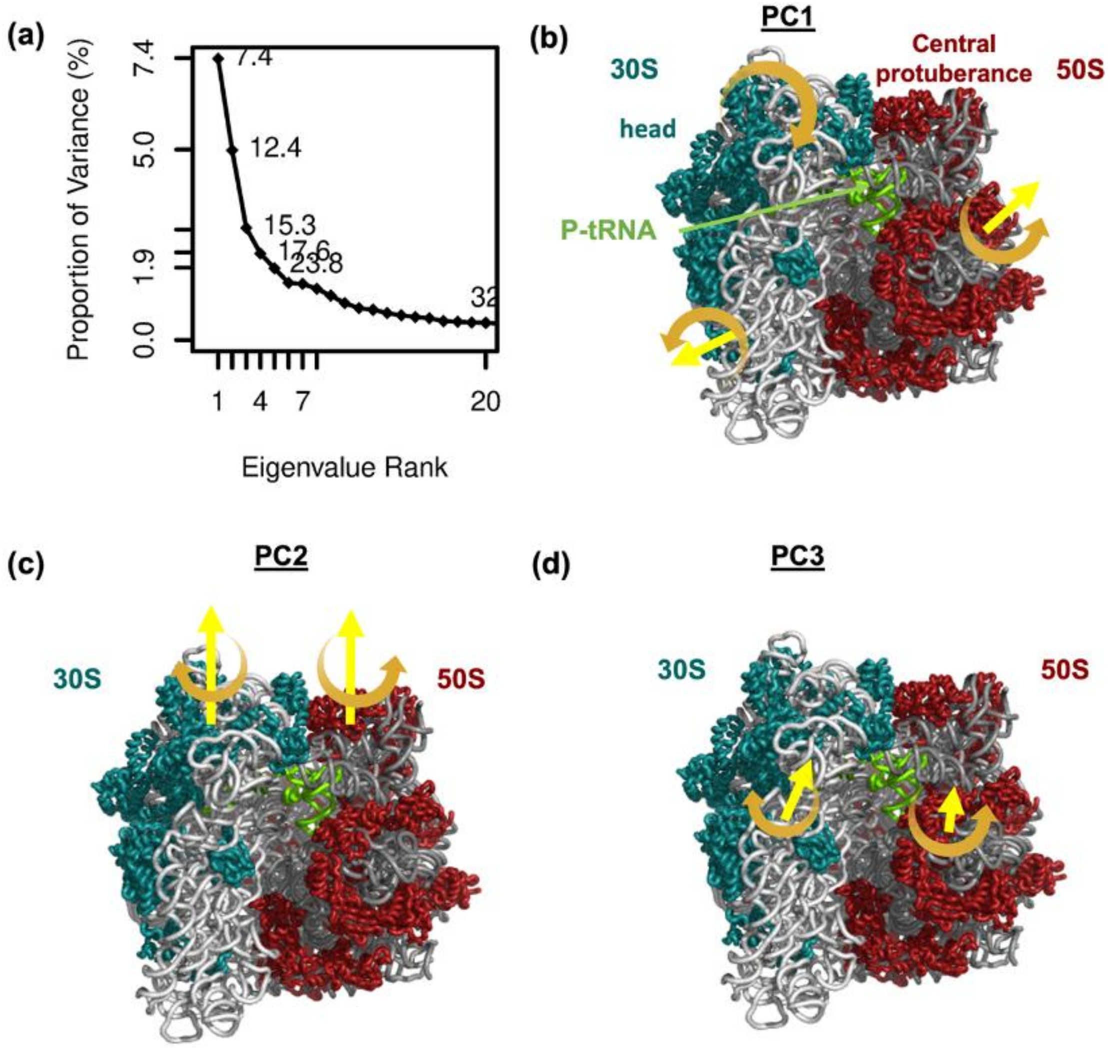

Figure S34. Principal component analysis of CGMD concatenated trajectories for the *E. coli* 70S ribosome (PDB ID: 4v5h) where the L1 stalk was excluded and aligned on the initial structure of the 50S core atoms. **(a)** Scree plot showing the variance explained by the first 20 principal components. **(b)–(d)** Representative global motions along PC1 (7.38%), PC2 (4.98%), and PC3 (2.94%). Arrows in orange indicate one direction of motion of the structural components around a rotational axis in yellow. PC1 captures the characteristic ratchet-like rotation of the subunits, while PC2 and PC3 capture the opening/closing dynamics of the subunit interface.

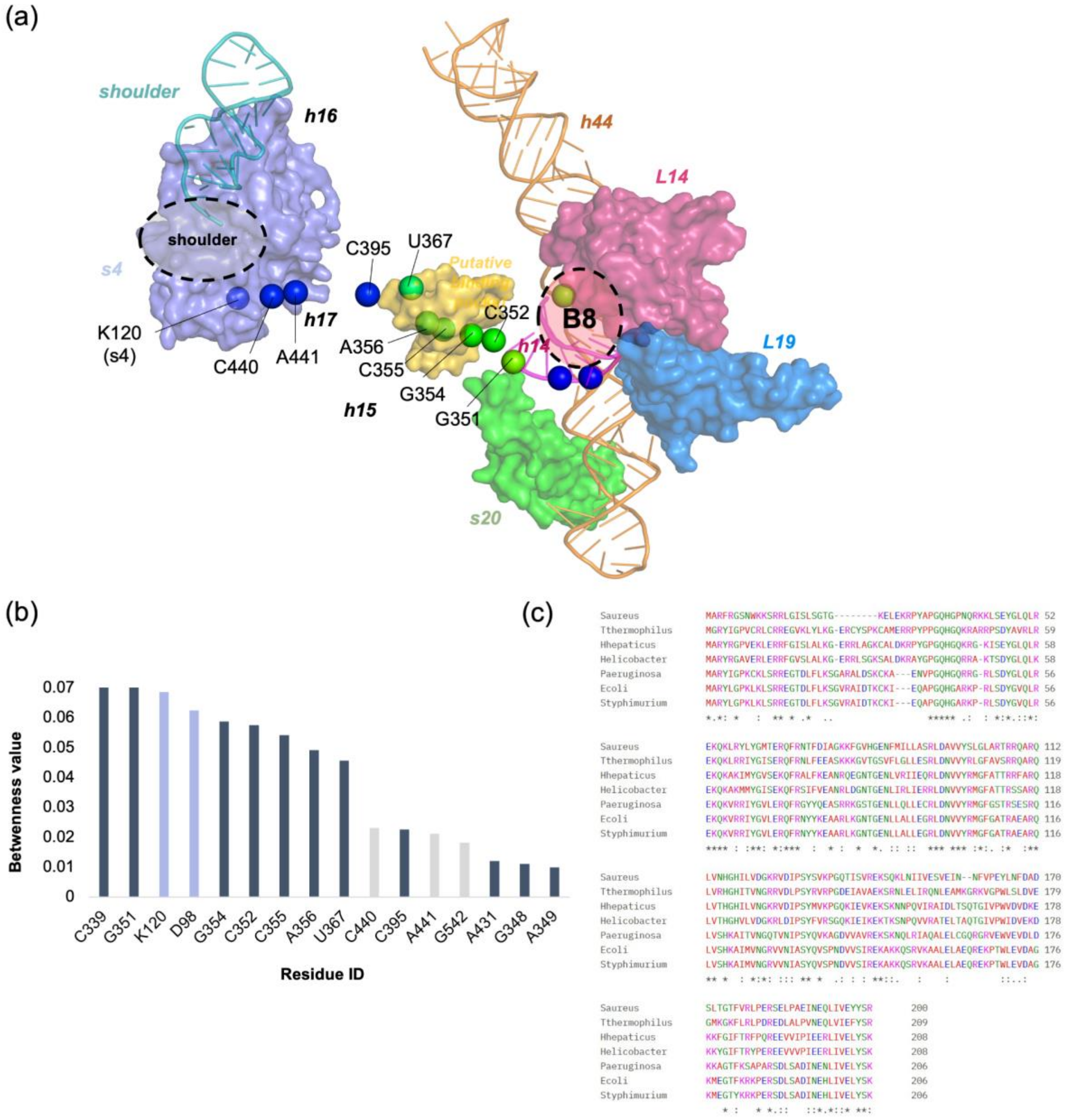

Figure S35. (a) Hub residues between the B8 bridge and the shoulder regions, shown in spheres colored according to their betweenness scores. (b) Evolutionary conservation (with CRW 2.0[5]) and network centrality of residues linking the B8 inter-subunit bridge and the shoulder. The bar chart displays the betweenness centrality values for the hub residues. Blue and light grey bars represent highly conserved residues (>98% conservation) and residues with lower conservation (<80%), respectively. Purple bars highlight residues belonging to the s4 ribosomal protein. (c) Conservation analysis for ribosomal protein s4 using Clustal W[6] for different bacterial species.

REFERENCES

(1) Halgren, T. A. Identifying and Characterizing Binding Sites and Assessing Druggability. *Journal of Chemical Information and Modeling* **2009**, *49* (2), 377–389.

(2) Friesner, R. A.; Banks, J. L.; Murphy, R. B.; Halgren, T. A.; Klicic, J. J.; Mainz, D. T.; Repasky, M. P.; Knoll, E. H.; Shelley, M.; Perry, J. K.; Shaw, D. E.; Francis, P.; Shenkin, P. S. Glide: A New Approach for Rapid, Accurate Docking and Scoring. 1. Method and Assessment of Docking Accuracy. *Journal of Medicinal Chemistry* **2004**, *47* (7), 1739–1749.

(3) Ruiz-Carmona, S.; Alvarez-Garcia, D.; Foloppe, N.; Garmendia-Doval, A. B.; Juhos, S.; Schmidtke, P.; Barril, X.; Hubbard, R. E.; Morley, S. D. rDock: A Fast, Versatile and Open Source Program for Docking Ligands to Proteins and Nucleic Acids. *PLoS Computational Biology* **2014**, *10* (4), e1003571.

(4) Kurkcuoglu, O.; Gunes, M. U.; Haliloglu, T. Local and Global Motions Underlying Antibiotic Binding in Bacterial Ribosome. *Journal of Chemical Information and Modeling* **2020**, *60* (12), 6447–6461.

(5) Cannone, J. J.; Subramanian, S.; Schnare, M. N.; Collett, J. R.; D'Souza, L. M.; Du, Y.; Feng, B.; Lin, N.; Madabusi, L. V.; Müller, K. M.; Pande, N.; Shang, Z.; Yu, N.; Gutell, R. R. The Comparative RNA Web (CRW) Site: An Online Database of Comparative Sequence and Structure Information for Ribosomal, Intron, and Other RNAs. *BMC Bioinformatics* **2002**, *3* (1), 2.

(6) Thompson, J. D.; Higgins, D. G.; Gibson, T. J. CLUSTAL W: Improving the Sensitivity of Progressive Multiple Sequence Alignment through Sequence Weighting, Position-Specific Gap Penalties and Weight Matrix Choice. *Nucleic Acids Research* **1994**, *22* (22), 4673–4680.
